# LDIP cooperates with SEIPIN and LDAP to facilitate lipid droplet biogenesis in Arabidopsis

**DOI:** 10.1093/plcell/koab179

**Published:** 2021-06-09

**Authors:** Michal Pyc, Satinder K. Gidda, Damien Seay, Nicolas Esnay, Franziska K. Kretzschmar, Yingqi Cai, Nathan M. Doner, Michael S. Greer, J. Joe Hull, Denis Coulon, Claire Bréhélin, Olga Yurchenko, Jan de Vries, Oliver Valerius, Gerhard H. Braus, Till Ischebeck, Kent D. Chapman, John M. Dyer, Robert T. Mullen

**Affiliations:** Department of Molecular and Cellular Biology, University of Guelph, Guelph, ON, N1G 2W1, Canada; Department of Molecular and Cellular Biology, University of Guelph, Guelph, ON, N1G 2W1, Canada; U.S. Department of Agriculture, Agricultural Research Service, U.S. Arid-Land Agricultural Research Center, Maricopa, Arizona 85138, USA; BioDiscovery Institute and Department of Biological Sciences, University of North Texas, Denton, Texas 76203, USA; Department of Plant Biochemistry, Albrecht-von-Haller-Institute for Plant Sciences, University of Göttingen, 37077 Göttingen, Germany; BioDiscovery Institute and Department of Biological Sciences, University of North Texas, Denton, Texas 76203, USA; Department of Molecular and Cellular Biology, University of Guelph, Guelph, ON, N1G 2W1, Canada; BioDiscovery Institute and Department of Biological Sciences, University of North Texas, Denton, Texas 76203, USA; U.S. Department of Agriculture, Agricultural Research Service, U.S. Arid-Land Agricultural Research Center, Maricopa, Arizona 85138, USA; Université de Bordeaux, Centre National de la Recherche Scientifique, Laboratoire de Biogenèse Membranaire, UMR5200, F-33140 Villenave d’Ornon, France; Université de Bordeaux, Centre National de la Recherche Scientifique, Laboratoire de Biogenèse Membranaire, UMR5200, F-33140 Villenave d’Ornon, France; U.S. Department of Agriculture, Agricultural Research Service, U.S. Arid-Land Agricultural Research Center, Maricopa, Arizona 85138, USA; Institute for Microbiology and Genetics, Göttingen Center for Molecular Biosciences and Campus Institute Data Science, Department of Applied Bioinformatics, University of Göttingen, 37077 Göttingen, Germany; Institute for Microbiology and Genetics and Göttingen Center for Molecular Biosciences, Department for Molecular Microbiology and Genetics, University of Göttingen, 37077 Göttingen, Germany; Institute for Microbiology and Genetics and Göttingen Center for Molecular Biosciences, Department for Molecular Microbiology and Genetics, University of Göttingen, 37077 Göttingen, Germany; Department of Plant Biochemistry, Albrecht-von-Haller-Institute for Plant Sciences, University of Göttingen, 37077 Göttingen, Germany; BioDiscovery Institute and Department of Biological Sciences, University of North Texas, Denton, Texas 76203, USA; U.S. Department of Agriculture, Agricultural Research Service, U.S. Arid-Land Agricultural Research Center, Maricopa, Arizona 85138, USA; Department of Molecular and Cellular Biology, University of Guelph, Guelph, ON, N1G 2W1, Canada

## Abstract

Cytoplasmic lipid droplets (LDs) are evolutionarily conserved organelles that store neutral lipids and play critical roles in plant growth, development, and stress responses. However, the molecular mechanisms underlying their biogenesis at the endoplasmic reticulum (ER) remain obscure. Here we show that a recently identified protein termed LD-associated protein [LDAP]-interacting protein (LDIP) works together with both endoplasmic reticulum-localized SEIPIN and the LD-coat protein LDAP to facilitate LD formation in *Arabidopsis thaliana*. Heterologous expression in insect cells demonstrated that LDAP is required for the targeting of LDIP to the LD surface, and both proteins are required for the production of normal numbers and sizes of LDs in plant cells. LDIP also interacts with SEIPIN via a conserved hydrophobic helix in SEIPIN and LDIP functions together with SEIPIN to modulate LD numbers and sizes in plants. Further, the co-expression of both proteins is required to restore normal LD production in *SEIPIN*-deficient yeast cells. These data, combined with the analogous function of LDIP to a mammalian protein called LD Assembly Factor 1, are discussed in the context of a new model for LD biogenesis in plant cells with evolutionary connections to LD biogenesis in other eukaryotes.

## Teaser

The lipid droplet (LD) proteins LDIP and LDAP cooperate with endoplasmic reticulum-localized SEIPIN to coordinate LD formation in plant cells.

##  

**Figure:**
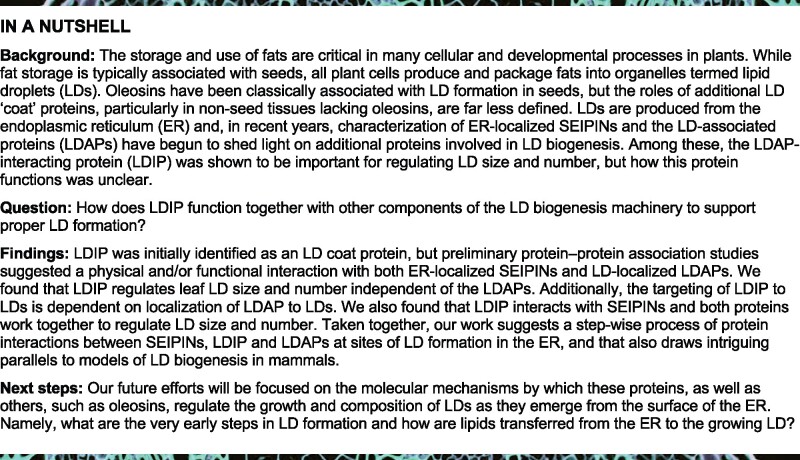


## Introduction

Cytoplasmic lipid droplets (LDs) are organelles that store neutral lipids, such as triacylglycerols (TAGs) and sterol esters, in a wide range of both unicellular and multicellular organisms (Yang et al., 2012a; Gross and Silver, 2014; Chapman et al., 2019; Ischebeck et al., 2020; Lundquist et al., 2020). Structurally, LDs are uniquely delineated by just a single phospholipid monolayer and coated with various proteins, which are broadly divided into two classes: class I LD proteins that target the LD surface by routing through the endoplasmic reticulum (ER), the site of LD formation; and class II LD proteins that target directly the LD surface from the cytoplasm (Kory et al., 2016). Studies from many eukaryotic organisms have increased our understanding of the types of proteins found on LDs, revealing a wide range of enzymes, structural proteins, and motor proteins, as well as numerous other proteins with hitherto unclear roles in LD biology (Welte, 2015; Thiam and Beller, 2017; Huang, 2018; Zhang and Liu, 2019). Research in the last decade has revealed also that LDs are not simply static oil depots, but rather dynamic organelles involved in a multitude of intracellular processes (Gao and Goodman, 2015). In plants, for instance, LDs are involved in post-germinative seedling growth, abiotic stress responses (Gidda et al., 2016; Kim et al., 2016), stomatal opening/closing (McLachlan et al., 2016), cuticular lipid formation (Zhang et al., 2016), synthesis of antifungal compounds (Shimada and Hara-Nishimura, 2015), and pollen tube growth (Müller and Ischebeck, 2018).

Despite their physiological importance, the biogenesis of LDs in plants is not well understood. Nonetheless, a general model has emerged wherein neutral lipids, such as TAGs, are first synthesized by membrane-associated enzymes at the ER. The neutral lipids then accumulate between the leaflets of the ER membrane as a lipid “lens” that subsequently emerges on the cytoplasmic side of the ER as a nascent LD (Olzmann and Carvalho, 2019; Jackson, 2019; Henne et al., 2020; Thiam and Ikonen, 2021). While this process can be induced with synthetic, emulsified LD systems in vitro by incorporating phospholipids that reduce the surface tension on one side of the membrane bilayer (Chorlay et al., 2019), LD formation at the ER in vivo appears to be a highly orchestrated, stepwise process that involves numerous proteins.

In plants, most studies related to LD biogenesis have been performed on oilseeds, where the well-known oleosins are the major LD coat proteins in seeds, which are thought to partition into the lipid lens in the ER membrane and promote emergence of the nascent LD from the ER (Huang, 2018). The loss of oleosin proteins in plants results in the formation of fewer, larger LDs in seeds, likely due to LD–LD fusion (Siloto et al., 2006; Schmidt and Herman, 2008; Miquel et al., 2014). Thus, oleosin appears to be critically important for stabilizing LDs, particularly during seed desiccation and rehydration. However, oleosins are predominantly expressed in seed tissues and consequently, far less is known about LD formation in other types of plant cells. Despite the limited understanding of LD biogenesis in plants, important advances have come primarily from recent studies of two groups of proteins that are constitutively expressed, namely the SEIPINs (Cai et al., 2015; Taurino et al., 2018), which are evolutionarily conserved in eukaryotes, and the LD-ASSOCIATED PROTEINS (LDAPs; Gidda et al., 2013, 2016; Horn et al., 2013; Kim et al., 2016), which are considered unique to plants (Pyc et al., 2017a; Chapman et al., 2019).

SEIPIN was first identified in humans where mutations in its gene sequence were associated with a neutral lipid storage disorder called Berardinelli–Seip congenital lipodystrophy. The loss of SEIPIN function resulted in a near absence of LDs in most tissues (Garg and Agarwal, 2009). Subsequent characterization of the protein in mammals, yeast, and insects revealed that SEIPIN is an integral ER membrane protein that forms a large, toroidal oligomeric complex composed of 10 to 12 subunits that is involved in the earliest stages of LD formation (Binns et al., 2010; Sui et al., 2018; Yan et al., 2018; Salo et al., 2019; Prasanna et al., 2021; Zoni et al., 2021). Specifically, the SEIPIN complex is critically important for LD initiation, where it “traps” neutral lipids in the ER bilayer and acts as a “vent” for the transfer of lipids from the ER into the expanding LD (Binns et al., 2010; Cartwright and Goodman, 2012; Salo et al., 2020). SEIPIN in animal cells is also known to interact with several other proteins to help coordinate the process of LD formation (Chen and Goodman, 2017; Bohnert, 2020; Salo et al., 2020). Notable among these proteins is promethin (more recently renamed Lipid Droplet Assembly Factor 1 [LDAF1]), which interacts with the SEIPIN complex to facilitate LD formation at the ER and then dissociates from SEIPIN to become localized on the expanding, nascent LD surface (Castro et al., 2019; Chung et al., 2019; Chartschenko et al., 2020; Prasanna et al., 2021). Unlike in animals and yeast, which have single copies of the *SEIPIN* gene, Arabidopsis (*Arabidopsis thaliana*) has three *SEIPIN* genes: *SEIPIN1–3* (Cai et al., 2015). The heterologous expression of Arabidopsis *SEIPINs* in plant (i.e. *Nicotiana benthamiana*) leaves greatly increases the number of LDs, and *SEIPIN1* favors the formation of a greater proportion of larger LDs, while *SEIPIN2* and *SEIPIN3* produce more normal-sized and smaller LDs, respectively (Cai et al., 2015). Similarly, in Arabidopsis seeds, the disruption of two or all three of the *SEIPIN* genes results in the formation of enlarged LDs that were, on occasion, not localized in the cytoplasm, but rather accumulated in the nucleus (Taurino et al., 2018). While it is known that the plant SEIPIN isoforms influence LD size and number based, at least in part, on their interaction with the membrane-tethering protein VESICLE-ASSOCIATED MEMBRANE PROTEIN-ASSOCIATED PROTEIN 27-1 (Greer et al., 2020) at ER–LD junctions, it remains to be determined whether other proteins work together with plant SEIPINs to influence LD formation.

The LDAPs were initially identified as plant-specific LD coat proteins based on proteomic analysis of LDs purified from avocado (*Persea americana*) mesocarp (Horn et al., 2013), which is an oil-rich tissue that lacks an abundance of oleosins. Homologs of LDAPs were subsequently found in proteomes of LDs in various other plant species, including Arabidopsis (Brocard et al., 2017; Zhi et al., 2017; Kretzschmar et al., 2020; Sturtevant et al., 2020). LDAPs also share extensive sequence similarity with the small rubber particle proteins in rubber-accumulating plants (Berthelot et al., 2014), suggesting that rubber particles and TAG-containing LDs are similar organelles that package different types of neutral lipids (Gidda et al., 2013; Horn et al., 2013). LDAPs are broadly conserved in plants and there are three LDAP genes (*LDAP1–3*) in Arabidopsis, each of which is constitutively expressed (Gidda et al., 2016; Kim et al., 2016). However, the Arabidopsis LDAPs are also selectively induced during abiotic stress conditions or enriched on LDs at certain developmental stages, such as senescence (Brocard et al., 2017). LDs are known to proliferate during abiotic stress responses in plants, and the ectopic overexpression of LDAPs can increase LD abundance in leaves and improve the tolerance of plants to drought (Gidda et al., 2016; Kim et al., 2016; Laibach et al., 2018). Conversely, disruption of any of the LDAPs in plants decreases the number of LDs in leaves (Gidda et al., 2016; Kim et al., 2016). Thus, LDAPs appear to be important for modulating the number of LDs in plants and may have distinct functions under certain physiological conditions.

To gain insight into how LDAPs function in plant cells, we previously conducted a yeast two-hybrid (Y2H) screen using Arabidopsis LDAP3 as bait and identified a largely hydrophobic protein of unknown function that we called LDAP-INTERACTING PROTEIN (LDIP) (Pyc et al. 2017b). Subsequent characterization of LDIP revealed that it localized to LDs and interacted with LDAPs on the LD surface (Brocard et al., 2017; Pyc et al., 2017b; Kretzschmar et al., 2018; Coulon et al., 2020). LDIP has a hydrophilic N-terminal region that is both necessary and sufficient for LD localization, and the loss of LDIP function in Arabidopsis results in the formation of fewer, but larger LDs (Pyc et al., 2017b). Collectively, these and other observations suggested that LDIP might bind first to LDs, where it serves as an anchor to recruit LDAPs for proper compartmentation of LDs (Chapman et al., 2019). Proteomic analysis of avocado mesocarp, however, revealed that LDIP was enriched in the microsomal fraction (Horn et al., 2013) rather than LDs, suggesting that LDIP might also function at the ER. Moreover, affinity-capture experiments using LDIP as bait revealed that LDIP interacted not only with LDAPs, but also with several other LD-related proteins, including ER-localized SEIPINs (Pyc et al., 2017b). Thus, LDIP appeared to associate with both ER-localized LD biogenetic proteins (i.e. SEIPIN) as well as established LD coat proteins (LDAPs). Here, we characterized the functional interactions of LDIP, LDAP, and SEIPIN, and show that LDIP plays a key role in LD biogenetic processes that involve both ER-localized SEIPINs, as well as LD-localized LDAPs. Notably, LDIP shows distant homology with human LDAF1, and our studies reveal structural and functional similarities between SEIPIN/LDIP relationships in plants and SEIPIN/LDAF1 in animals. These findings allowed us to draw parallels to models of LD biogenesis in mammals and propose a new, more generalized model of LD biogenesis in plants that involves a protein complex at the ER and coat proteins on the LD surface.

## Results

### LDIP is not required for the localization of LDAPs to LDs

LDIP contains a discrete LD targeting signal and a substantial hydrophobic domain (Pyc et al., 2017b), while LDAPs are generally hydrophilic proteins that require the full-length protein sequence for LD targeting (Gidda et al., 2016). Consequently, we initially asked whether LDIP might serve as an anchor that binds first to LDs, then recruits LDAP to the LD surface via protein–protein interaction. If LDIP is required for LDAP localization to LDs, then a loss of LDIP should result in the mislocalization of LDAPs to the cytoplasm. This premise was tested in two different ways: (1) the ectopic expression of Cherry-fluorescent-protein-tagged LDAPs in *ldip*-knockout (*KO*) plants and (2) a comparative analysis of LD proteomes derived from the wild-type (*WT*) and *ldip* mutant seedlings.

**Figure 1:**
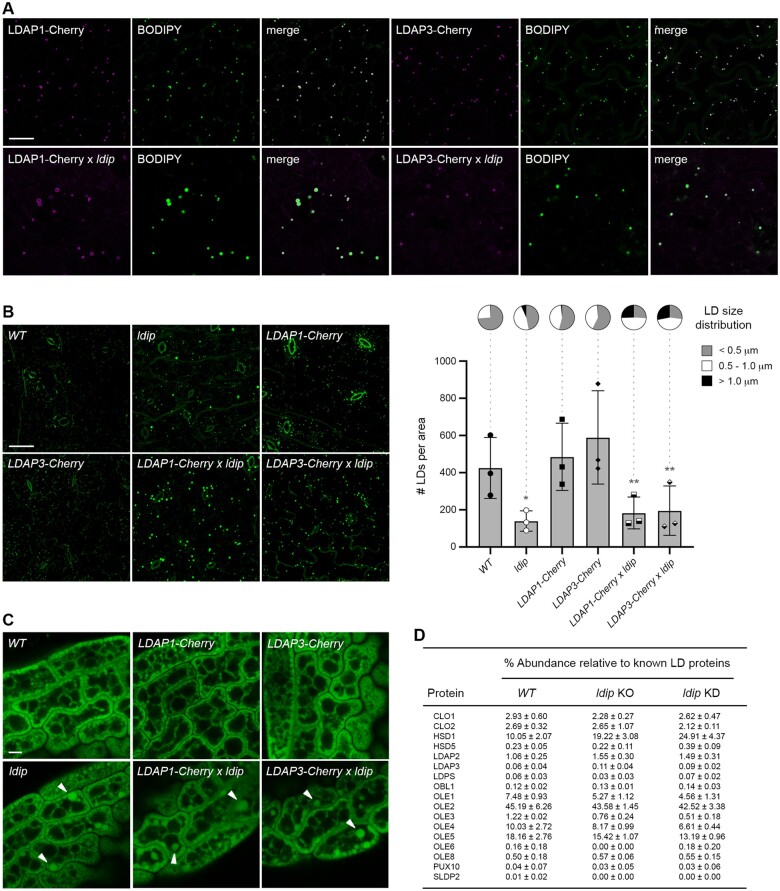
LDIP is not required for the localization of LDAPs to LDs but is important for regulating LD numbers and sizes independent of LDAPs. A, Representative CLSM images (z-sections) of LDAP1-Cherry and LDAP3-Cherry localization in leaf epidermal cells of 15-day-old stable transgenic Arabidopsis seedlings from *LDAP1/3-Cherry* parental lines or crossed *LDAP1/3-Cherry* x *ldip* (*KO*) lines, as indicated by labels. Shown also are images of the LDs in the same cells, stained with BODIPY, as well as the corresponding merged images. Quantifications of LDAP1/3-Cherry and BODIPY colocalizations in images (*n* = 7-8) from each of the four plant lines, based on the mean ± standard deviation (sd) of Manders’ co-occurrence coefficient, were as follows: *LDAP1-Cherry* = 0.865±0.063; *LDAP3-Cherry* 0.835±0.096; *LDAP1-Cherry* × *ldip* = 0.922±0.026; *LDAP3-Cherry* × *ldip* = 0.841±0.087. RT-PCR confirmation of transgene (i.e. *LDAP1/3-Cherry*) expression and/or disruption of endogenous *LDIP* expression in various lines are shown in Supplemental Figure S2A. Bar = 20 µm and applies to all images in the panel. B, LD numbers and sizes in leaves of Arabidopsis *WT*, *ldip KO*, *LDAP1/3-Cherry*, and *LDAP1/3-Cherry* × *ldip* plant lines. Shown on the left are representative CLSM images (z-stacks) of the BODIPY-stained LDs in leaf epidermal and mesophyll cells of 15-day-old seedlings from each line, as indicated by labels. Bar = 20 µm and applies to all images in the panel. Quantifications of LD numbers per area and LD sizes are shown in the graphs on the right. Values of LD numbers are the mean ± sd from three biological replicates, with each replicate consisting of eight leaf samples per line and two micrographs per leaf sample. LD diameters were calculated using the same data set (i.e. micrographs) and are presented as the distribution of LDs in three size classes: <0.5 µm (small), 0.5–1.0 µm (intermediate), and >1.0 µm (large); refer also to key. Single and double asterisks in graph represent statistically significant differences at *P* ≤0.05 and *P* ≤0.01 relative to the *WT* and *LDAP1/3-Cherry* lines, respectively, as determined by an Analysis of Variance (ANOVA) test followed by a Dunnett post hoc multiple comparisons test. A summary of the statistical analysis is shown in Supplemental Data Set 1. Refer also to Supplemental Figure S1A for violin plots representing the average LD sizes (i.e. LD diameters) in the same lines, based on the data set used here in (B). C, Representative CLSM images (z-sections) of BODIPY-stained LDs in mature, dry seeds of Arabidopsis *WT*, *ldip KO*, *LDAP1/3-Cherry*, and *LDAP1/3-Cherry* × *ldip* plant lines, as indicated by labels. Arrowheads highlight examples of larger LDs in *ldip* and *LDAP1/3-Cherry* × *ldip* seeds. Bar = 5 µm and applies to all images in the panel. D, Relative abundance of LDAPs and other known LD proteins in LD proteomes derived from the *WT* and *ldip* mutant seedlings. LD-enriched fractions were isolated from 40-h-old germinated seedlings of the *WT* and *ldip KO* or *KD* mutant lines. Proteins from three biological replicates (i.e. three separate LD isolations per line) were in-gel digested with trypsin and analyzed by liquid chromatography-tandem mass spectrometry. Protein levels were calculated using the label-free quantification algorithm (Cox and Mann, 2008; Cox et al., 2014); see Supplemental Data Sets 2–4 for the values and enrichment ratios for all proteins identified in all samples. All of the proteomics data are available also through the ProteomeXchange Consortium via the PRIDE partner repository (Accession No. PXD012992); refer to Supplemental Table S1. Protein abundances shown are the mean ± sd from the three biological replicates and were normalized to the percentage of known Arabidopsis LD proteins (based on Kretzschmar et al., 2020) in each sample. CLO, caleosin; HSD, hydroxysteroid dehydrogenase (steroleosin); LDPS, LD protein of seeds; OBL, oil body lipase; OLE, oleosin; PUX, plant UBX-domain-containing protein, SLDP, seed LD protein.

To generate Arabidopsis lines expressing Cherry-tagged LDAPs in the *ldip* mutant background, homozygous *ldip KO* plants (Pyc et al., 2017b) were crossed with homozygous plants ectopically expressing either LDAP1-Cherry or LDAP3-Cherry (Gidda et al., 2016), and then heterozygous progeny were advanced to homozygosity. As shown in Figure 1A, confocal microscopy analysis of leaf epidermal cells from *LDAP1-Cherry* and *LDAP3*-*Cherry* parental lines showed that both proteins targeted to LDs stained with the neutral lipid-specific dye boron-dipyrromethene (BODIPY) 493/503 (Listenberger et al., 2007), as previously reported (Gidda et al., 2016). Similarly, both LDAP1-Cherry and LDAP3-Cherry localized to LDs in leaves in the *ldip KO* background (Figure 1A), indicating that LDIP is not required for the localization of LDAPs to LDs. Further, there appeared to be fewer and larger LDs in leaves of the *LDAP1/3-Cherry* × *ldip* mutant lines compared to the *LDAP1/3-Cherry* parental lines (Figure 1A). Quantification of the numbers and sizes of LDs in leaves from the various parental and progeny plant lines revealed that, in comparison to the *WT*, the loss of LDIP resulted in a significant decrease in the LD number, but an increase in the average LD size, including the proportion of intermediate- and large-sized LDs with diameters of 0.5–1 µm and >1.0 µm, respectively (Figure 1B; refer also to Supplemental Figure S1A for the analysis of average LD size), consistent with previous results (Pyc et al., 2017b). Comparatively, the expression of either LDAP1-Cherry or LDAP3-Cherry in a *WT* background resulted in a slight increase in the number of LDs relative to the *WT*, and a concomitant increase in the average LD size, including the proportion of intermediate-sized LD (Figure 1B;  Supplemental Figure S1A), also as expected (Gidda et al., 2016). The expression of LDAPs in the *ldip KO* background (i.e. *LDAP1/3-Cherry* × *ldip*), however, resulted in a phenotype more similar to the *ldip KO*, with significantly fewer, but larger LDs relative to the *LDAP1/3-Cherry* parental lines (Figure 1B;  Supplemental Figure S1A). In fact, the ectopic expression of LDAPs further increased the proportion of larger sized (i.e. >1.0 µm diameter) LDs in comparison to the *ldip KO* background alone (Figure 1B;  Supplemental Data Set S1; refer also to Supplemental Figure S2A for reverse transcription–polymerase chain reaction [RT-PCR] confirmation of transgene [i.e. *LDAP1/3-Cherry*] expression and/or disruption of endogenous *LDIP* expression in the abovementioned lines).

As in leaves, enlarged LDs also were observed in mature (dry) seeds of *LDAP1/3-Cherry* × *ldip* plants (Figure 1C). LDAPs are minor constituents of the LD protein coat in seeds (Kretzschmar et al., 2020), and proteomic analysis showed that LDAP2 and LDAP3 (LDAP1 was undetectable at this stage of development in this study, but present in others [Kretzschmar et al., 2020]) were still associated with LDs in *ldip KO* or *ldip* knockdown (*KD*) 40-h-old, germinated seedlings (Figure 1D;  Supplemental Data Sets S2–S4). Other known LD proteins, including oleosins and caleosins, also were associated with LDs in *ldip* mutant seedlings (Figure 1D). Notably, the relative abundance of several LD proteins either increased, as for LDAP2 and hydroxysteroid dehydrogenase 1, or decreased, as for oleosins, in *ldip* mutant seedlings relative to the *WT*. Whether these changes are a direct or indirect result of LDIP disruption remains to be determined.

Taken together, the results presented in Figure 1 indicate that: (1) LDIP is not required for the association of LDAPs and most other LD proteins with LDs in either leaves or seedlings and (2) LDIP is critically important for regulating LD numbers and sizes in a manner that appears to be independent of and perhaps upstream of the function of LDAPs and oleosins in LD compartmentation.

### LDIP is recruited to the LD surface by LDAP3 when co-expressed in insect cells

**Figure 2:**
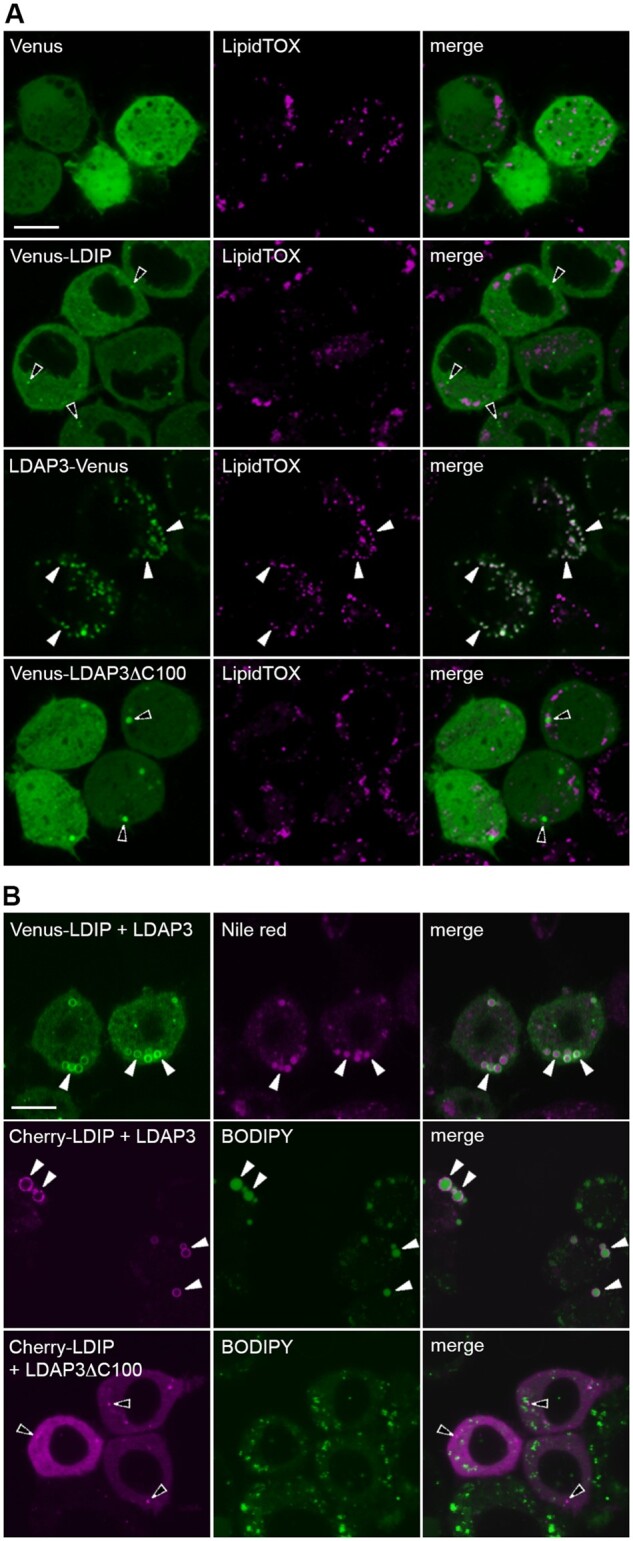
LDAP targets to LDs in insect cells and recruits LDIP to the LD surface. Representative CLSM images (z-sections) of insect (*T. ni*)-cultured cells either (A) transiently transformed with either Venus alone or Venus-tagged Arabidopsis LDIP or LDAP3 or a C-terminal 100-amino-acid-truncated version of LDAP3 (LDAP3ΔC100) or (B) stably transformed with nontagged LDAP3 or LDAP3ΔC100 and transiently-transformed with *Venus-* or Cherry-tagged LDIP, as indicated by labels. All cells were grown in oleate-containing media to stimulate LD growth and proliferation (Thiel et al., 2013) and LDs were stained with LipidTOX, Nile red, or BODIPY. Shown also are the corresponding merged images. Open arrowheads indicate examples of the non-colocalization of an expressed fusion protein and LDs; solid white arrowheads indicate examples of colocalization of an expressed fusion protein and LDs. RT-PCR analysis confirming the expression of *LDAP3* or *LDAP3ΔC100* in stably transformed cell lines in (B) are presented in Supplemental Figure S2B. Bars in (A) and (B) = 10 µm and applies to all images in the panels.

Given that LDIP and LDAP were previously shown to physically interact on the LD surface (Pyc et al., 2017b), and LDIP is not required for LDAP association with LDs (Figure 1), we next asked if instead LDAPs might be important for localizing LDIP to LDs. This was tested by expressing Arabidopsis LDAP3 and LDIP individually or in combination in insect (*Trichoplusia ni*) cultured cells. We selected this system since potential homologs of LDIP and LDAP in insects, like other metazoans, are only distantly related (see below), which reduces the possibility of interactions between ectopically expressed plant proteins and endogenous insect proteins. As shown in Figure 2A, the transient expression of Venus-fluorescent-protein-tagged LDIP (Venus-LDIP) in insect cells resulted in its localization predominantly to the cytoplasm, similar to the localization of Venus alone. While some punctate fluorescence was observed in Venus-LDIP-expressing cells (refer to open arrowheads in Figure 2A), these structures did not co-localize with LDs stained with the neutral lipid-specific dye HCS LipidTOX Deep Red. In contrast, transiently expressed LDAP3-Venus readily localized to LipidTOX-stained LDs (Figure 2A), supporting the results from plant cells (Figure 1A) that LDIP is not required for targeting of LDAP3 to LDs. On the other hand, a truncated version of LDAP3 lacking its C-terminal 100 amino acids (Venus-LDAP3ΔC100), which disrupts the ability of the protein to target to LDs in plant cells (Gidda et al., 2016), did not target to LDs in insect cells (Figure 2A), suggesting that the LD targeting pathway for LDAP3 is similar in insect and plant cells.

We tested next whether LD-localized LDAP3 could recruit LDIP to LDs by generating stable insect cell lines that expressed either nontagged, full-length LDAP3 or truncated LDAP3ΔC100. As shown in Figure 2B, both Venus- or Cherry-tagged LDIP transiently expressed in cells stably expressing full-length LDAP3 localized to LDs stained with Nile red, a neutral lipid-specific dye (Greenspan et al., 1985), or BODIPY, respectively, although the LDs in these cells were relatively large compared to LDs in cells expressing LDIP or LDAP3 individually (compare with images in Figure 2A). On the other hand, transiently expressed Cherry-LDIP localized to the cytoplasm and punctate structures, but not to LDs, in cells stably expressing the truncated LDAP3ΔC100 protein (Figure 2B), similar to when Venus-LDIP was expressed on its own (Figure 2A). Whether the apparent increase in LD size in insect cells co-expressing LDIP and LDAP3 (Figure 2B) reflects a biologically relevant function of these proteins in plant cells, or an artifact of the heterologous expression system, remains to be determined. Nonetheless, these results are consistent with those in Arabidopsis leaves and seeds showing that LDAPs can target to LDs independently of LDIP (Figure 1) and, further, that LDAPs are important for localizing LDIP to the LD surface.

### LDIP works together with LDAPs and oleosins to form normal-sized LDs in plant cells

**Figure 3:**
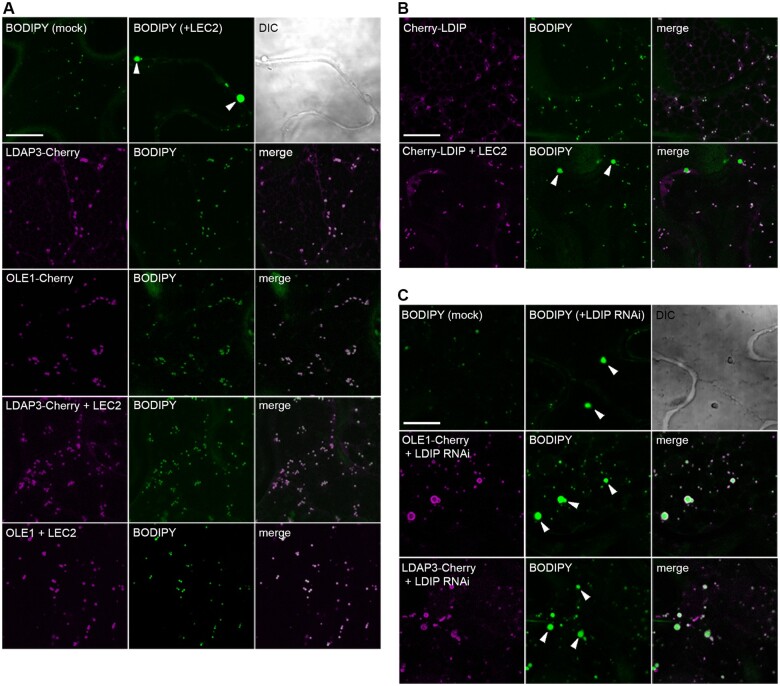
LDIP, unlike LDAP3 or oleosin, is unable to compartmentalize neutral lipids into normal-sized LDs in plant cells. (A)–(C) Representative CLSM images (z-sections) of *N. benthamiana* leaf epidermal cells transiently (co)transformed with either Cherry-tagged LDAP3, LDIP, or OLE1, along with or without Arabidopsis LEC2 or an RNAi for endogenous *N. benthamiana LDIP*, or with LEC2 alone or mock transformed, as indicated by labels. All cells in (A) and (B) were also (co)transformed with P19, serving as a suppressor of gene silencing (Petrie et al., 2010). LDs in all cells were stained with BODIPY. Shown also in the top rows in (A) and (C) are the corresponding differential interference contrast images of the cells transformed with LEC2 or *LDIP* RNAi, respectively. Note the presence of aberrant, supersized LDs (indicated with arrowheads) in cells transformed with either LEC2 alone (top row in (A)), Cherry-LDIP and LEC2 (bottom row in (B)), or with *LDIP* RNAi, either alone or with OLE1-Cherry or LDAP3-Cherry (C). In contrast, all mock-transformed cells or those expressing LDAP3-Cherry or OLE1-Cherry with or without LEC2 possess normal-sized LDs. Bars in (A)–(C) = 20 µm and applies to all images in the panel. RT-PCR analysis confirming transgene expression in samples in (A) and (B) (and in Supplemental Figure S3) is presented in Supplemental Figure S2C; RT-PCR and RT-qPCR analysis confirming the suppression of endogenous *LDIP* expression in (C) are presented in Supplemental Figure S2, D and E, respectively.

The presence of enlarged LDs in both leaves and seeds of *ldip* mutant plants (Figure 1, B and C; Pyc et al., 2017b) was somewhat surprising given that the predominant LD coat proteins (i.e. LDAPs and oleosins) were still associated with LDs in both organ types (Figure 1D). These observations suggested that LDIP, akin to LDAPs and oleosin, might be serving, at least in part, as an important structural LD coat protein to help determine proper LD size. To investigate this possibility, we used a transient expression system wherein the Arabidopsis LEAFY COTYLEDON 2 (LEC2) transcription factor was ectopically expressed in *N. benthamiana* leaves. LEC2 is normally expressed in developing seeds, where it upregulates multiple genes associated primarily with fatty acid biosynthesis. The ectopic expression of LEC2 in leaves also upregulates genes for fatty acid biosynthesis (Santos Mendoza et al., 2005; Vanhercke et al., 2017), but genes for LD structural coat proteins, such as oleosins, are not as strongly upregulated (Feeney et al., 2013; Kim et al., 2013). This deficiency in LD coat proteins leads to the formation of aberrant, supersized LDs that are not observed in mock-transformed leaf cells (refer to arrowheads in Figure 3A), as previously shown (Gidda et al., 2016). The co-expression of either LDAP3-Cherry or the Cherry-tagged Arabidopsis oleosin isoform 1 (Cherry-OLE1) with LEC2, however, increases the availability of coat proteins and suppresses the formation of supersized LDs, resulting in more normal-sized LDs (Figure 3A), also as previously shown (Gidda et al., 2016).

To test whether LDIP also has the ability to compartmentalize the enlarged LDs in LEC2-expressing leaves, we first expressed Cherry-LDIP on its own and showed that the protein targets to LDs in *N. benthamiana* leaves, as expected (Figure 3B; Brocard et al., 2017; Pyc et al., 2017b; Coulon et al., 2020). The co-expression of Cherry-LDIP and LEC2, however, did not suppress the presence of supersized LDs (Figure 3B). Similar results were observed when LDIP was co-expressed with a mutant version of the mouse (*Mus musculus*) fat storage-inducing transmembrane protein 2 (FIT2^Mut^), which also produces aberrant, supersized LDs when ectopically expressed in plant cells (Cai et al., 2017). That is, the supersized LDs observed in FIT2^Mut^-transformed *N. benthamiana* leaf cells were not suppressed by co-expression with Cherry-LDIP but were suppressed by co-expression with LDAP3-Cherry (Supplemental Figure S3).

Given that both LDAP and oleosin can suppress the formation of supersized LDs in LEC2-expressing leaves (Figure 3A), while supersized LDs remain in *LDAP1/3-Cherry* × *ldip* and *ldip* mutant plants (Figure 1), we asked next whether LDIP is required for the formation of normal-sized LDs in combination with LDAP or oleosin proteins. To test this possibility, we developed an RNA interference (RNAi)-based assay to suppress the endogenous *LDIP* expression in *N. benthamiana* leaves. As shown in Figure 3C, *LDIP* RNAi-transformed *N. benthamiana* leaf cells, similar to *ldip* mutant Arabidopsis leaves (Figure 1B; Pyc et al., 2017b), possessed several conspicuously enlarged BODIPY-stained LDs which were not observed in mock-transformed cells. As shown also in Figure 3C, the co-expression of either LDAP3-Cherry or OLE1-Cherry with *LDIP* RNAi did not supress the appearance of the supersized LDs. In fact, both LDAP3-Cherry and OLE1-Cherry were localized to the periphery of the supersized LDs, as well as to the periphery of the other, more normal-sized LDs in these cells (Figure 3C). Taken together these data and the other results presented in Figure 2 point to several conclusions: (1) LDIP is not sufficient for the production of normal-sized LDs when oleosins or LDAPs are limiting (i.e. when LDIP is co-expressed with LEC2 in leaves); (2) LDAPs and oleosins also are not sufficient for the formation of normal-sized LDs when LDIP is limiting (i.e. when OLE1 or LDAP are co-expressed with an *LDIP* RNAi); and (3) LDIP and LDAPs/oleosins participate together to produce normal-sized LDs in plant cells.

### LDIP interacts with ER-localized SEIPIN

**Figure 4:**
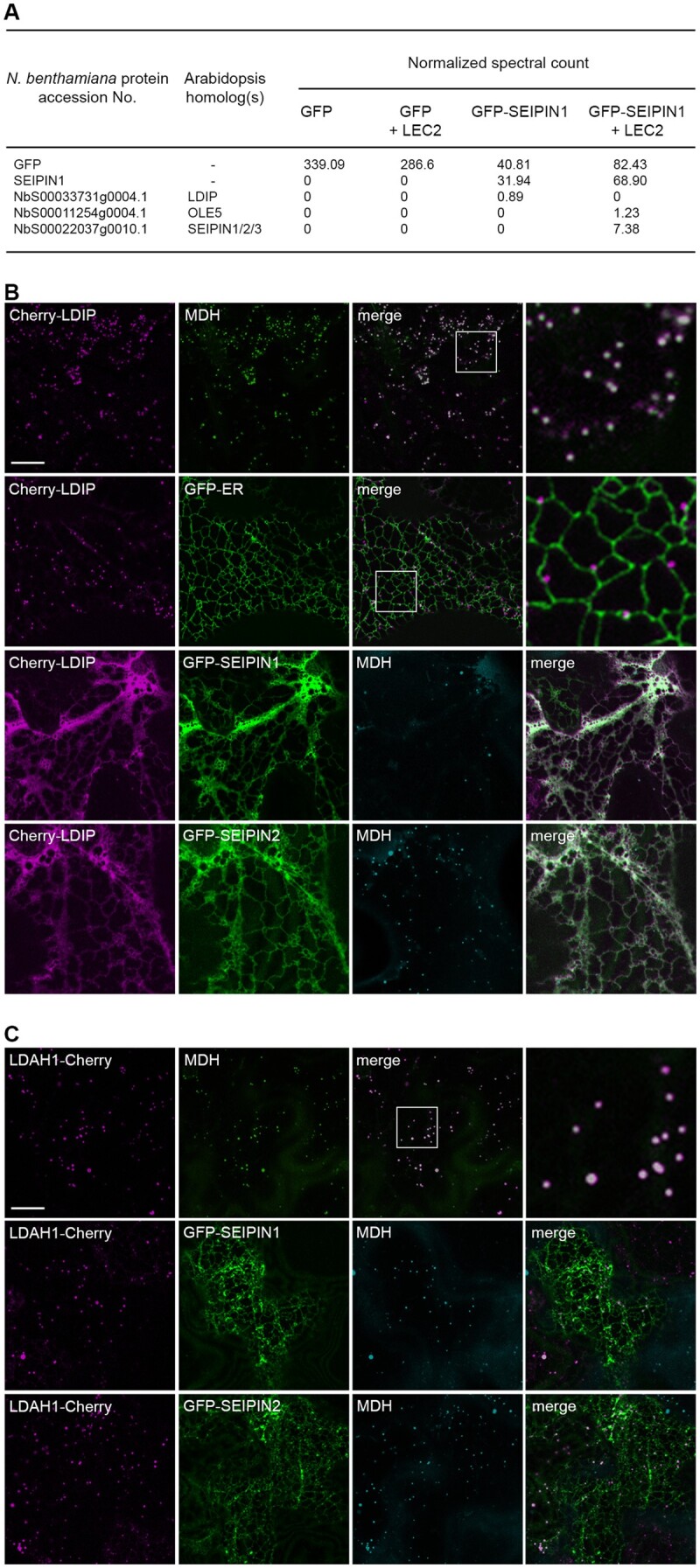
Interaction of LDIP and SEIPIN in plant cells. A, Affinity capture of SEIPIN1 transiently expressed in *N. benthamiana* leaves. Listed are selected MS-identified *N. benthamiana* LD or LD-related proteins (based on those described in Ischebeck et al. (2020)) that co-immunoprecipitated with expressed GFP or GFP-SEIPIN1, with or without co-expressed LEC2. Proteins were captured using agarose-conjugated anti-GFP antibodies. The accession numbers and descriptions of *N. benthamiana* proteins were acquired from the *N. benthamiana* genome database (i.e. SGN), and Arabidopsis Gene Identifier numbers and protein names of the closest Arabidopsis homologs were obtained from TAIR. Spectral counts of each protein were normalized to the average of the sums of all MS samples in the experiment. All *N. benthamiana* proteins identified in pull-downs of GFP-SEIPIN1 with or without LEC2, as well as pull-downs of GFP-LDIP with and without LEC2 reported in Pyc et al. (2017b), are shown in Supplemental Data Set S5. All of the proteomics data are available also through the ProteomeXchange Consortium via the PRIDE partner repository (Accession No. PXD023043); refer to Supplemental Table S2. B, C, Representative CLSM images (*z*-sections) of *N. benthamiana* leaf epidermal cells (co)transformed, as indicated by labels, with either Cherry-LDIP or LDAH1-Cherry alone, or with either GFP-ER, serving as an ER marker protein, or with GFP-SEIPIN1 or GFP-SEIPIN2. LDs were stained with MDH. Shown also are the corresponding merged images. The boxes in some merged images represent the portion of the cells shown at higher magnification in the panels to the right. Bars in (B) and (C) = 20 µm and applies to all images in the panels, with the exception of those showing a portion of a cell at higher magnification.

Given that the loss of LDIP in plants results in fewer and larger LDs (Figure 1; Pyc et al., 2017b; Coulon et al., 2020), we asked next whether LDIP might work together with other LD biogenetic proteins, such as SEIPIN, to modulate the numbers and sizes of LDs in plants. SEIPIN is known to be critically important for LD formation and altering expression levels through gene-knockouts or ectopic overexpression significantly influences the number and size of LDs in plants (Cai et al., 2015; Taurino et al., 2018) and other eukaryotes (Szymanski et al., 2007; Fei et al., 2008; Wang et al., 2016). Previous studies involving the expression of green fluorescent protein (GFP)-tagged LDIP in *N. benthamiana* leaves, followed by affinity-capture with GFP antibodies and proteomic analysis, showed that LDIP associated not only with itself and LDAPs, but also with SEIPINs (Pyc et al., 2017b). Moreover, both SEIPINs and oleosins were identified in affinity-capture experiments when GFP-LDIP was co-expressed with LEC2 (Pyc et al., 2017b), which, as mentioned previously, increases storage lipid biosynthesis and also, although to a lower extent, induces LD coat proteins (Santos Mendoza et al., 2005; Feeney et al., 2013; Kim et al., 2013; Vanhercke et al., 2017). To confirm and extend these observations, we performed similar affinity-capture experiments in *N. benthamiana* leaves using GFP-tagged Arabidopsis SEIPIN1 as bait, with and without co-expressed LEC2. As shown in Figure 4A, affinity-capture with GFP-SEIPIN1 resulted in the recovery of endogenous LDIP, while GFP-SEIPIN1 and LEC2 resulted in the capture of endogenous oleosin (i.e. oleosin isoform 5 [OLE5]) and SEIPINs. These results support the premise that LDIP is in close proximity to and possibly interacts with SEIPIN proteins in plant cells.

The association of LDIP and SEIPIN initially was somewhat unexpected, given that LDIP is predominantly localized to LDs, while SEIPIN is an ER-localized membrane protein (Cai et al., 2015; Brocard et al., 2017; Müller et al., 2017; Pyc et al., 2017b; Taurino et al., 2018; Coulon et al., 2020; Greer et al., 2020). To further investigate the potential relationship between LDIP and SEIPIN proteins, we first considered the proximal association of LDs and ER in plant cells. Prior studies revealed that LDs are often closely associated with the ER in plant cells (Cai et al., 2015; Brocard et al., 2017; Greer et al., 2020), and in some organisms, such as yeasts, LDs remain physically attached to the ER and can expand or shrink depending on the physiological needs of the cell (Hugenroth and Bohnert, 2020). As shown in Figure 4B, the expression of Cherry-LDIP in *N. benthamiana* leaves confirmed the steady-state localization of the protein primarily to LDs stained with the neutral lipid-specific dye monodansylpentane (MDH; Yang et al., 2012b). The co-expression of Cherry-LDIP with a GFP-tagged ER marker protein (i.e. GFP-ER) further revealed that the majority of the LDIP-containing LDs were indeed closely associated with the ER (Figure 4B). These results support the premise that a portion of LDIP and SEIPIN proteins might be in close proximity at ER–LD junction sites. The co-expression of Cherry-LDIP with GFP-tagged Arabidopsis SEIPIN1 or SEIPIN2 (GFP-SEIPIN1/2), however, resulted in a dramatic alteration in the subcellular distribution of LDIP, whereby Cherry-LDIP was co-localized with GFP-SEIPIN1 or GFP-SEIPIN2 throughout the ER network, instead of at MDH-stained LDs (Figure 4B). Indeed, the co-localization of Cherry-LDIP and GFP-SEIPIN1 or GFP-SEIPIN2 was even more pronounced at extended time periods (Supplemental Figure S4), when the overexpression of SEIPIN is known to reorganize the ER in plant cells (Cai et al. 2015; Taurino et al., 2018; Greer et al., 2020). Co-localization at the ER was not observed, however, when a different LD protein, LD-ASSOCIATED HYDROLYASE 1 (LDAH1; Kretzschmar et al., 2020), was co-expressed with SEIPIN1/2. As shown in Figure 4C, the expression of LDAH1-Cherry alone in *N. benthamiana* leaves resulted in localization of the protein to MDH-stained LDs, as expected (Kretzschmar et al., 2020). But, unlike Cherry-LDIP, the localization of LDAH1-Cherry to LDs was unaffected by co-expression with GFP-SEIPIN1 or GFP-SEIPIN2 (Figure 4C). Taken together, these observations suggest that the localization of LDIP to LDs is dynamic in nature, and the increase in steady-state amount of SEIPIN protein upon co-expression results in localization of LDIP to the ER, possibly due to protein–protein interactions.

The alteration in subcellular distribution of LDIP upon co-expression with SEIPIN1 or SEIPIN2 (Figure 4B) was reminiscent of results observed when human SEIPIN was ectopically (co)expressed with LDAF1, i.e. LDAF1 is localized to LDs in mammalian cells under steady-state conditions, but co-expression with *SEIPIN* results in its re-localization to the ER (Castro et al., 2019). Structural analysis of the human SEIPIN-LDAF1 complex by cryo-electron microscopy (EM) revealed that LDAF1 physically interacts with SEIPIN in a 1:1 stoichiometric manner by binding to a hydrophobic helix (HH) present in SEIPIN (Chung et al., 2019). Notably, the HH sequence in human SEIPIN is well conserved among homologs in other species (Chung et al., 2019), including plants, and structural homology modeling studies indicate that plant SEIPIN proteins can adopt a similar 3D structure as their human (*Homo sapiens*) and fly (*Drosophila melanogaster*) counterparts (Chapman et al., 2019; refer also to Supplemental Figure S5). Plant LDIPs also share sequence homology with human LDAF1, albeit remotely, and both proteins possess similar hydropathy profiles (Supplemental Figure S6, A and B), suggesting they might perform analogous functions in spite of their limited sequence similarity.

**Figure 5:**
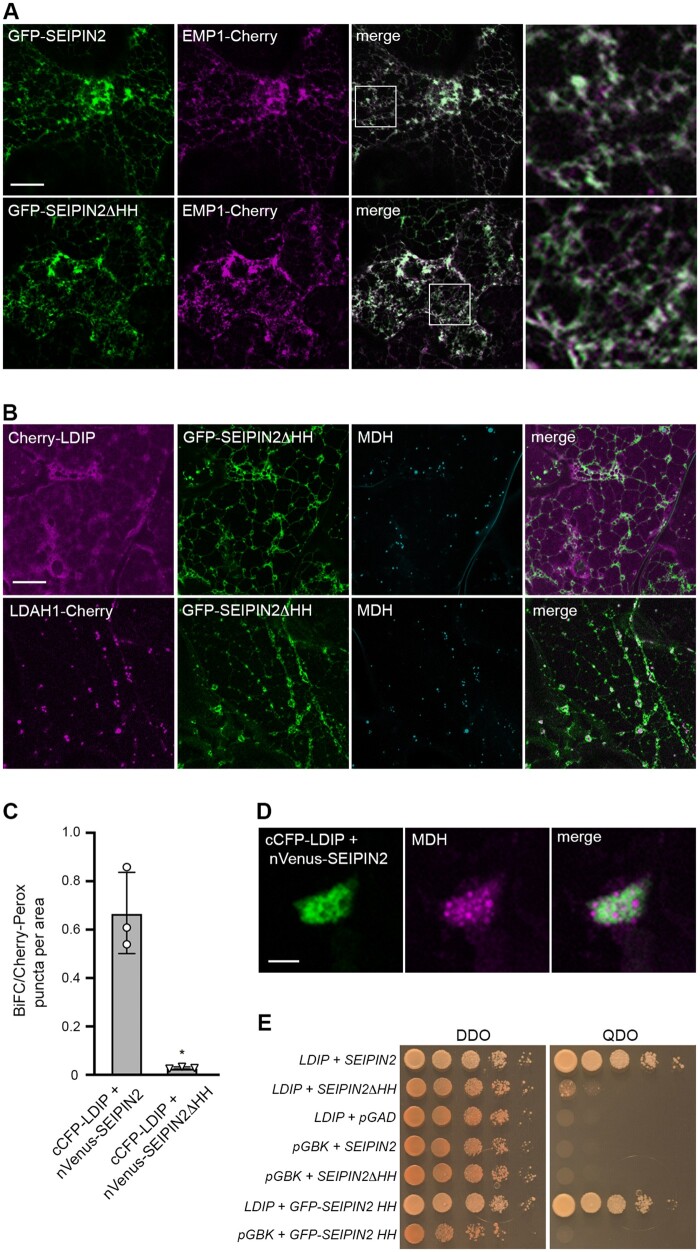
Interaction of LDIP and SEIPIN2 in plant and yeast cells is dependent on the conserved HH sequence in SEIPIN2. A, B, Representative CLSM images (*z*-sections) of *N. benthamiana* leaf epidermal cells co-transformed, as indicated by labels, with either (A) GFP-SEIPIN2 or GFP-SEIPIN2ΔHH (consisting of SEIPIN2 lacking its HH sequence; refer to Supplemental Figure S5) and EMP1-Cherry, serving as an ER marker protein, or (B) Cherry-LDIP or LDAH1-Cherry and GFP-SEIPIN2ΔHH. In (B), LDs were stained with MDH. Shown also are the corresponding merged images. The boxes in the merged images in (A) represent the portion of the cells shown at higher magnification in the panels to the right, highlighting the colocalization of GFP-SEIPIN2 and GFP-SEIPIN2ΔHH with EMP1-Cherry at the ER. Bars in (A) and (B) = 20 µm and applies to all images in the panels, with the exception of those showing a portion of a cell at higher magnification. C, Quantification of BiFC assays with LDIP and SEIPIN2 in *N. benthamiana* leaf cells. Results from 30 areas (i.e. micrographs) of transformed epidermal leaf cells, identified by co-expressed Cherry-Perox fluorescence (serving as a cell transformation marker protein for BiFC assays), were analyzed from three independent experiments (i.e. infiltrations) using the indicated plasmid combinations. Means of the number of BiFC puncta per Cherry-Perox puncta (±sd) per area (micrograph) are shown. Asterisk represents a statistically significant difference (*P* ≤0.05), as determined by a two-tailed Student’s *t* test with Welch’s correction. A summary of the statistical analysis is shown in Supplemental Data Set S1. RT-PCR analysis confirming expression of both pairs of BiFC fusion constructs is shown in Supplemental Figure S2F. D, Representative CLSM images (z-sections) of a region of an individual *N. benthamiana* leaf epidermal cell co-transformed with cCFP-LDIP and nVenus-SEIPIN2. LDs were stained with MDH. Shown also is the corresponding merged image. Note the reticular-like BiFC fluorescence attributable to the interaction of cCFP-LDIP and nVenus-SEIPIN2 and the closely associated MDH-stained LDs; compare with images in Supplemental Figure S4 showing the reorganization of the ER and aggregation of LDs in *N. benthamiana* leaf epidermal cells overexpressing LDIP and SEIPIN proteins and their localization to ER–LD junctions. Bar = 1 µm and applies to all images in the panel. E, Y2H protein interaction analysis of LDIP and SEIPIN2. Full-length Arabidopsis LDIP fused to the Gal4 AD and either full-length SEIPIN2 or mutant versions of SEIPIN2 (i.e. SEIPIN2ΔHH or GFP-SEIPIN2 HH; the latter consisting of the SEIPIN2 HH sequence appended to GFP) fused to the Gal4 BD were co-transformed into yeast (*S. cerevisiae*) cells. Cells were then plated on either plasmid selection conditions (DDO) or higher stringency conditions (QDO) where yeast cell growth is dependent on Y2H protein interactions (see “BiFC and Y2H assays” in “Materials and methods” for additional details). Empty plasmid (negative) controls included pGBK or pGAD. Plasmid combinations are shown to the left and images of the corresponding cell culture serial-dilution series on DDO or QDO plates are shown on the right. Results shown are representative of at least three separate co-transformations of yeast with each plasmid combination

To determine whether plant SEIPINs and LDIP might physically interact in a manner similar to SEIPIN and LDAF1 in mammals, we first deleted the HH sequence from SEIPIN2 (i.e. GFP-SEIPIN2ΔHH, which lacks amino acids 395–416; see Supplemental Figure S5) and evaluated protein localization in *N. benthamiana* leaf cells. As shown in Figure 5A, the co-expression of either GFP-SEIPIN2 or GFP-SEIPIN2ΔHH with Cherry-tagged Arabidopsis ENDOMEMBRANE PROTEIN 1 (EMP1-Cherry), serving as an ER marker protein, resulted in the localization of both proteins at the ER. These results indicate that the deletion of the HH sequence from SEIPIN2 does not disrupt its normal ER targeting in plant cells. However, when GFP-SEIPIN2ΔHH was co-expressed with Cherry-LDIP, the latter protein was not re-localized to the ER, as it was when it was co-expressed with native GFP-SEIPIN2, but instead accumulated in the cytoplasm (Figure 5B, compare with the images of Cherry-LDIP in Figure 4B). That is, Cherry-LDIP yielded a diffuse fluorescence pattern in cells that was distinct from that attributable to co-expressed GFP-SEIPIN2ΔHH at the ER and also the MDH-stained LDs (Figure 5B). On the other hand, the co-expression of GFP-SEIPIN2ΔHH had no obvious effects on the localization of LDAH1-Cherry to LDs (Figure 5B; compare with the localization of LDAH1-Cherry in cells co-expressing GFP-SEIPIN2 in Figure 4C). These results suggest a dominant-negative effect of the SEIPIN2ΔHH protein that is specific for proper targeting of LDIP in plant cells.

Additional support for a physical interaction between LDIP and SEIPIN2 was obtained from bimolecular fluorescence complementation (BiFC) assays in *N. benthamiana* leaves and two-hybrid analysis in yeast. As shown in Figure 5C, the co-expression of cCFP-LDIP and nVenus-SEIPIN2 yielded a BiFC fluorescence signal in *N. benthamiana* leaf cells, but there was significantly less BiFC fluorescence when cCFP-LDIP was co-expressed with nVenus-SEIPIN2ΔHH. Closer analysis of the BiFC fluorescence signal attributable to cCFP-LDIP and nVenus-SEIPIN2 in an individual *N. benthamiana* leaf cell revealed an aggregated and reticular-like structure(s) that was in close association with MDH-stained LDs (Figure 5D), which resembled the reorganization of the ER in cells overexpressing SEIPIN proteins and their localization to ER–LD junctions (Cai et al. 2015; Taurino et al., 2018; Greer et al., 2020; refer also to Supplemental Figure S4). Consistent with the results from BiFC assays, the co-expression of LDIP and SEIPIN2 in the Y2H system resulted in yeast (*Saccharomyces cerevisiae*) cell growth under selective conditions, indicative of a protein–protein interaction (Figure 5E). Yeast cell growth was not observed, however, when LDIP was co-expressed with SEIPIN2ΔHH, nor when LDIP or SEIPIN2 were co-expressed with the corresponding empty vector controls (Figure 5E). Moreover, the co-expression of LDIP with a construct containing just the HH sequence SEIPIN2 alone appended to GFP (i.e. GFP-SEIPIN2 HH), resulted in yeast cell growth. These data together demonstrate that the HH sequence of SEIPIN2 is both necessary and sufficient for interaction with LDIP.

In summary, five lines of evidence support a physical interaction between LDIP and SEIPIN proteins in plant cells, including reciprocal affinity-capture experiments (Figure 4A;  Supplemental Data Set S5; Pyc et al. 2017b), relocalization of LDIP to the ER upon co-expression with SEIPIN (Figure 4B), the loss of LDIP relocalization when the HH sequence of SEIPIN is removed (Figure 5B), and BiFC and Y2H analyses showing that the LDIP and SEIPIN interaction occurs in an HH sequence-dependent manner (Figure 5, C and E).

### Modulating the relative expression of *LDIP* and/or *SEIPIN* influences LD numbers and sizes

**Figure 6:**
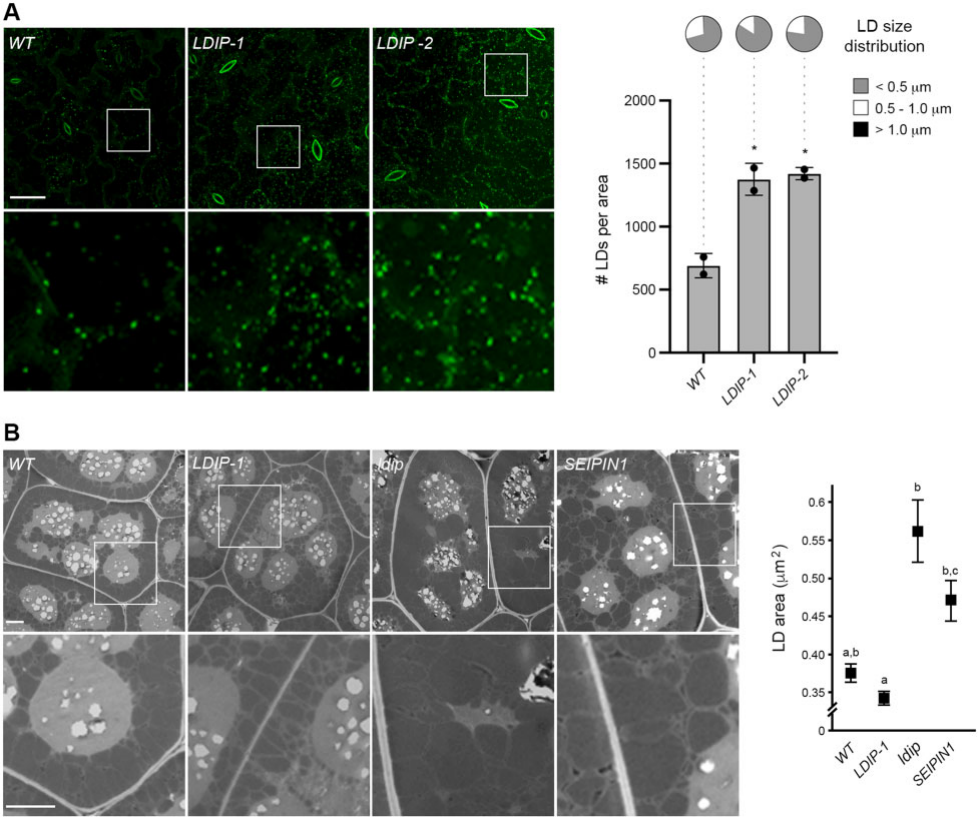
Opposite effects of LDIP and SEIPIN1 in modulating LD size in plant cells. A, LD numbers and sizes in leaves of Arabidopsis *WT* and *LDIP* overexpression transgenic lines. Shown on the left are representative CLSM images (*z*-stacks) of the BODIPY-stained LDs in leaf epidermal and mesophyll cells of 15-day-old seedlings from the *WT* and two independent *LDIP* overexpression lines (i.e. *LDIP-1* and *LDIP-2*), as indicated by labels. Boxes represent the portion of the cells shown at higher magnification in the panels below. Bar = 20 µm and applies to all images in the top row of the panel. Quantifications of LD numbers per area (micrograph) and LD sizes are shown in the graphs on the right. Values of LD numbers are the mean ± sd from three biological replicates, with each replicate consisting of eight leaf samples per line and two micrographs per leaf sample. LD diameters were calculated using the same data set (i.e. micrographs) and are presented as the distribution of LDs in three size classes: <0.5 µm (small), 0.5–1.0 µm (intermediate), and >1.0 µm (large); refer also to key. Asterisks in graph represent statistically significant differences at *P* ≤0.05 relative to the *WT*, as determined by an ANOVA test followed by a Dunnett post hoc multiple comparisons test. A summary of the statistical analysis is shown in Supplemental Data Set S1. Refer to Supplemental Figure S2G for RT-PCR analysis confirming *LDIP* overexpression in both transgenic (*LDIP*-*1* and *LDIP-2*) lines compared to the *WT*. See also Supplemental Figure S1B for violin plots representing the average LD sizes (i.e. LD diameters) in the same lines, based on the data set used here in (A). B, LD sizes in seeds of Arabidopsis *WT* and various transgenic or mutant lines. Shown on the left are representative TEM images of cotyledonary cells in mature, dry seeds from the *WT*, *LDIP-1*, *ldip KO*, and *SEIPIN1* (overexpression) lines, as indicated by labels. Boxes represent the portion of the cells shown at higher magnification in the panels below. Bar = 2 µm and applies to all images in the top row of the panel. Values representing the mean ± sd of LD area for each line are shown in the graph on the right and were calculated based on a data set of manual measurements of LDs in cells from seeds of each line. Statistically significant differences of at least *P* ≤0.01 were determined by an ANOVA test followed by a Tukey’s post hoc multiple comparisons test; refer to Supplemental Data Set S1 for the *P*-value of each group.

To further explore the functional relationships of LDIP and SEIPIN proteins in plant cells, we modulated their relative expression levels in Arabidopsis by overexpressing LDIP and then observing any effects on LD numbers and/or sizes. In *WT* Arabidopsis leaves, LDs are often observed in a fairly narrow range of sizes, with most LDs (∼70%) being small (i.e. <0.5 µm diameter) and the others being either intermediate-sized LDs (i.e. 0.5–1.0 µm [∼29%]) or very few large LDs (i.e. >1 µm [∼0.4%]; Figure 6A). However, the overexpression of LDIP in two independent stable lines (*LDIP-1* and *LDIP-2*) produced a near doubling in the total number of LDs in leaves and with a decrease in average LD size, including a trend toward an increased proportion of smaller LDs at the expense of intermediate-sized LDs (Figure 6A;  Supplemental Figure S1B). Smaller LDs were also observed in Arabidopsis *LDIP-1* seeds compared to LDs in *WT* seeds when analyzed by conventional transmission EM (TEM) (Figure 6B). In contrast, the loss of *ldip* (*KO*) or the overexpression of SEIPIN1 (*SEIPIN1*) in seeds resulted in significant increases in the average LD diameter in both plant lines (Figure 6B).

**Figure 7:**
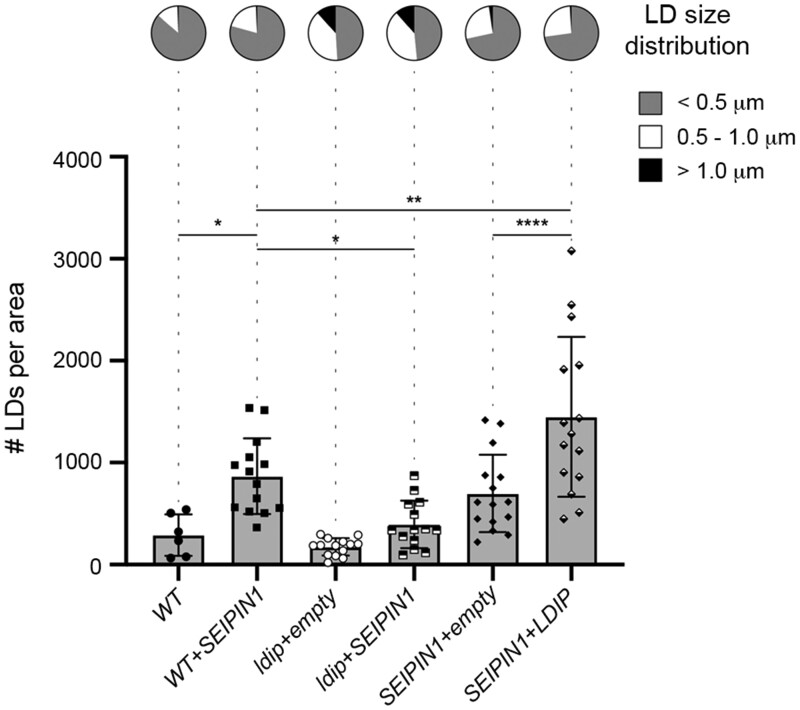
Modulating the relative expression levels of *LDIP* and *SEIPIN1* influences LD numbers and/or sizes in Arabidopsis leaves. Arabidopsis transgenic lines were generated by transforming either *WT* or the *ldip KO* or *SEIPIN1* (overexpression) homozygous parental lines (previously characterized in Pyc et al. (2017a) and Cai et al. (2015), respectively) with plasmids encoding Arabidopsis SEIPIN1 or LDIP, or the corresponding empty vector serving as a control. T_1_ transgenic seedlings were selected on plates based on antibiotic resistance conferred by the introduced transgene-containing vector and then BODIPY-stained LDs in leaf epidermal and mesophyll cells in 28-day-old plants were visualized with CLSM. Values of LD numbers represent the mean ± sd from three biological replicates, with each replicate consisting of three micrographs of two leaf samples from five individual T_1_ plants per line, with the exception of the *WT*, whereby two to four plants from two replicates were examined. LD diameters were calculated using the same data set (i.e. micrographs) and are presented as the distribution of LDs in three size classes: <0.5 µm (small), 0.5–1.0 µm (intermediate), and >1.0 µm (large); refer also to key. *P ≤0.05, **P ≤0.01, or ****P ≤0.0001 in graph represent statistically significant differences as determined by an ANOVA test followed by a Šídák’s post hoc multiple comparisons test. A summary of the statistical analysis is shown in Supplemental Data Set S1. Refer to Supplemental Figure S2H for RT-PCR analysis confirming the relative overexpression or absence of (trans)gene expression for *LDIP* and *SEIPIN1* in selected T_1_ transgenic seedlings compared to the *WT*. See also Supplemental Figure S1C for violin plots representing the average LD sizes (i.e. LD diameters) in the same lines, based on the data set used here in Figure 7.

The similarity in aberrant LD phenotypes observed in *ldip KO* and *SEIPIN1* overexpressing seeds (Figure 6B; Cai et al., 2015; Pyc et al., 2017b; Taurino et al., 2018; Coulon et al., 2020) suggests that relative amounts of LDIP and SEIPIN proteins are important for producing normal-sized LDs in plants, whereby LDIP might act to suppress the formation of larger LDs by SEIPIN1. In this model, the loss of LDIP in the *ldip KO* line would result in the production of enlarged LDs by unregulated SEIPIN1 proteins, while in the *SEIPIN1* overexpression line, the relative amount of SEIPIN1 would be higher than endogenous LDIP, also resulting in the production of larger LDs. Unfortunately, we were unable to generate Arabidopsis plant lines homozygous for both the *ldip KO* and overexpressed *SEIPIN1*, possibly due to embryo death of the progeny. To circumvent this problem, we analyzed LD numbers and sizes in a variety of Arabidopsis T_1_ transgenic plants, which were generated by stably transforming *WT*, *ldip KO* (Pyc et al., 2017b) or *SEIPIN1* (Cai et al., 2015) plants with SEIPIN1 or LDIP, or with the corresponding empty expression vector serving as a control. As shown in Figure 7, compared to the *WT*, the transgenic overexpression of SEIPIN1 in *WT* plants (*WT*+*SEIPIN1*) increased the total number of LDs in leaves, with an increase in average LD size (Supplemental Figure S1C), including a trend towards production of more intermediate-sized LDs, as expected (Cai et al., 2015). The overexpression of SEIPIN1 in the *ldip KO* background line (*ldip+SEIPIN1*), however, increased the total number of LDs, but yielded no obvious alterations in the distributions of LD sizes in comparison to the *ldip+empty* control (Figure 7;  Supplemental Figure S1C). These results suggest that LDIP is indeed important for modulating SEIPIN1’s ability to produce LDs of different sizes in plant cells. However, the transgenic overexpression of LDIP in the *SEIPIN1* overexpression background line (*SEIPIN1+LDIP*) significantly increased LD abundance, but did not alter the distributions of LD sizes, compared to the *SEIPIN1+empty* control (Figure 7;  Supplemental Figure S1C). Taken together, these data suggest that LDIP interacts functionally with SEIPINs to determine the number of LDs in plant cells and, furthermore, that LDIP might work together with SEIPIN to regulate LD size.

### LDIP and SEIPIN work together in *SEIPIN*-disrupted yeast cells to restore the formation of normal numbers and sizes of LDs

**Figure 8:**
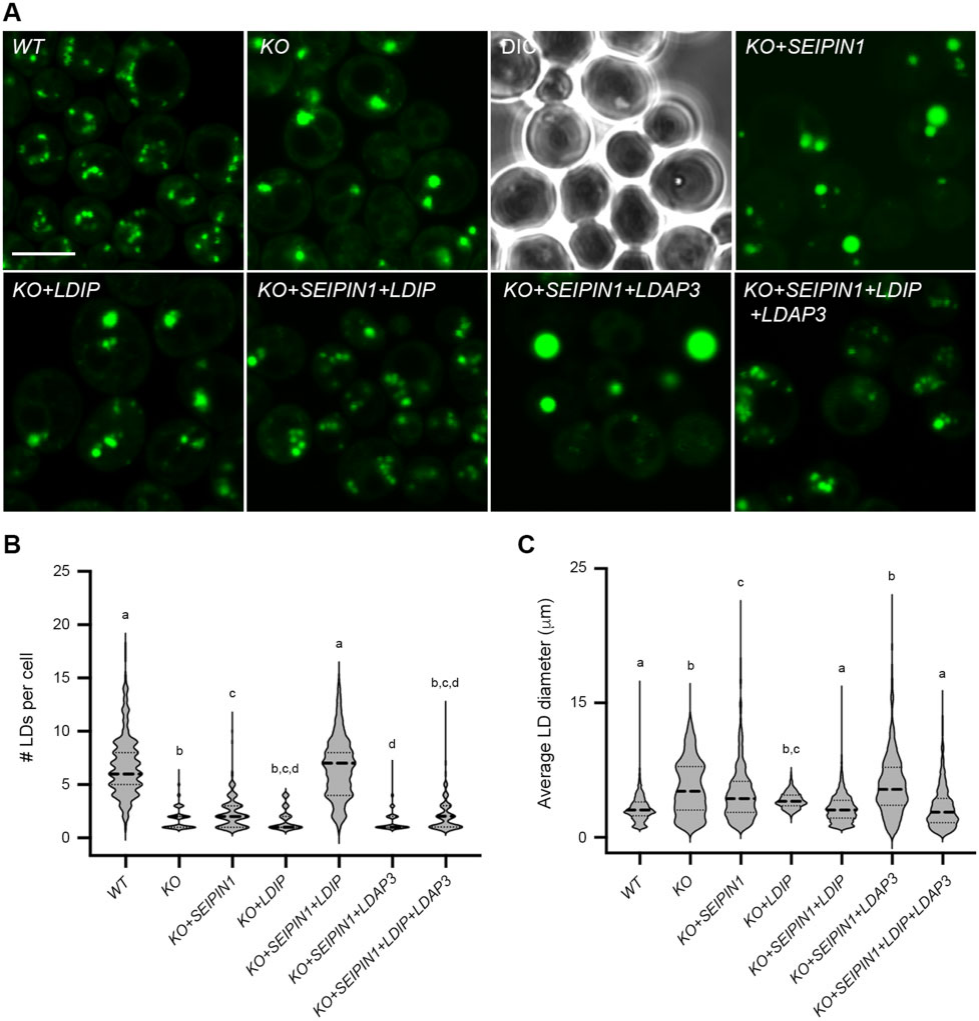
Co-expression of Arabidopsis SEIPIN1 and LDIP in a yeast *SEIPIN*-mutant restores production of normal numbers and sizes of LDs. A, Representative CLSM images (*z*-sections) of either *WT* yeast or yeast cells (*S. cerevisiae*) disrupted in the endogenous *SEIPIN* gene (*KO*), expressing plasmid-borne copies of the indicated Arabidopsis genes, including *SEIPIN1*, *LDIP*, and/or *LDAP3*. Cells were stained with BODIPY and LDs visualized with CLSM; the corresponding differential interference contrast image is also included for the *KO* image. Note the presence of fewer, larger LDs in *KO* yeast cells, and complementation of the LD phenotype only when Arabidopsis SEIPIN1 and LDIP are co-expressed in this mutant background; see main text for additional details. Bar = 5 µm and applies to all images in the panel. B, C, Violin plots showing the numbers and sizes of LDs in each yeast strain, as indicated. Values of LD numbers are averages from >200 cells from at least three separate experiments and LD diameters were calculated using the same data set (i.e. micrographs), including those shown in (A). Dashed and dotted lines represent the median and quartiles, respectively. Significant differences are indicated at least at *P* ≤0.05, as determined by a Kruskal–Wallis test corrected by a Dunn’s multiple comparisons test; refer to Supplemental Data Set S1 for the *P-*value of each group.

To directly assess the roles of LDIP and SEIPIN in modulating LD numbers and sizes in a different cell system, we reconstituted LD biogenesis in a well-characterized yeast (*S. cerevisiae*) *SEIPIN*-disrupted mutant strain (Szymanski et al., 2007; Fei et al., 2008). As shown in the representative confocal microscopy images in Figure 8A and quantified in Figure 8, B and C, and consistent with previous studies (Szymanski et al., 2007; Fei et al., 2008), fewer, but on average, larger-sized LDs were observed in BODIPY-stained *SEIPIN*-disrupted (*KO*) yeast cells, relative to *WT* cells. Similarly, the expression of Arabidopsis SEIPIN1 in the yeast *SEIPIN*-disrupted line (*KO+SEIPIN1*) resulted in an LD phenotype that resembled the *KO* line (Figure 8). The expression of Arabidopsis LDIP in the *KO* line (*KO+LDIP*) also resulted in no obvious effect on the number of LDs and only slight decrease in the average LD size, relative to the *KO* line (Figure 8). On the other hand, the co-expression of SEIPIN1 and LDIP in the *KO* line (*KO+SEIPIN1+LDIP*) yielded an LD phenotype similar to the *WT*, i.e. both the number and average size of LDs were similar in both the *KO+SEIPIN1*+*LDIP* and *WT* cells (Figure 8), indicating that plant SEIPIN and LDIP can function together, but not individually, to restore proper LD biogenesis in yeast.

Given that Arabidopsis SEIPIN1 could produce enlarged LDs in yeast cells (Figure 8; Cai et al. [2015]) and that *S. cerevisiae* appears to lack any obvious homologs to LDIP or LDAF1 (see “Discussion”), we utilized this system to further explore the functional relationships of Arabidopsis SEIPIN1, LDIP, and LDAP in LD biogenesis. The co-expression of SEIPIN1 and LDAP3, but without LDIP, in the yeast *SEIPIN*-disrupted line (*KO+SEIPIN1+LDAP3*) resulted in the appearance of LDs that were fewer and often conspicuously larger than those in the *KO+SEIPIN1* line (Figure 8). These results are remarkably similar to those presented in Figure 1, where fewer and larger LDs were observed in plant cells overexpressing the LDAPs, but lacking LDIP (i.e. *LDAP1/3-Cherry* × *ldip*). However, the co-expression of all three plant proteins together in the yeast *SEIPIN*-disrupted line (*KO+SEIPIN1*+*LDIP*+*LDAP3*), restored production of normal-sized LDs, although there were fewer LDs in comparison to the *WT* (Figure 8). These results suggest there is competition between SEIPIN and LDAP for LDIP binding and that the relative amounts of all three proteins influence the overall number and size of LDs produced in yeast cells.

## Discussion

In recent years, significant advances in our understanding of LD biogenesis in plants have come largely from the identification and characterization of ER-localized, LD biogenetic proteins, such as SEIPINs, as well as LD coat proteins, such as LDAPs and oleosins (reviewed in Pyc et al., 2017a; Huang, 2018; Chapman et al., 2019; Shao et al., 2019; Ischebeck et al., 2020). Here, we show that LDIP serves as a key linchpin that works together with both sets of proteins to coordinate the overall process of LD formation in plant cells. In doing so, the results of this study, as discussed below, help establish a new model of LD biogenesis in plants that is consistent with and extends recent findings on LD biogenesis in eukaryotes more broadly.

### LDIP works together with LDAPs and/or oleosins to modulate LD size and number in plants

There is abundant evidence in the literature showing that LD coat proteins, including LDAPs and oleosins, are important for modulating LD size and/or number in plant cells (Pyc et al., 2017a; Huang, 2018; Shao et al., 2019). For instance, a loss or reduction in the expression of oleosin in Arabidopsis results in an increase in the LD size in seeds (Siloto et al., 2006; Schmidt and Herman, 2008; Miquel et al., 2014), while disruption of any of the three LDAPs in Arabidopsis (i.e. LDAP1–3) results in a decrease in the number of LDs in leaves, but no obvious changes in LD sizes (Gidda et al., 2016). In nitrogen-starved Arabidopsis leaves, however, where LD production is stimulated, the loss of LDAP1 leads to the formation of fewer, but larger LDs in comparison to the *WT* (Brocard et al., 2017). Given that a similar phenotype is observed in Arabidopsis *ldip KO* or *KD* plants (i.e. fewer, larger LDs) and that LDIP contains a discrete LD targeting signal and interacts with LDAPs (Pyc et al., 2017b), we suspected that LDIP might serve as an anchor that recruits LDAPs to the LD surface. The expression of Cherry-tagged LDAPs in *ldip KO* plants, however, revealed that the LDAPs still targeted to LDs in the absence of LDIP (Figure 1A), indicating that LDIP is not required for association of LDAPs with LDs. This premise was supported by proteomic experiments showing that LDAPs, as well as other LD coat proteins (e.g. oleosins), remained associated with LDs in *ldip* mutant plants (Figure 1D). Indeed, additional experiments to reconstitute the interactions between LDIP and LDAP in an insect cell system revealed that LDAP recruits LDIP to the LD surface rather than the other way around (Figure 2).

While LDIP is not required for targeting of LDAPs or oleosins to LDs, LDIP is critically important for regulating LD size and number in a manner that is independent of LDAP or oleosin proteins. For instance, LDAP1- and LDAP3-Cherry both localized to large LDs in leaves of Arabidopsis *ldip* mutant plants (Figure 1A), and LDAPs and oleosins remained associated with large LDs in *ldip* mutant seeds (Figure 1D). Cherry-tagged LDAP3 or oleosin proteins also localized to normal-sized or supersized LDs when *LDIP* expression was modulated by RNAi in *N. benthamiana* leaves (Figure 3). However, proper amounts of both LDIP and LDAP or oleosin are apparently required for formation of normal-sized LDs, since the overexpression of LDIP in *N. benthamiana* leaves was unable to suppress the formation of supersized LDs formed by the co-expression of LEC2 (Figure 3B), a condition where LDAPs and/or oleosin are limiting (Feeney et al., 2013; Kim et al., 2013). Likewise, the overexpression of LDIP in stably transformed Arabidopsis plants resulted in an increase in LD abundance in leaves (Figure 6A) and a greater proportion of smaller LDs in both leaves and seeds (Figure 6, A and B;  Coulon et al., 2020), a condition where the relative amount of LDIP is higher than normal. Thus, it appears that a proper stoichiometric ratio of LDIP and LDAPs or oleosins, as well as SEIPINs (see below), is important for proper LD biogenesis in plant cells.

### LDIP interacts with SEIPIN at the ER

Previous affinity-capture experiments using GFP-LDIP as bait indicated that LDIP interacted not only with itself and LDAPs (and oleosins), but also with the ER-localized SEIPINs (Pyc et al., 2017b). Given the known role of SEIPIN in modulating LD size and number in plant cells (Cai et al., 2015; Taurino et al., 2018), we further explored the potential functional and physical relationships between these proteins. Toward that end, a reciprocal affinity-capture experiment using Arabidopsis GFP-tagged SEIPIN1 as bait indicated that SEIPIN1 interacted with endogenous *N. benthamiana* LDIP (Figure 4A). Surprisingly, the co-expression of Arabidopsis *SEIPIN1* or *SEIPIN2* with *LDIP* in *N. benthamiana* leaves also resulted in a dramatic relocalization of LDIP from LDs to the ER (Figure 4B;  Supplemental Figure S4), suggesting that the localization of LDIP is more dynamic in nature and influenced by relative expression levels of SEIPIN.

The relocalization of LDIP to the ER by the co-expression of SEIPIN (Figure 4B;  Supplemental Figure S4) is similar to recent findings for mammalian SEIPIN and its protein-binding-partner promethin (Castro et al., 2019), which was initially named as such because of its promethin domain (InterPro: PF16015) but is now referred to as LDAF1 (Chung et al., 2019). LDAF1 was originally identified in a screen for genes induced by the peroxisome proliferator-activated receptor γ, which is a transcription factor that regulates adipogenesis and lipid storage in mammals (Yu et al., 2004). A potential functional relationship between LDAF1 and SEIPIN was revealed by a high-throughput affinity-capture screen using human LDAF1 as bait, which identified SEIPIN as an interacting-protein partner (Eisenberg-Bord et al., 2018). Gene expression studies further showed that *LDAF1* and *SEIPIN* genes are similarly expressed in differentiating C3H10T1/2 cells, which is a model cell line for adipogenesis in mammals (Castro et al., 2019). Confocal microscopy analysis revealed that the subcellular localization of LDAF1 is dynamic, being mostly cytoplasmic when mammalian cells are cultured in regular media, but LD-localized when cells were treated with oleic acid to stimulate LD formation (Castro et al., 2019). The co-expression of LDAF1 and SEIPIN in mammalian cells, however, resulted in a dramatic relocalization of LDAF1 from LDs to the ER (Castro et al., 2019), similar to results where we observed the relocalization of LDIP when co-expressed with Arabidopsis SEIPIN1 or SEIPIN2 in plant cells (Figure 4B;  Supplemental Figure S4), suggesting that, in an analogous manner, LDIP and LDAF1 might interact with and function together with SEIPIN at the ER.

LDAF1 was recently shown to be essential for LD formation in mammals (Chung et al., 2019) and insects (Chartschenko et al., 2020). Further, mutagenesis studies indicated that LDAF1 binds specifically to an evolutionarily conserved HH in SEIPIN that is orientated on the lumenal side of the ER membrane, and cryo-EM structural analysis showed that human LDAF1 and SEIPIN form a large, multimeric toroidal-shaped complex (Chung et al., 2019). Consistent with this, plant SEIPIN proteins possess the conserved HH sequence and structural homology modeling studies showed that they can adopt a similar 3D structure as their human or fly counterparts (Chapman et al., 2019; Supplemental Figure S5). Moreover, our results indicate that Arabidopsis SEIPIN2 and LDIP physically interact in an HH-dependent manner (Figure 5). Since LDIP and LDAF1 are short proteins, thus hampering the construction of robust phylogenetic trees for exploring their evolutionary relationship, we examined their sequence homology through other means. That is, using HHpred to query an alignment of plant LDIPs against all the proteins in the human proteome we recovered LDAF1 as the best hit, while querying alignments of LDAFs/promethin-domain-containing proteins from animals and fungi (with the notable exception of *S. cerevisiae*, see below) against the proteome of Arabidopsis recovered an oleosin (also referred to as glycine-rich protein 17) (Supplemental Figure S6C). Thus, while we did not identify LDIP in the latter search, we did identify a known LD-associated protein, namely oleosin (Huang et al., 2018), indicating a relationship between LD proteins of plants and opisthokonts that share a last common ancestor that likely lived more than 1.5 billion years ago (i.e. the last common ancestor of eukaryotes). This warrants further investigation and speaks to a conspicuous similarity and perhaps reflects a deep homology of the components involved in LD formation and function. However, convergence or even more complicated evolutionary scenarios cannot be ruled out.

In mammals, the SEIPIN–LDAF1 complex, but not SEIPIN alone, determines the sites of nascent LD formation at the ER membrane, as defined by the recruitment of perilipin 3 (PLIN3), which is considered among the earliest LD coat proteins associated with LD formation in mammals (Chung et al., 2019). Further, super-resolution confocal microscopy of the LD maturation process in mammalian cells revealed that LDAF1 and SEIPIN initially co-localize at nascent LDs at the ER, and as LD maturation progresses, LDAF1 dissociates from SEIPIN and co-localizes instead with PLIN3 on the LD surface (Chung et al., 2019). If SEIPIN and LDIP function similarly in plant cells, our results would further suggest that the binding of LD coat proteins, such as LDAP, to nascent LDs could help dissociate LDIP from the SEIPIN complex via protein–protein interactions. Prior studies revealed that LDIP and LDAPs physically interacted on the LD surface (Pyc et al., 2017b), and our current experiments with insect cells showed that localization of LDIP to LDs was dependent on co-expression with LDAP3 (Figure 2). Although LDAPs were initially reported as plant-specific proteins (Horn et al., 2013), a remote homology search using alignment of plant LDAP protein sequences against the human proteome identified a PLIN as the best hit (Supplemental Figure S7). Furthermore, a reciprocal search using animal and fungal PLIN protein sequences against the proteome of Arabidopsis identified an LDAP (Supplemental Figure S7). Given this sequence similarity between LDAP and PLIN proteins, and the known physical interaction of LDAPs and LDIP on the LD surface (Pyc et al., 2017b), as well as the results from this study showing the LDAP-dependent targeting of LDIP to LDs (Figure 2), it is plausible that PLIN proteins in animals and fungi might also physically interact with LDAF1 in a manner that promotes dissociation of LDAF1 from the SEIPIN complex and localization to the nascent LD surface.

### LDIP functions with SEIPIN to modulate LD formation in plants and yeast

Disruption of *LDIP* expression in plants or *LDAF1* expression in mammalian cells similarly results in production of fewer, larger LDs (Pyc et al., 2017b; Chung et al., 2019; Figure 1), further suggesting that the two proteins are functionally related. Thus, one role for the LDIP/LDAF1 proteins appears to be their ability to work together with SEIPIN to produce normal numbers and sizes of LDs in cells. Evidence for this in plants comes from our experiments aimed at modulating the relative expression levels of *LDIP* and *SEIPIN1* in Arabidopsis (Figures 6 and 7), which indicated that proper amounts of both proteins are essential for regulating LD numbers and sizes. Additional evidence was obtained in a yeast functional complementation assay, where the expression of Arabidopsis SEIPIN1 in a yeast *SEIPIN*-mutant background resulted in production of fewer, larger LDs (similar to *ldip* mutant plants; Pyc et al., 2017b), while the co-expression of SEIPIN1 and LDIP restored normal numbers and sizes of LDs similar to that in *WT* yeast cells (Figure 8). While there are no obvious homologs to LDAF1/LDIP in *S. cerevisiae*, as well as no proteins annotated to contain a promethin domain, the endogenous yeast SEIPIN protein does have an intimate protein partner in Ldb16 (low dye-binding partner 16; Wang et al., 2014; Grippa et al., 2015; Han et al., 2015). Notably, the loss of Ldb16 phenocopies a *seipin* mutant, revealing that both proteins are required for normal LD biogenesis (Grippa et al., 2015), and they might function together in a manner analogous to mammalian LDAF1 and SEIPIN (Bohnert, 2020). Two additional proteins in *S. cerevisiae*, LD organization proteins of 16 or 45 kDa (Ldo16 and Ldo45, respectively), have also been shown to be important for SEIPIN activity (Esienberg-Bord et al., 2018; Teixeira et al., 2018). Whether any of these additional yeast proteins plays a similar functional role to LDAF1/LDIP remains to be determined.

Although the mechanism by which LDIP or LDAF1 modulates SEIPIN activity to produce normal-sized LDs is currently unknown, there are at least two sets of observations from the literature that shed potential light on this process. First, changes in phospholipid metabolism are often associated with changes in LD size, which are thought to be due to the accumulation of polar lipids in the LD monolayer that either increase or decrease membrane curvature (Fei et al., 2011; Ben M’barek et al., 2017; Choudhary et al., 2018). The SEIPIN complex is known to be important for determining not only the delivery of TAG from the ER into the interior of a nascent, growing LD (Wang et al., 2016; Salo et al., 2019; Prasanna et al., 2021; Zoni et al., 2021), but also the content and composition of the LD monolayer, including both coat protein and phospholipid constituents (Grippa et al., 2015). Perhaps LDIP and LDAF1 function by contributing to a “gatekeeping” mechanism that helps determine the proper monolayer composition of polar lipids, which subsequently influences LD size. Interestingly, the hydrophobic regions of LDIP and LDAF1 proteins have previously been reported to share some similarities with a hydrophobic domain in the mycobacterial membrane protein large (MmpL) family of proteins (Yu et al., 2004; Pyc et al., 2017b), which employ the domain (referred to as an MmpL domain [InterPro: PF03176]) for the transfer of various lipids across the mycobacterial plasma membrane (Viljoen et al., 2017). Perhaps, LDIP/LDAF1 functions in a similar way to transfer the proper phospholipids to the growing monolayer of LDs. However, whether this is the case, or whether LDIP/LDAF1 plays a broader role in modulating some other aspect of phospholipid metabolism, remains to be determined.

A second mechanism known to be involved in determining LD size is the rate of LD initiation at the ER. In yeast cells, deletion of the N-terminal region of SEIPIN results in the production of fewer, larger LDs (Cartwright et al., 2015). By using an inducible LD-formation system, Cartwright et al. (2015) showed that the N-terminal *SEIPIN*-mutant-protein initiated LD formation at the ER at a slower rate than the native SEIPIN protein. However, once LD formation began, LDs produced by the N-terminal *SEIPIN* mutant filled up more quickly, resulting in formation of fewer, but larger LDs than those produced by the native SEIPIN protein. The analysis of LD formation in *LDAF1*-disrupted mammalian cells also revealed a slower rate of LD initiation in comparison to the *WT*, resulting in production of fewer, larger LDs (Chung et al., 2019). Thus, one possible function of LDIP/LDAF1 might be to interact with the SEIPIN complex in a manner that allows for the proper initiation of LD formation at the ER. Notably, TAG amounts were significantly higher in the ER membranes of yeast cells lacking SEIPIN activity (Cartwright et al., 2015). Thus, as reported by Chung et al. (2019), LDAF1/LDIP might interact with SEIPIN in a manner that allows for LD formation at lower ER TAG concentrations. This in turn might be important for maintaining the overall stability and functionality of ER membranes.

### A generalized model for LD biogenesis

**Figure 9:**
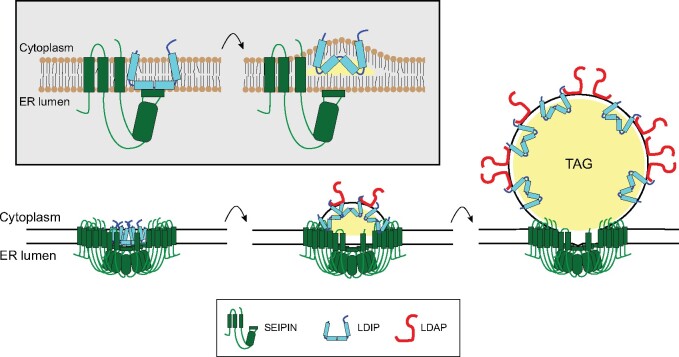
Model for the function of SEIPIN, LDIP, and LDAP in LD biogenesis in plants. The model depicts the association of individual LDIP and SEIPIN proteins at the ER membrane, via LDIP binding to the HH of the SEIPIN protein (refer to model of the Arabidopsis SEIPIN1 protein within the ER membrane in Supplemental Figure S5A), and akin to the interaction of LDAF1 and SEIPIN in mammals. As the TAG “lens” grows, LDIP dissociates from SEIPIN and is stabilized on the LD surface through interaction with LDAP. See main text for additional details. Adapted partly from Chung et al. (2019), Coulon et al. (2020), and Prasanna et al. (2021).

The characterization of the LDAF1/SEIPIN complex in mammals resulted in development of a new and more detailed model of LD biogenesis (Chung et al., 2019; Prasanna et al., 2021; Zoni et al., 2021; reviewed in Thiam and Ikonen, 2021). Given the similarities of SEIPIN/LDAF1 interaction in mammals and SEIPIN/LDIP interaction in plants, we propose a similar model for LD biogenesis in plants (Figure 9). In the initial steps of LD assembly at the ER, LDIP would associate with SEIPIN in a manner similar to how LDAF1 is proposed to interact with SEIPIN in mammals (Chung et al., 2019; Prasanna et al., 2021), wherein LDIP binds to the conserved HH on the ER lumenal side of the SEIPIN protein (Figure 9; refer also to the illustration in Supplemental Figure S5A). Similar also to how LDAF1 is proposed to function (Chung et al., 2019; Prasanna et al., 2021), the hydrophobic segments of LDIP could integrate into the ER bilayer in manner that promotes membrane-bending, akin to ER-shaping reticulon proteins. If plant SEIPINs, like their mammalian counterparts, adopt a radial geometry of 10–12 subunits, as recently proposed from structural homology studies (Chapman et al., 2019), and LDIP associates with SEIPIN in a 1:1 stoichiometry, as shown for mammalian LDAF1 and SEIPIN, the result would be the formation of a large, circular complex of ∼600 kDa, with as many as 72 transmembrane domains (TMDs) present in the complex (i.e. 12 SEIPIN monomers × 2 TMDs each = 24 TMDs; 12 LDIP monomers × 4 TMDs each = 48 TMDs = 72 TMDs total; Figure 9). This large, localized assembly of TMDs would create a hydrophobic pocket that contributes to the accumulation of TAG to form the “lens”-like structures in the ER that are typically observed during early stages of LD biogenesis in mammals (Choudhary et al., 2015; Wang et al., 2016; Zoni et al., 2021). As TAG continues to accumulate within the hydrophobic pocket, LDIP dissociates from SEIPIN, and becomes associated with the nascent LD surface (Figure 9). LDAPs (like PLIN3; Chung et al., 2019) are then recruited to the cytoplasmic surface of the growing ER lens through recognition of membrane “packing defects” that are typical of LD monolayer membranes (Bacle et al., 2017; Prévost et al., 2018; Dhiman et al., 2020). This association of LDAPs with the cytoplasmic surface would help to stabilize the localization of LDIP to the LD surface via protein–protein interactions (Figure 9). The combination of LDAPs and LDIP may also lower membrane surface tension, thereby contributing to the vectorial budding of the nascent LD into the cytoplasm. In seeds and pollen, this process may be assisted by oleosin proteins, which are abundantly distributed from the ER into LDs during LD biogenesis in these cell types (Huang, 2018). The acquisition of additional neutral lipids and phospholipids into the growing LD would then be further mediated by the SEIPIN complex remaining at the ER–LD junction. Targeting of additional LDAPs to the growing LD surface would increase the relative local concentration of LDAP versus SEIPIN proteins, which might serve as a mechanism to modulate the localization of LDIP between the ER-localized SEIPIN and LDs (Figure 9).

While this model is consistent with the experimental evidence collected to date, many questions remain. For instance, do plant SEIPINs form oligomeric, radial structures similar to those observed in yeast, insects, and mammals, and are the complexes composed of mixtures of all three SEIPIN homologs, or are the complexes distinct? How does oleosin work together with SEIPINs, LDIP, and LDAPs to facilitate LD biogenesis in plant cells? Does LDIP modulate polar lipid content and/or composition of the LD monolayer, or polar lipid metabolism, more generally? Alternatively, or in addition to, does LDIP have other functions on LDs beyond biogenesis? Addressing these and other questions should lead to further advances in our understanding of the cellular mechanisms involved in compartmentalization of neutral storage lipids in plants.

## Materials and methods

### Plant materials

All Arabidopsis-based experiments employed the *WT* Columbia Col-0 ecotype or derivatives thereof, including previously described transfer (T)-DNA insertion mutants of *ldip* *KO* and *KD* plants (Pyc et al., 2017b) and *WT*-derived overexpression lines, including *LDAP1-Cherry*, *LDAP3-Cherry*, and *SEIPIN1* (Cai et al., 2015; Gidda et al., 2016). Arabidopsis plants were grown in soil or on plates containing half-strength Murashige and Skoog media (Murashige and Skoog, 1962) in a growth chamber (equipped with T8 bulbs [Sylvania]) at 22°C with a 16-h-day/8-h-night cycle and 50 μE m^−2^ s^−1^ light intensity; with the exception of plants used for LD isolations and proteomics (see below), which were grown under 150 μE m^−2^ s^−1^ light intensity. The *WT* and transgenic plants were grown together at the same time to harvest seed for further studies.

*LDAP1-Cherry* × *ldip* (*KO*) and *LDAP3-Cherry* × *ldip* (*KO*) lines were generated by crossing *ldip KO* with *LDAP1-Cherry* or *LDAP3-Cherry* plants, respectively, and F_1_ progeny were advanced to homozygosity. RT-PCR was used to confirm the expression of LDAP1-Cherry or LDAP3-Cherry and absence of LDIP expression in selected lines (see “RT-PCR and RT-quantitative PCR [RT-qPCR]” for details and refer to Supplemental Figure S2 for all RT-PCR and RT-qPCR results presented in this study). Plants stably overexpressing LDIP were generated using the floral dip method (Clough and Bent, 1998) and *Agrobacterium tumefaciens* strain GV3101 containing the binary plasmid pMDC32/LDIP (see “Plasmid construction” for details), then progeny analysis and RT-PCR were used to select two independent, single-copy, homozygous lines (*LDIP1* and *LDIP2*; Supplemental Figure S2G). Arabidopsis T_1_ plants overexpressing SEIPIN1 in a *ldip KO* or *WT* background or overexpressing LDIP in a *SEIPIN1* (overexpression) background, as well as the same background lines transformed with the corresponding empty vectors, were generated using the floral dip method and binary plasmids pMDC32/SEIPIN1 or pB19/LDIP, or the respective empty vectors, and then selected based on antibiotic resistance conferred by the latter transgene-containing (or empty) vector. After ∼2 weeks on selection, transgenic seedlings were transferred to plates without antibiotic resistance and then grown for another 2 weeks, followed by microscopy analysis of LDs. RT-PCR was used to confirm the overexpression or absence of transgene expression (or endogenous gene expression) in T_1_ seedlings (Supplemental Figure S2H).

*Nicotiana benthamiana* plants used for all *A. tumefaciens*-mediated transient expression experiments were grown in soil in a growth chamber (equipped with T8 bulbs [Sylvania]) at 22°C with a 16-h-day/8-h-night cycle and 50 μE m^−2^ s^−1^ light intensity. Leaves of ∼28-day-old *N. benthamiana* plants were infiltrated with *A. tumefaciens* strain LBA4404 or, for co-immunoprecipitations, strain GV3101, carrying appropriate binary vectors. *A. tumefaciens* transformed with the *Tomato bushy stunt virus* (TBSV) gene *P19* was also included in all infiltrations to enhance transgene expression (Petrie et al., 2010). Detailed procedures for *A. tumefaciens* growth, transformation, infiltration, and processing of *N. benthamiana* leaf material for microscopy have been described previously (McCartney et al., 2005; Cai et al., 2015).

### Plasmid construction

Molecular biology reagents were purchased from New England Biolabs, Thermo Fisher Scientific or Invitrogen, and custom oligonucleotides were synthesized by Sigma-Aldrich. Sequence information for all primers and template DNAs used to construct new plasmids, as described below, are provided in Supplemental Table S3. All plant expression plasmids used in this study were driven by the 35S Cauliflower Mosaic Virus promoter and all newly constructed plasmids were verified using automated DNA sequencing performed at the University of Guelph Genomics Facility or Retrogen Inc.pMDC32/LDIP, encoding nontagged Arabidopsis LDIP, was generated by amplifying (via PCR) the full-length *LDIP* open reading frame (ORF), using pRTL2/Cherry-LDIP (Pyc et al., 2017b) as template DNA and gene-specific primers (Supplemental Table S3). Thereafter, the *LDIP* ORF was subcloned into the pDONR cassette vector yielding pDONR/LDIP, and then the plant expression binary vector pMDC32 using Gateway technology (Curtis and Grossniklaus, 2003). pMDC43/GFP-SEIPIN2ΔHH, encoding GFP linked to the N-terminus of a mutant version of Arabidopsis SEIPIN2 that is missing its conserved, membrane-associated HH sequence (amino acids residues 395–416; refer to Supplemental Figure S5), was generated using PCR-based site-directed mutagenesis with gene-specific primers (Supplemental Table S3) and pDONR/SEIPIN2 (Greer et al., 2020) as template DNA. The coding sequence for SEIPIN2ΔHH (in pDONR/SEIPIN2ΔHH) was then subcloned into pMDC43 using Gateway technology. pB19/LDIP, encoding nontagged LDIP and used for generating Arabidopsis transgenic lines overexpressing LDIP in the *SEIPIN1* overexpression background line, was constructed by PCR-amplifying the Arabidopsis *LDIP* ORF (from pRS316-*PHOSPHOGLYCERATE KINASE* (PGK)/LDIP, see below) with gene-specific primers with flanking 5′ NcoI and 3′ SacII restriction sites (Supplemental Table S3). PCR products were then digested with NcoI and SacII and ligated into similarly digested cloning vector pK83 (Shockey et al., 2015), followed by AscI restriction-enzyme-based cloning into the plant expression vector pB19 (Shockey et al., 2015). Construction of the *N. benthamiana LDIP*-specific RNAi binary vector pB7GW1WG2/NbLDIP RNAi was carried out by PCR-amplifying a conserved region in both of the *N. benthamiana LDIP* genes (nucleotides 61–260 in Niben101Scf06413g00005.1 and Niben101Scf07841g00009.1; sequences and accession numbers based on *N. benthamiana* genome annotation available at Sol Genomics Network (SGN; https://solgenomics.net) using *N. benthamiana* cDNA (derived from mRNA isolated from leaves of 4-week-old plants) as template DNA and gene-specific primers (Supplemental Table S3). Resulting PCR products were then subcloned into pDONR (yielding pDONR/NbLDIP RNAi) and then into the Gateway plant expression RNAi vector pB7GW1WG2 (Karimi et al., 2002). pMDC32/EMP1-Cherry encodes Cherry C-terminal-tagged Arabidopsis EMP1 and was generated by amplifying (via PCR) the full-length *EMP1* ORF, using Arabidopsis cDNA (derived from mRNA isolated from leaves of 4-week-old plants) as template DNA and gene-specific primers (Supplemental Table S3)). Thereafter, the *EMP1* ORF was subcloned into pDONR (yielding pDONR/EMP1) and then pMDC32/ChC (Kretzschmar et al., 2020) using Gateway technology.

Plasmids used for BiFC assays were generated based on the Gateway-compatible vectors pDEST-VYNE/nVenus and pDEST-SCYCE/cCFP, which encode the N- and C-terminal halves of monomeric Venus and cyan fluorescent protein (CFP), respectively (Gehl et al., 2009). Specifically, using Gateway technology, the full-length ORF of Arabidopsis *SEIPIN2ΔHH* was subcloned into pDEST-VYNE/nVenus using pDONR/SEIPIN2ΔHH (see above) as template DNA, while the ORF of Arabidopsis *LDIP* was subcloned into pDEST-SCYCE/cCFP using pDONR/LDIP (see above) as template DNA.

The construction of all other plant expression plasmids used in this study has been described elsewhere, including: pMDC32/LDAP1-Cherry and pMDC32/LDAP3-Cherry, encoding monomeric Cherry C-terminal-tagged Arabidopsis LDAP1 and LDAP3, and OLE1-Cherry, encoding Cherry C-terminal-tagged Arabidopsis oleosin 1 (Gidda et al., 2016); pMDC43/GFP-LDIP and pMDC32/Cherry-LDIP, encoding GFP or Cherry N-terminal-tagged Arabidopsis LDIP (Pyc et al., 2017b); pMDC43/GFP-SEIPIN1 and pMDC43/GFP-SEIPIN2, encoding GFP N-terminal-tagged Arabidopsis SEIPIN1 and SEIPIN2; and pMDC32/SEIPIN1, encoding nontagged Arabidopsis SEIPIN1 (Cai et al., 2015); pDEST-VYNE/nVenus-SEIPIN2, encoding the N-terminal half of Venus fused to Arabidopsis SEIPIN2 (Greer et al., 2020); pMDC32/Cherry-LDAH1, encoding Cherry N-terminal-tagged Arabidopsis LDAH1 (Kretzschmar et al., 2020); pBIN/ER-GK, encoding ER (lumen)-localized GFP and referred to in this study as GFP-ER (obtained from the Arabidopsis Biological Resource Center; Nelson et al., 2007); pORE04-LEC2, encoding LEAFY COTYLEDON 2, a regulator of seed development in Arabidopsis, and pORE04-P19, encoding the *TBSV* RNA-silencing suppressor P19 (Petrie et al., 2010); pMDC32/FIT2-FLL^[157^^–^^159]^AAA, encoding a mutant version of (nontagged) *M. musculus* FIT2 (referred to as FIT2^Mut^ in this study) and whereby the tripeptide -FLL- at positions 157–159 in FIT2 was changed to -AAA- (Cai et al., 2017); and pMDC32/Cherry-PTS1, encoding Cherry linked to type 1 peroxisomal matrix targeting signal (Ching et al., 2012), and referred to as Cherry-Perox in this study.

Plasmids for the expression of plant proteins (or modified versions thereof) in cultured insect cells were constructed using the pIB/V5-His TOPO TA expression kit (Thermo Fisher Scientific), which includes the pIB vector with the constitutive *OplE2* promoter. Briefly, sequences encoding either Venus or Cherry N-terminal-tagged Arabidopsis LDIP, Venus C-terminal-tagged Arabidopsis LDAP3, or nontagged LDAP3 or LDAP3ΔC100 (which encodes a C-terminal 100-amino acid-long truncated version of Arabidopsis LDAP3; Gidda et al., 2016), were generated using overlap extension PCR-based cloning, as described by Fabrick and Hull (2017). All PCR reactions were carried out using the KOD Hot Start DNA polymerase (Sigma-Aldrich), along with the appropriate gene-specific primers and plant expression plasmids as template DNA, including pIB/Venus (Hull et al., 2009), pIB/Cherry (Fabrick and Hull, 2017), pGAD/LDIP, and pGAD/LDAP3 (Pyc et al., 2017b); refer to Supplemental Table S3 for details on primer sequences and template plasmid DNA used for each overlap extension PCR-based cloning step.

For plasmids used in Y2H assays, the full-length Arabidopsis *SEIPIN2* ORF and *SEIPIN2ΔHH* ORF (i.e. encoding SEIPIN2 lacking the HH sequence [amino acids 395–416; refer to Supplemental Figure S5]) were subcloned individually into the “prey” vector pGADT7-AD (which contains the GAL4 activation domain [AD]) (Clontech), using PCR, along with pMDC43/SEIPIN2 (Cai et al., 2015) and pDONR/SEIPIN2ΔHH (see above), respectively, as template DNAs. PCRs also included the appropriate gene-specific primers containing flanking 5′ BamHI and 3′ SacI restriction sites (Supplemental Table S3). Resulting PCR products were then digested with BamHI and SacI and ligated into similarly digested pGADT7-AD, yielding pGAD/SEIPIN2 and pGAD/SEIPIN2ΔHH. pGAD/GFP-SEIPIN2 HH, encoding GFP linked to the N-terminal end of the Arabidopsis SEIPIN2 HH sequence (i.e. amino acids 394–416), was generated by first inserting the HH coding sequence into the MCS of pGADT7-AD using PCR-based site-directed mutagenesis (Q5 Side-Directed Mutagenesis kit; NEB) and pGADT7-AD as template DNA, along with the appropriate primers (Supplemental Table S3). The resulting plasmid, pGAD/SEIPIN2 HH, was then digested with EcoRI and XmaI and ligated with similarly digested PCR products encoding the full-length *GFP* ORF (without a stop codon) which was amplified (via PCR) from pMDC43/mGFP using the appropriate gene-specific primers (Supplemental Table S3), yielding pGAD/GFP-SEIPIN2 HH. The construction of pGBK/LDIP, consisting of the full-length Arabidopsis *LDIP* ORF linked to the GAL4 DNA-binding domain (BD) in the “bait” vector pGBKT7-BD (Clontech), was described in Pyc et al. (2017b).

For complementation studies, yeast expression plasmids encoding Arabidopsis LDIP or LDAP3 were constructed by PCR-amplifying each ORF using gene-specific primers with flanking 5′-BamHI and 3′-EcoRI restriction sites (Supplemental Table S3), and with pMDC32/LDIP and pIB/LDAP3, respectively, as template DNA (see above). Resulting PCR products were digested with BamHI and EcoRI and ligated into the similarly digested pRS-based plasmids, pRS316-PGK or pRS315-PGK, both of which contain the constitutive *PGK1* promoter (Binns et al., 2006), yielding pRS316-PGK1/LDIP and pRS313-PGK1/LDAP3. The construction of pRS315-PGK1/SEIPIN1, encoding Arabidopsis SEIPIN1, was described in Cai et al. (2015).

### Insect cell culture and transfection

*Trichoplusia* *ni* cabbage looper cells (Allele Biotechnology), which are an established culture line derived from *T. ni* ovarian tissue (Hink, 1970), were maintained at 28°C as an adherent monolayer culture in Ex-Cell 420 serum-free insect media (Sigma-Aldrich). Cells were (co)transfected according to Fabrick and Hull (2017) with expression plasmids encoding fluorescent protein-tagged versions of Arabidopsis LDIP and/or LDAP3 (or LDAP3Δ100) and then maintained at 28°C for 48 h for heterologous gene expression. To stimulate LD growth and proliferation, cells were incubated in media supplemented with oleic acid-bovine serum albumin (Sigma-Aldrich) at a final concentration of 400 µM (Thiel et al., 2013). Cells were examined 48-h post-transfection for protein localization and processing for confocal laser-scanning microscopy (CLSM; see “Microscopy”). Polyclonal *T. ni* cell lines stably expressing nontagged Arabidopsis LDAP3 or LDAP3Δ100 were generated by selecting for transgene integration 48-h post-transfection with 250-µg·mL^−^^1^ blasticidin (Thermo Fisher Scientific) for 1 week, then confirming expression by RT-PCR (Supplemental Figure S2B). Stable lines were subsequently maintained in media supplemented with 25 µg·mL^-1^ blasticidin.

### Yeast strains, growth media, and transformation

*Saccharomyces* *cerevisiae* strain BY4742 (*WT*) and the *SEIPIN* gene deletion mutant *ylr404wΔ* derived from this strain were provided by Joel Goodman (Szymanski et al., 2007). All yeast transformations were carried out using the lithium acetate/single-stranded carrier DNA/polyethylene glycol method (Gietz and Schiestl, 2008). For complementation studies, yeast cells were grown in synthetic dextrose (SD) medium containing 2% dextrose, 0.67% yeast nitrogen base, and appropriate amino acid and base supplements (Bufferad), as described elsewhere (Cartwright et al., 2015), and then, when early stationary phase was reached, prepared for CLSM (see “Microscopy” for additional details). For Y2H assays, yeast cells were grown in SD media lacking the appropriate amino acids and then plated on low and high selection growth conditions (see “BiFC and Y2H assays” for additional details).

### BiFC and Y2H assays

BiFC assays in *N. benthamiana* leaves were performed according Pyc et al. (2017b) and guidelines for minimizing protein overexpression artifacts and false positives were taken into consideration, as described elsewhere (Stefano et al., 2015; Kudla and Bock, 2016). Briefly, leaves were co-infiltrated with *Agrobacterium* containing plasmids encoding cCFP-LDIP and nVenus appended to SEIPIN2 or SEIPIN2ΔHH. Infiltrations were also carried out with cCFP alone and nVenus-SEIPIN2. All infiltrations also included Cherry-Perox, which served as a marker for cell transformation. Transformed cells in leaf areas were first detected (via CLSM) based on Cherry fluorescence, and then both Cherry and reconstituted BiFC (cCFP/nVenus) fluorescence signals were collected with identical image acquisition settings for all samples analyzed. The “Analyze Particles” function in ImageJ (v.1.43; https://imagej.nih.gov/ij;  Schneider et al., 2012) was used to quantify Cherry-Perox and BiFC puncta from acquired micrographs of 30 leaf areas from three separate infiltrations.

Y2H assays were carried out as described previously (Greer et al., 2020). Yeast strains (Y2HGold; Clontech) harboring “bait” (pGAD) and “prey” (pGBK) plasmid pairs were plated as serial dilutions on low (double drop-out [DDO]) selection (i.e. SD media lacking tryptophan and leucine) or high (quadruple drop-out [QDO]) selection (same as low selection media, but also lacking histidine and adenine). Drop-out media were purchased from Bufferad Inc. Results of growth assays shown in figures are representative of those obtained from analyzing at least three isolated yeast colonies from three independent transformations.

### RT-PCR and RT-qPCR

Sequence information for all primers used in all RT-PCRs and RT-qPCRs are available in Supplemental Table S3. RNA used for RT-PCR and RT-qPCR was extracted from ∼28-day-old *WT* and transgenic Arabidopsis plants and *Agrobacterium*-infiltrated *N. benthamiana* leaves, as described previously (Cai et al., 2015; Gidda et al., 2016). For RT-PCRs, Arabidopsis β-*TUBULIN* or *N. benthamiana ACTIN* served as reference genes and cycling parameters for Arabidopsis and *N. benthamiana* RNA were 35 cycles (except for *LDIP* RNA, which was 30 cycles) of 95°C for 30 s, 55°C for 30 s, and 72°C for 90 s.

To confirm the expression of transgenes in insect cells using RT-PCR, aliquots of the stable lines and nontransfected cells were pelleted, lysed in TriReagent (Ambion/Thermo Fisher Scientific) by passing through a 22-gage needle, and total RNA isolated with a RNeasy mini kit (Qiagen). cDNAs were generated from 1 µg of DNase I‐treated total RNA using Superscript III reverse transcriptase (Thermo Fisher Scientific) and custom-made random pentadecamer primers. Arabidopsis *LDAP3* and *LDAP3ΔC100* transcripts and *T. ni ACTIN* were amplified using the appropriate primers and Sapphire Amp Fast PCR master mix (Takara Bio USA, Inc.) and the following cycling parameters: 95°C for 2 min followed by 33 cycles at 95°C for 20 s, 56°C for 20 s, 72°C for 20 s, and a final extension at 72°C for 5 min. All RT-PCR products were separated on agarose gels and imaged with a gel documentation system.

RT-qPCRs used to assess the suppression of endogenous *LDIP* in RNAi-infiltrated *N. benthamiana* leaves were performed as follows. cDNAs were generated from isolated RNA (obtained from three separate infiltrations) using a High-Capacity cDNA Reverse Transcription Kit (Applied Biosystems). RT-qPCRs were performed with a QuantStudio 7 Pro Real-Time PCR System (Applied Biosystems) with SsoAdvanced Universal Inhibitor-Tolerant SYBR Green Supermix (Bio-Rad Laboratories) and primers specific for *N. benthamiana LDIP* or *L23* (see Supplemental Table S3), a 60S ribosomal gene, and a well-established *N. benthamiana* reference gene for RT-qPCR (Liu et al., 2012). Cycling parameters were 98°C for 3 min followed by 40 cycles of 98°C for 10 s and 60°C for 30 s. Melt-curve analysis was performed for both *LDIP* and *L23* to confirm that a single RT-qPCR product was amplified. The efficiency of the LDIP primers was also calculated to be 108.8% using a calibration dilution curve. Relative *LDIP* expression was calculated as 2^−^^ΔCt^, where ΔC_t_ = C_t_ (*LDIP*) ^−^ C_t_ (*L23*) and the mean value for each RNAi-untreated sample was normalized to 1.

### Microscopy

The *WT* and transgenic Arabidopsis seeds and leaves, as well as *Agrobacterium*-infiltrated *N. benthamiana* leaves, were processed for CLSM imaging, including staining of LDs either with BODIPY 493/503 (Invitrogen) or MDH (Abgent), as previously described (Cai et al., 2015; Gidda et al., 2016). Micrographs of Arabidopsis and *N. benthamiana* leaves were acquired using either (1) a Leica DM RBE microscope equipped with a 63× Plan Apochromat oil-immersion objective (numerical aperture [NA]=1.32), TCS SP2 scanning head, and three laser systems, including an argon (Ar)-ion laser, and green and red helium–neon (HeNe) lasers; or (2) a Leica SP5 CLSM equipped with a 63× glycerol-immersion objective (NA = 1.3), and five laser systems, including an Ar-ion laser, green, orange, and red HeNe lasers, and a Radius 405-nm laser (Leica Microsystems). Micrographs of dry seeds were acquired with a Zeiss LSM710 with a 63× water-immersion objective lens (NA = 1.15) and an Ar-ion laser (Carl Zeiss Inc.). Images of leaf cells were acquired as single optical sections (i.e. z-sections) or as *z*-stacks (consisting of 0.4-µm *z*-sections, 10-µm thick in total) and, depending on the CLSM system employed, saved as either 512 × 512pixel or 1,024 × 1,024 pixel digital images. Excitations and emission signals for fluorescent proteins and neutral lipid-specific dyes were collected sequentially in double- or triple-labeling experiments are the same as those described previously (Cai et al., 2015; Gidda et al., 2016); single-labeling experiments showed no detectable crossover at the settings used for data collection. All fluorescence images of plant cells shown in individual figures are representative of at least three separate experiments, including at least eight separate (stably transformed) Arabidopsis plants (seedlings), whereby at least 24 leaf areas were analyzed, and at least three separate infiltrations of *N. benthamiana* leaves, whereby more than 25 transformed leaf cells were analyzed. The numbers and diameters of LDs in images of leaves of Arabidopsis seedlings were quantified according to Cai et al. (2015), using the “Analyze Particles” function (at the default settings, with the exception of a circularity value of 0.90–1.0) in ImageJ (v.1.43; https://imagej.nih.gov/ij;  Schneider et al., 2012). Co-localizations of ectopically expressed LDAP1/3-Cherry and BODIPY-stained LDs in leaf cells of stably transformed Arabidopsis plants were quantified based the co-occurrence fraction of Cherry and BODIPY fluorescence, i.e. the Manders’ co-occurrence coefficient, using the ImageJ plugin JACoP (Bolte and Cordelières, 2006).

TEM of Arabidopsis mature seeds was carried out as follows. Seeds were soaked in water for 20 min, then seed coats removed and embryos fixed in 2.5% (w/v) glutaraldehyde, 4% (w/v) paraformaldehyde, 0.01% (v/v) Triton X-100 in 30-mM HEPES buffer (pH 7.4) with 6 × 30 s microwave pulses, and an incubation overnight at 4°C. After 2 h of post-fixation in 1% (w/v) osmium tetroxide at 4°C, and progressive dehydration, inclusion in HM20 Lowicryl resin (Electron Microscopy Services) was performed. Samples were then polymerized under UV light for 48 h. Ultrathin, 70-nm sections were obtained using a Leica EM UC7 ultramicrotome (Leica Microsystems). A FEI Tecnai G2 Spirit TWIN 120kV TEM equipped with a CCD Eagle 4k camera (Raptor Photonics) was used for imaging. Quantification of LD areas in seeds was determined using the ImageJ “Measure” tool (at the default settings) after manually tracing the perimeter of each LD identified in six cells per seed and 2–3 seeds per line (with the exception of the *SEIPIN1* line, whereby three cells per seed were examined) using the ImageJ “Freehand Selection” tool.

LDs in yeast cells were stained with 0.4-µg·mL^-1^ BODIPY 493/503 in 50-mM PIPES buffer, HCS LipidTOX^TM^ Deep Red Neutral Lipid Stain (Thermo Fisher Scientific), or 0.4-µg·mL^-1^ BODIPY 493/503 in IPL-41 insect medium (Thermo Fisher Scientific). LDs were visualized in both cell types using an Olympus FLUOVIEW FV10i CLSM equipped with a 60× water-immersion lens (NA = 1.2), and four laser systems, including an Ar-ion laser, green and red HeNe lasers, and a radius 405-nm laser (Olympus Corp.). All fluorescence images of yeast and insect cells shown in individual figures are single optical sections (0.3 µm) and are representative of >200 and >100 cells for each line, respectively, analyzed from at least three separate experiments (i.e. three independent yeast (co)transformations and insect cell (co)transfections). Subsequent quantification of LD numbers and diameters in 512 × 512 pixel digital images of yeast cells was performed using the “Analyze Particles” function (at the default settings) in the Fiji-plugin image processing package in ImageJ (v.1.52P; Schindelin et al., 2012). All figure compositions were generated and processed for brightness and contrast using Adobe Photoshop CS and Adobe Illustrator (Adobe Systems).

### LD isolations and proteomics

Two hundred and fifty milligrams of Arabidopsis seeds, either *WT*, *ldip* KD, or *ldip KO*, were sterilized and stratified for 3 days in the dark at 4°C and then grown for 40 hr on plates containing half-strength Murashige and Skoog media (see “Plant materials” for additional details). LD-enriched fractions and total cellular fractions were obtained, and proteins extracted as described previously (Kretzschmar et al., 2020; see also Kretzschmar et al., 2018). Proteins were isolated, their concentrations determined, and then subjected to in-gel tryptic digestion (Kretzschmar et al., 2020). The peptide purification, liquid chromatography-tandem mass spectrometry (MS/MS), and protein analysis, including calculated protein levels based on the label-free quantification and intensity-based quantification algorithms with MaxQuant software (v. 1.6.2.10; Cox and Mann, 2008; Cox et al., 2014), were performed exactly as described in Kretzschmar et al. (2020). All LD proteomics data are shown in Supplemental Data Sets S2–S4 and available also through the ProteomeXchange Consortium via the PRIDE partner repository (https://www.ebi.ac.uk/pride/;  Perez-Riverol et al., 2019), under the project accession number PXD012992; refer to metadata in Supplemental Table S1.

### Affinity-capture of GFP-SEIPIN1 expressed in *N. benthamiana* leaves

Co-immunoprecipitation assays with GFP-Trap agarose, consisting of Alpaca (*Vicugna pacos*) anti-GFP V_H_H, purified antibodies coupled to agarose beads (ChromoTek GmbH), and extracts from *A. tumefaciens*-infiltrated *N. benthamiana* leaves expressing either GFP alone or GFP-SEIPIN1, with or without co-expressed Arabidopsis LEC2, were carried out as described previously for co-immunoprecipitations with GFP-LDIP, with and without LEC (Pyc et al., 2017b; see also Cai et al. (2019) and Greer et al. (2020) for additional details on co-immunoprecipitation procedures). GFP-SEIPIN1 co-purifying proteins were concentrated in the top of an SDS–PAGE resolving gel and the Coomassie blue-stained protein bands were excised and submitted to the Michigan State University Proteomics Core Facility (https://rtsf.natsci.msu.edu/proteomics/) for subsequent processing of samples for MS analysis, as described previously (Pyc et al., 2017b; Cai et al., 2019). All co-precipitating proteins identified in GFP-SEIPIN1 affinity-capture experiments, as well as those identified in previous affinity-capture experiments using GFP-LDIP (Pyc et al. (2017b), are shown in Supplemental Data Set S5. These data are available also through the ProteomeXchange Consortium via the PRIDE partner repository (https://www.ebi.ac.uk/pride/), under the project accession number PXD023043. Refer to Supplemental Table S2 for additional details on accessing the MS data for both GFP-SEIPIN1 and GFP-LDIP affinity-capture experiments.

### Bioinformatics

Multiple sequence alignments of SEIPIN and LDIP proteins were generated using the Clustal Omega tool at the European Bioinformatics Institute (https://www.ebi.ac.uk/Tools/msa/clustalo/; Madeira et al., 2019). Putative TMDs in polypeptide sequences were identified using the TMHMM (v.2.0) server (http://www.cbs.dtu.dk/services/TMHMM/;  Krogh et al., 2001) or the Constrained Consensus TOPology prediction server (http://cctop.enzim.ttk.mta.hu; Dobson et al., 2015). Protein domain structures of Arabidopsis LDIP and *H. sapiens* LDAF1 were based on the UniProt/InterPro database (https://www.ebi.ac.uk/interpro/; UniProt Consortium, 2020). Sequences of LDIP (PTHR35508-domain-containing), LDAF1 (promethin [PF16015]-domain-containing), LDAP, and PLIN homologs used for multiple alignment using fast Fourier transform-based (with the G-INS-I option; https://mafft.cbrc.jp; Katoh and Standley, 2013) alignments were obtained from UniProt/InterPro, and also (for plant LDIP [PTHR35508-domain-containing] proteins) from the genomes of *Anthoceros agrestis*, *Azolla filiculoides*, *Brassica rapa*, *Gnetum montanum*, *Gossypium hirsutum*, *N. tabacum*, *Oryza sativa*, *Physcomitrella patens*, and *Theobroma cacao*. Pairwise comparison of profile hidden Markov model searches were carried out using the HHpred server at the MPI Bioinformatics Toolkit (https://toolkit.tuebingen.mpg.de/tools/hhpred; Zimmermann et al., 2018) against the proteome of *H. sapiens or A. thaliana* and using the Protein Data Bank (https://www.rcsb.org) as structural/domain database and default parameters.

To generate the model of Arabidopsis SEIPIN1 and conservation of the HH sequence in plant SEIPIN1 proteins, as shown in Supplemental Figure S5A, multiple sequence alignment was performed using the Clustal Omega with 96 sequences of plant (taxid: 3193) SEIPIN1 protein homologs recovered by BLAST with Arabidopsis SEIPIN1 as the query. The sequence logo of the conserved membrane-associated HH in SEIPIN1 proteins was extracted from a motif identified using the Multiple Em for Motif Elicitation suite (https://meme-suite.org/meme/tools/meme;  Bailey et al., 2015). The motif count was set to “3” with a minimum and maximum width set between “10 and 200” residues. No motif *E*-value threshold was assigned and the minimum site per motif was set to “96”. This nonbiased method identified a 56-amino acid-long motif containing the membrane-associated HH with an *E*-value of 3.7e4158.

### Statistical analysis

All statistical analyses were performed using GraphPad Prism (v.9.1) (www.graphpad.com). Significant differences were determined by an analysis of variance test followed by (1) a Dunnett or Šídák post hoc multiple comparisons test for LD numbers per area in Arabidopsis leaves or (2) a Tukey post hoc multiple comparisons test for LD sizes in Arabidopsis seeds. For LD size quantifications in Arabidopsis leaves and LD numbers and size quantifications in yeast, a Kruskal–Wallis test corrected by a Dunn’s multiple comparisons test was used. BiFC and RT-qPCR data were analyzed using two-tailed Student’s *t* tests with a Welch’s correction. Summaries of all the statistical analysis data are available in Supplemental Data Set S1.

### Accession numbers

Accession numbers, based on The Arabidopsis Information Resource (https://www.arabidopsis.org), National Center for Biotechnology Information (https://www.ncbi.nlm.nih.gov), UniProt, or SGN, for all proteins examined in this study are as follows: Arabidopsis EMP1 (At5g10840); LDAH1 (At1g10740), LDAP1, (At1g67360), LDAP3 (At3g05500), LDIP (At5g16550), LEC2 (At1g28300), OLE1 (At4g25140), SEIPIN1 (At5g16460), SEIPIN2 (At1g29760) and β-TUBULIN (At5g44340); *D. melanogaster* SEIPIN (Q9V3X4); *H. sapiens* SEIPIN (Q96G87), LDAF1 (NP_001288700.1); *M. musculus* FIT2 (EMBL Accession No. BAE37420.1); *N. benthamiana* ACTIN (AY179605.1) and LDIP (Niben101Scf06413g00005.1 and Niben101Scf07841g00009.1); *T. ni* ACTIN (JF303662); TBSV P19 (CAC01278,1).

## Supplemental data

The following materials are available in the online version of this article.

**Supplemental Figure S1**. Violin plots showing the sizes of LDs in the *WT* and various transgenic Arabidopsis lines.

**Supplemental Figure S2**. Confirmation of the expression of transgenes or the suppression of *LDIP* in plants and/or insect cells.

**Supplemental Figure S3**. LDIP, unlike LDAP3, does not compartmentalize neutral lipids into normal-sized LDs in leaves co-expressing mouse FIT2^Mut^.

**Supplemental Figure S4**. Colocalization of LDIP, SEIPIN, and LDs in *N. benthamiana* leaf cells.

**Supplemental Figure S5**. Characterization of the HH sequence in plant SEIPINs.

**Supplemental Figure S6**. Similarity of Arabidopsis LDIP and human LDAF1.

**Supplemental Figure S7**. Phylogenetic relationship of plant LDAP and animal PLIN proteins.

**Supplemental Table S1**. Metadata file for liquid chromatography-tandem mass spectrometry processing of the *WT* and *ldip* mutant LD proteomics with MaxQuant.

**Supplemental Table S2**. Access information to raw MS data for GFP-SEIPIN1 and GFP-LDIP (with or without co-expressed Arabidopsis LEC2) affinity-capture experiments.

**Supplemental Table S3**. Names and sequences of oligonucleotide primers used in this study.

**Supplemental Data Set S1**. Reports from statistical tests performed in GraphPad Prism.

**Supplemental Data Set S2**. Raw quantitative *WT* and *ldip* mutant LD proteomics data.

**Supplemental Data Set S3**. Normalized quantitative *WT* and *ldip* mutant LD proteomics data.

**Supplemental Data Set S4**. Normalized quantitative proteomics data of the *WT* and *ldip* mutant LD-associated proteins.

**Supplemental Data Set S5**. List of *N. benthamiana* proteins co-immunoprecipitated with either GFP-SEIPIN1 or GFP-LDIP and with or without co-expressed Arabidopsis LEC2.

**Supplemental Data Set S6**. List of 15 land plant PTHR35508-domain-containing protein sequences.

**Supplemental Data Set S7**. List of 320 animal promethin (PF16015)-domain-containing protein sequences.

**Supplemental Data Set S8**. Proteins identified in an HHpred search of the *H. sapiens* proteome using an alignment of 15 plant PTHR35508-domain-containing protein sequences.

**Supplemental Data Set S9**. Proteins identified in a HHpred search of the Arabidopsis proteome using an alignment of 320 animal promethin (PF16015)-domain-containing protein.

**Supplemental Data Set S10**. List of 75 animal and fungal N-terminal PLIN protein sequences.

**Supplemental Data Set S11**. Proteins identified in an HHpred search of the Arabidopsis proteome using an alignment of 75 animal and fungal N-terminal PLIN protein sequences.

**Supplemental Data Set S12**. List of 53 angiosperm and gymnosperm LDAP sequences.

**Supplemental Data Set S13**. Proteins identified in an HHpred search of the *H. sapiens* proteome using an alignment of 53 angiosperm and gymnosperm LDAP sequences.

## Supplementary Material

koab179_Supplementary_Data

## Notes

M.P., S.K.G., D.S., F.K.K., Y.C., N.M.D., M.S.G., J.J.H., D.C., N.E., C.B. and T.I. performed the experiments. O.V. and G.H.B. performed LD proteomics and J.d.V. performed LDIP/LDAF and LDAP/PLIN phylogenetic analyses. M.P., R.T.M., J.M.D., and K.D.C. conceptualized through initial discussions the potential for interactions among SEIPINS, LDIP, and LDAP. J.M.D. and R.T.M. prepared the first and revised drafts of the manuscript with input from M.P., T.I., and K.D.C. All the authors contributed to editing the manuscript and have read and approved the final version.

The author responsible for distribution of materials integral to the findings presented in this article in accordance with the policy described in the Instructions for Authors (https://academic.oup.com/plcell) is: Robert T. Mullen (rtmullen@uoguelph.ca).
