# Supplementary material for: LDIP cooperates with SEIPIN and LDAP to facilitate lipid droplet biogenesis in Arabidopsis
Source: Plant Cell. 2021 Jun 9;33(9):3076–103. doi: 10.1093/plcell/koab179 (PMC8462815; doi:10.1093/plcell/koab179)
Supplement: koab179_Supplementary_Data [file koab179_supplementary_data.zip › tpc.00378.2021-s01.pdf]

Supplemental Data. Pyc and Gidda et al. (2021). LDIP Cooperates with SEIPIN and LDAP to Facilitate Lipid Droplet Biogenesis in Arabidopsis. Plant Cell.

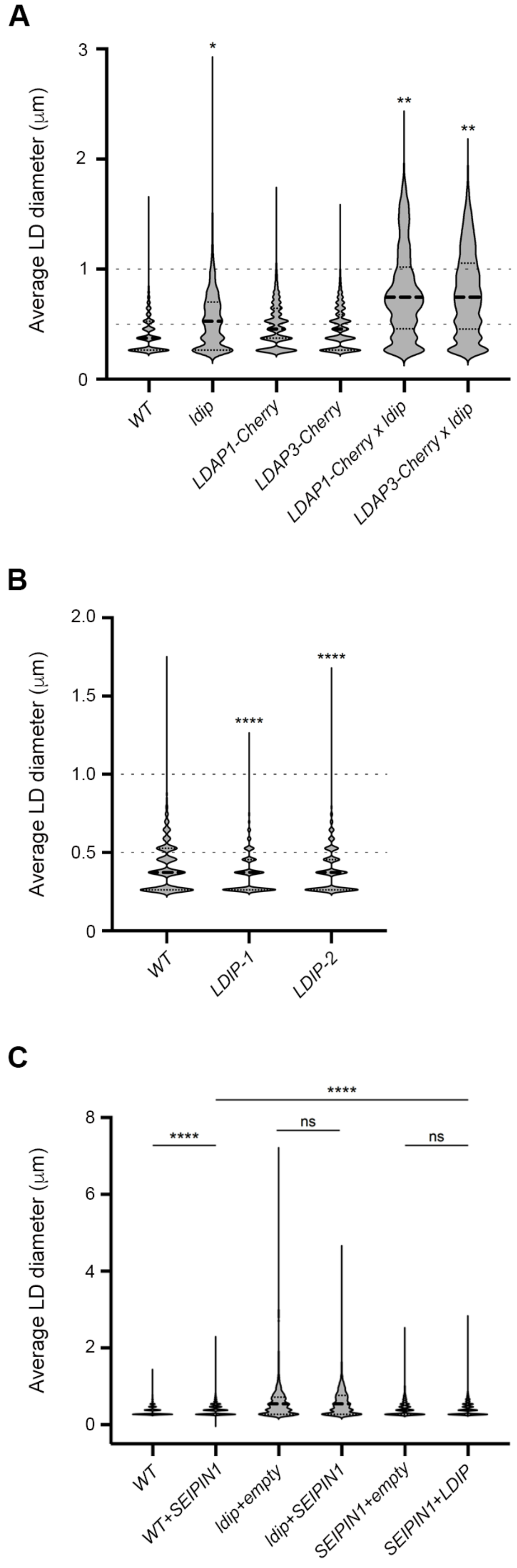

Supplemental Data. Pyc and Gidda et al. (2021). LDIP Cooperates with SEIPIN and LDAP to Facilitate Lipid Droplet Biogenesis in Arabidopsis. Plant Cell.

Supplemental Figure S1. Violin plots showing the sizes of LDs in *WT* and various transgenic Arabidopsis lines. Supports Figure 1, 6 and 7.
(A) and **(B)** Values of LD sizes (i.e., diameters) in **(A)** and **(B)** were calculated using the same data set as those used in Figure 1B and 6A, respectively. Values are the averages ± SD from three biological replicates, with each replicate consisting of eight leaf samples per line and two micrographs per leaf sample. Dashed and dotted lines represent the median and quartiles, respectively. Asterisks in graphs represent statistically significant differences in **(A)** at $P \leq 0.05$ (*) and $P \leq 0.01$ (**) relative to *WT* and *LDAP1/3-Cherry* lines, respectively, or in **(B)** at $P \leq 0.0001$ (****) relative to *WT*, as determined by a Kruskal-Wallis test followed by a Dunnett post-hoc multiple comparisons test. A summary of the statistical analysis is shown in Supplemental Dataset S1.
(C) Values of LD sizes (i.e., diameters) were calculated using the same data set as used in Figure 7. Values are the averages ± SD from three biological replicates, with each replicate consisting of three micrographs of two leaf samples from 5 individual T_1 plants per line, with the exception of *WT*, whereby 2–4 plants from two replicates were examined. Dashed and dotted lines represent the median and quartiles, respectively. Asterisks represent statistically significant differences at $P \leq 0.0001$, as determined by a Kruskal-Wallis test followed by a Dunnett post-hoc multiple comparisons test; ns, not significant. A summary of the statistical analysis is shown in Supplemental Dataset S1.

Supplemental Data. Pyc and Gidda et al. (2021). LDIP Cooperates with SEIPIN and LDAP to Facilitate Lipid Droplet Biogenesis in Arabidopsis. Plant Cell.

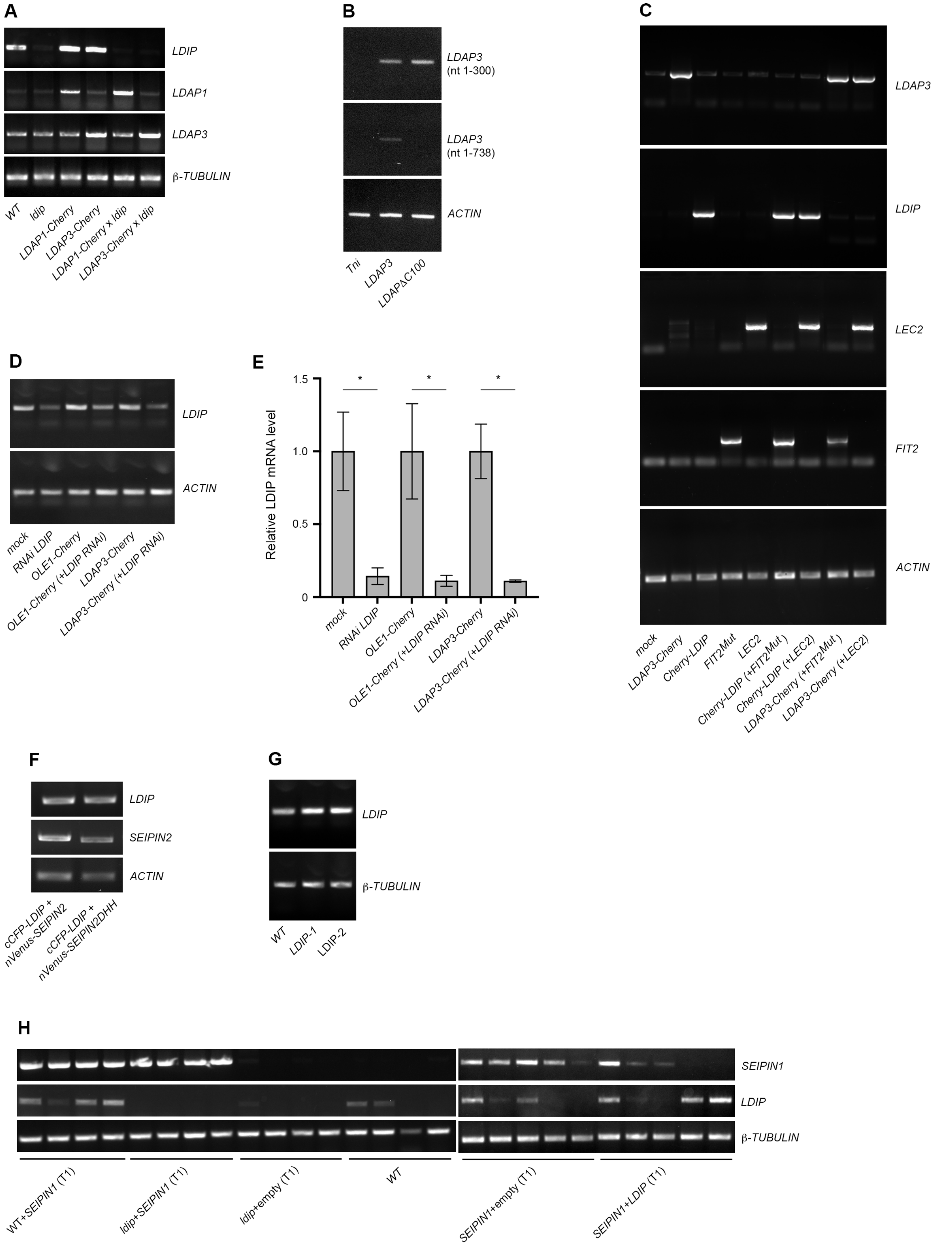

Supplemental Figure S2. Confirmation of expression of transgenes or suppression of *LDIP* in plants and/or insect cells. Supports Figure 1, 2, 3, 5, 6 and 7, as well as Supplemental Figure S3.

(A) RT-PCR analysis of crossed *Cherry-LDAP1/3* x *ldip* Arabidopsis lines. RNA was isolated from leaves from *WT*, parental homozygous *LDAP1-Cherry* and *LDAP3-Cherry* overexpression lines and the *ldip KO* line (described previously in Gidda et al. [2016] and Pyc et al. [2017b], respectively), and crossed *LDAP1/3-Cherry* x *ldip* plants, and then subjected to RT-PCR with gene-specific primers, or with primers for endogenous β-*TUBULIN*, which served as a reference gene. Details on primers used in all RT-PCRs and RT-qPCRs described in **(A)**–**(G)** are available in Supplemental Table S3.

(B) RT-PCR analysis of *LDAP3* and *LDAP∆C100* expression in insect cell lines. RNA was isolated from non-transformed *T. ni* cultured cells (Tni) and polyclonal cell lines stably expressing either Arabidopsis full-length *LDAP3* or C-terminal-truncated LDAP3 (*LDAP3∆C100*). RNA was then subjected to RT-PCR using *LDAP3*-specific primers, including primer pairs designed to amplify nucleotides (nt) 1–300 or 1–738 in the *LDAP3* ORF, or with primers for endogenous *ACTIN*, which served as a reference gene.

(C) RT-PCR analysis of transgene expression in *N. benthamiana* leaves. RNA was isolated from leaves infiltrated with the indicated constructs (or mock infiltrated), including, with or without LEC2 or $FIT2^{Mut}$. RNA was then subjected to RT-PCR using gene-specific primers, or with primers for endogenous *ACTIN*.

(D) RT-PCR analysis of the suppression of endogenous *LDIP* expression in *N. benthamiana* leaves via RNAi. RNA was isolated from leaves infiltrated with the indicated constructs (or mock infiltrated), including, with or without *LDIP* RNAi. RNA was then subjected to RT-PCR using endogenous *LDIP*-specific primers or with primers for *ACTIN*. Refer to **(E)** for quantification of endogenous *LDIP* suppression using RT-qPCR.

(E) RT-qPCR analysis of *LDIP* RNAi-infiltrated *N. benthamiana* leaves. As above in **(D)**, RNA was isolated from leaves infiltrated with the indicated constructs (or mock infiltrated), including with or without *LDIP* RNAi. RNA was then subjected to RT-qPCR using endogenous *LDIP*-specific primers, or with primers specific for endogenous *L23,* serving as reference gene (Liu et al., 2012). Relative *LDIP* expression is shown normalized to each of the samples infiltrated without LDIP RNAi. Relative *LDIP* expression values are averages ± SD from three biological replicates, each biological replicate consisting of a separate infiltration and RNA isolation. Asterisks represent statistically significant differences ($P \leq 0.05$), as determined by a two-tailed Student's *t*-test with Welch's correction. A summary of the statistical analysis is shown in Supplemental Dataset S1.

(F) RT-PCR analysis of *BiFC* transgene expression in *N. benthamiana* leaves. RNA was isolated from leaves infiltrated with the indicated LDIP and SEIPIN2 (or 'empty') BIFC constructs. RNA was then subjected to RT-PCR using gene fusion-specific primers, or with primers for endogenous *ACTIN.*

(G) RT-PCR analysis of Arabidopsis *LDIP* overexpressing lines. RNA was isolated from leaves from *WT* or the two independent, single-copy homozygous *LDIP* overexpressing (i.e., *LDIP-1 and LDIP-2*) transgenic lines, and then subjected to RT-PCR with *LDIP*-specific primers, or with primers for endogenous β-*TUBULIN*.

(H) RT-PCR analysis of gene expression in various Arabidopsis T_1 plants. RNA was isolated from leaves from 4–5 selected first generation (T_1) plants that were generated by stably transforming *WT* or the *ldip KO* or *SEIPIN1* overexpressing, homozygous parental lines (described previously in Pyc et al. [2017b] and Cai et al. [2015], respectively) with either Arabidopsis SEIPIN1 or LDIP, or an empty vector serving as a control. RNA was then subjected to RT-PCR with *LDIP-* or *SEIPIN1*-specific primers, or with primers for endogenous β-*TUBULIN.*

Supplemental Data. Pyc and Gidda et al. (2021). LDIP Cooperates with SEIPIN and LDAP to Facilitate Lipid Droplet Biogenesis in Arabidopsis. Plant Cell.

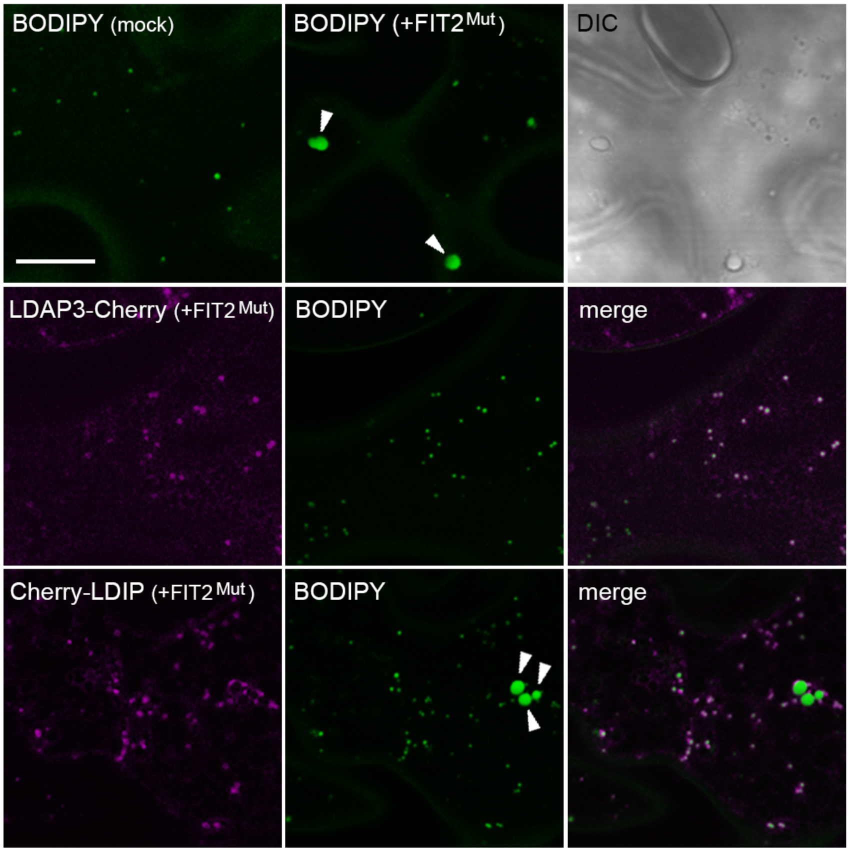

Supplemental Figure S3. LDIP, unlike LDAP3, does not compartmentalize neutral lipids into normal-sized LDs in leaves co-expressing mouse FIT2^{Mut}. Supports Figure 3.

Representative CLSM images (z-sections) of *N. benthamiana* leaf epidermal cells mock transformed or transiently (co)transformed, as indicated by labels, with either Cherry-tagged LDAP3 or LDIP, along with a mutant version of the mouse LD biogenetic protein FIT2 (FIT2^{Mut}), which produces supersized LDs when ectopically expressed in plant cells (Cai et al., 2017). All cells were also transformed with P19, serving as a suppressor of gene silencing (Petrie et al., 2010), and LDs in cells were stained with BODIPY. Note the presence of aberrant, supersized LDs (indicated with arrowheads) in cells transformed with either FIT2^{Mut} alone, as previously reported (Cai et al., 2017), or with Cherry-LDIP and FIT2^{Mut}. By contrast, mock-transformed cells or those co-expressing LDAP3-Cherry with FIT2^{Mut} possess normal-sized LDs. Shown also is the corresponding DIC image of the cell transformed with FIT2^{Mut} alone. RT-PCR analysis confirming transgene expression in all samples are presented in Supplemental Figure S2C. Bar = 20 μm and applies to all images in the panel.

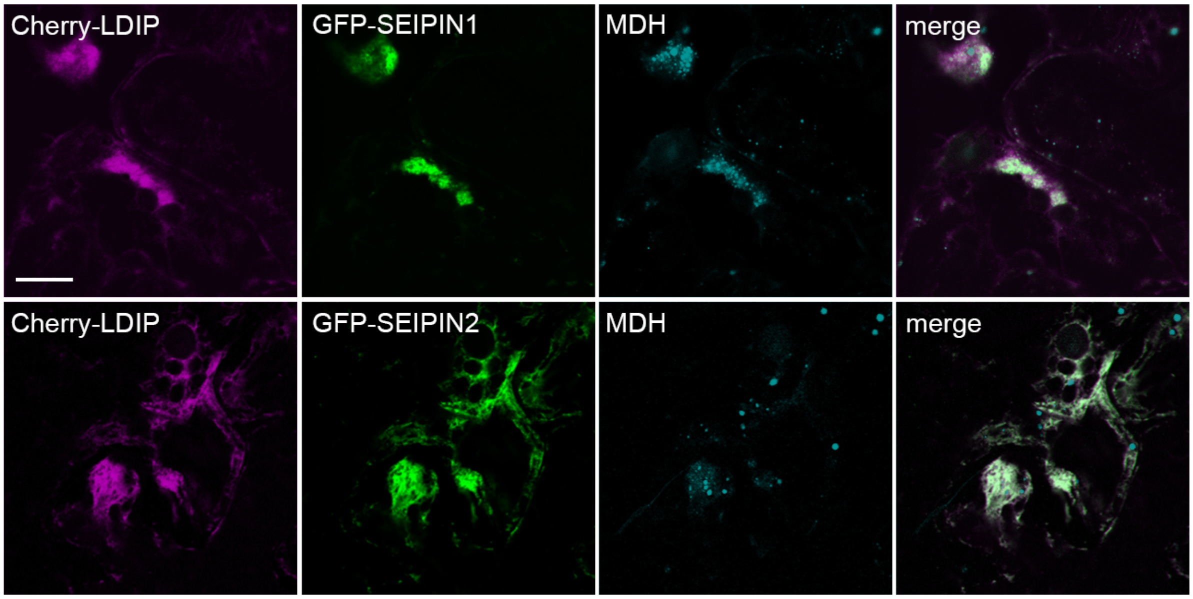

Supplemental Figure S4. Colocalization of LDIP, SEIPIN and LDs in *N. benthamiana* leaf cells. Supports Figure 4.

Representative CLSM images (z-sections) of *N. benthamiana* leaf epidermal cells (co)transformed, as indicated by labels, with Cherry-LDIP and either GFP-SEIPIN1 or GFP-SEIPIN2. LDs were stained with MDH. Shown also are the corresponding merged images. Bar = 20 μm and applies to all images in the panel. Note the colocalization of Cherry-LDIP and GFP-SEIPIN1/2 at the ER and the closely-associated, aggregated LDs, as well as the reorganization of the ER due to the overexpression of SEIPIN1/2, as previously reported (Cai et al. 2015; Taurino et al., 2018; Greer et al., 2020); compare with images presented in Figure 4B; here with Cherry-LDIP and GFP SEIPIN1/2 (co)expressed for a longer time period (i.e., 5-d post-infiltration, rather than 3-d post-infiltration, as in Figure 4B). These data provide results that support those in Figure 4.

Supplemental Data. Pyc and Gidda et al. (2021). LDIP Cooperates with SEIPIN and LDAP to Facilitate Lipid Droplet Biogenesis in Arabidopsis. Plant Cell.

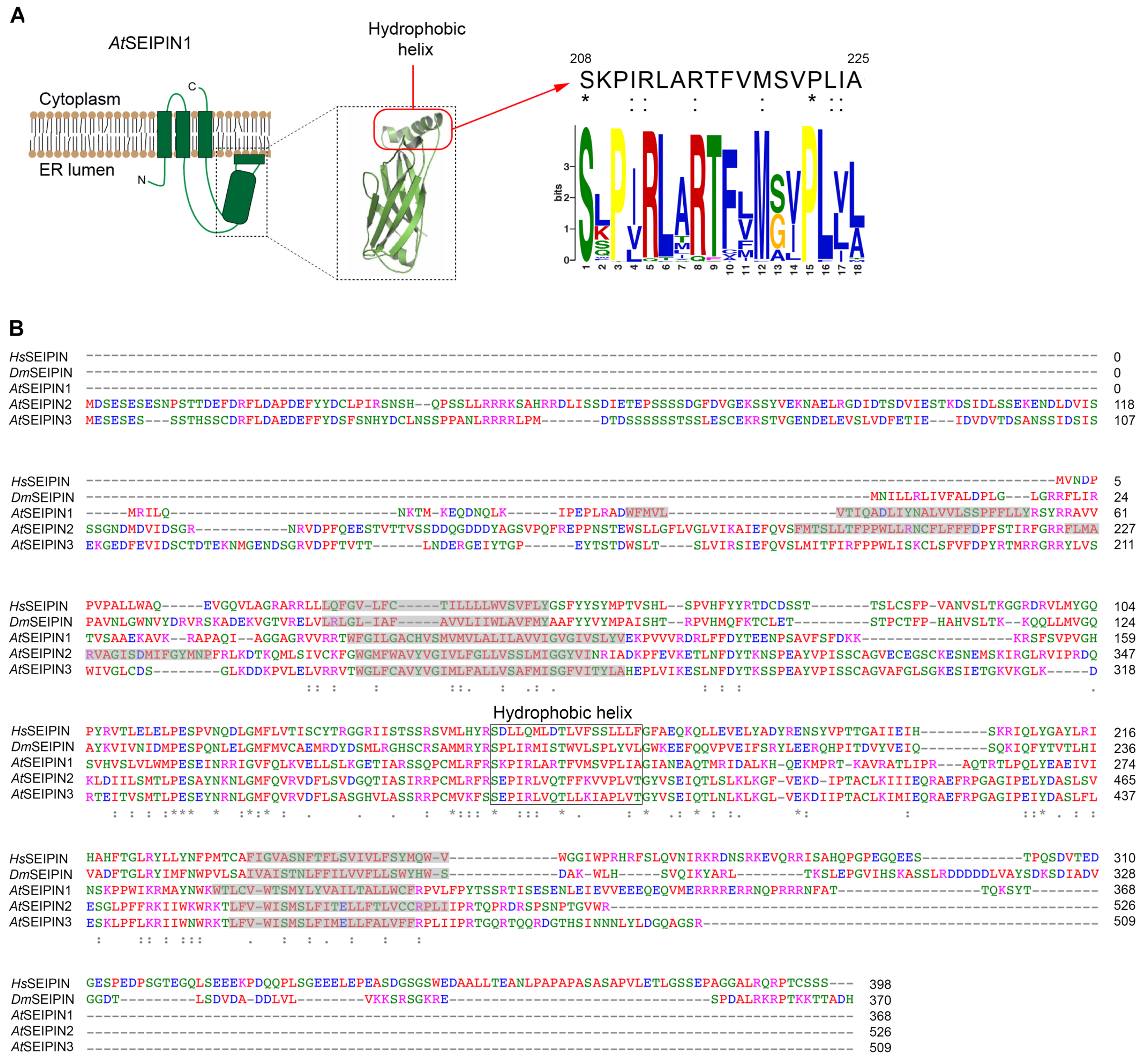

Supplemental Figure S5. Model of the Arabidopsis SEIPIN1 protein and conservation of the HH sequence in Arabidopsis, human and fly SEIPINs. Supports Figure 5, 6, 7, 8 and 9.
(A) Model of the Arabidopsis SEIPIN1 protein within the ER membrane according to putative TMDs for SEIPIN1 reported in Greer et al. (2020) and structural homology modelling of the evolutionarily conserved HH sequence on the ER lumenal side of SEIPIN1 (Chapman et al., 2019). Sequence alignments of the HH in Arabidopsis SEIPIN1 (shown, residues 208–225) and 96 other plant SEIPIN1 protein homologs revealed identical and similar amino acids indicated with asterisks and colons, respectively. Shown also is the sequence logo of the SEIPIN1 HH extracted from the motif-based sequence analysis of plant SEIPIN1 proteins using MEME. The bits scores indicate residue frequency for each position in the motif. **(B)** Deduced polypeptide sequence alignment of human, fly, and Arabidopsis SEIPIN proteins. Alignments of *H. sapiens* (Hs), *D. melanogaster* (Dm) and *A. thaliana* (At) SEIPIN1, 2, and 3 were generated using Clustal Omega. Asterisks indicate positions which have a single, fully conserved residue; colons indicate conservation between groups of strongly similar amino acids; and periods

Supplemental Figure S5. continued

indicate conservation between groups of weakly similar amino acids. Amino acid residues are highlighted as follows: red - small and mostly hydrophobic residues (A, V, F, P, M, I, L and W); blue - acidic residues (D and E); magenta - basic residues (R and K); green - hydroxyl, sulfhydryl, amine and glycine residues (S, T, Y, H, C, N, Q and G). The HHs and TMDs in SEIPIN sequences are indicated by the box and grey shading, respectively, and are based on those previously described for the human and fly proteins in Chung et al. (2019) and the Arabidopsis proteins in Greer et al. (2020).

Supplemental Data. Pyc and Gidda et al. (2021). LDIP Cooperates with SEIPIN and LDAP to Facilitate Lipid Droplet Biogenesis in Arabidopsis. Plant Cell.

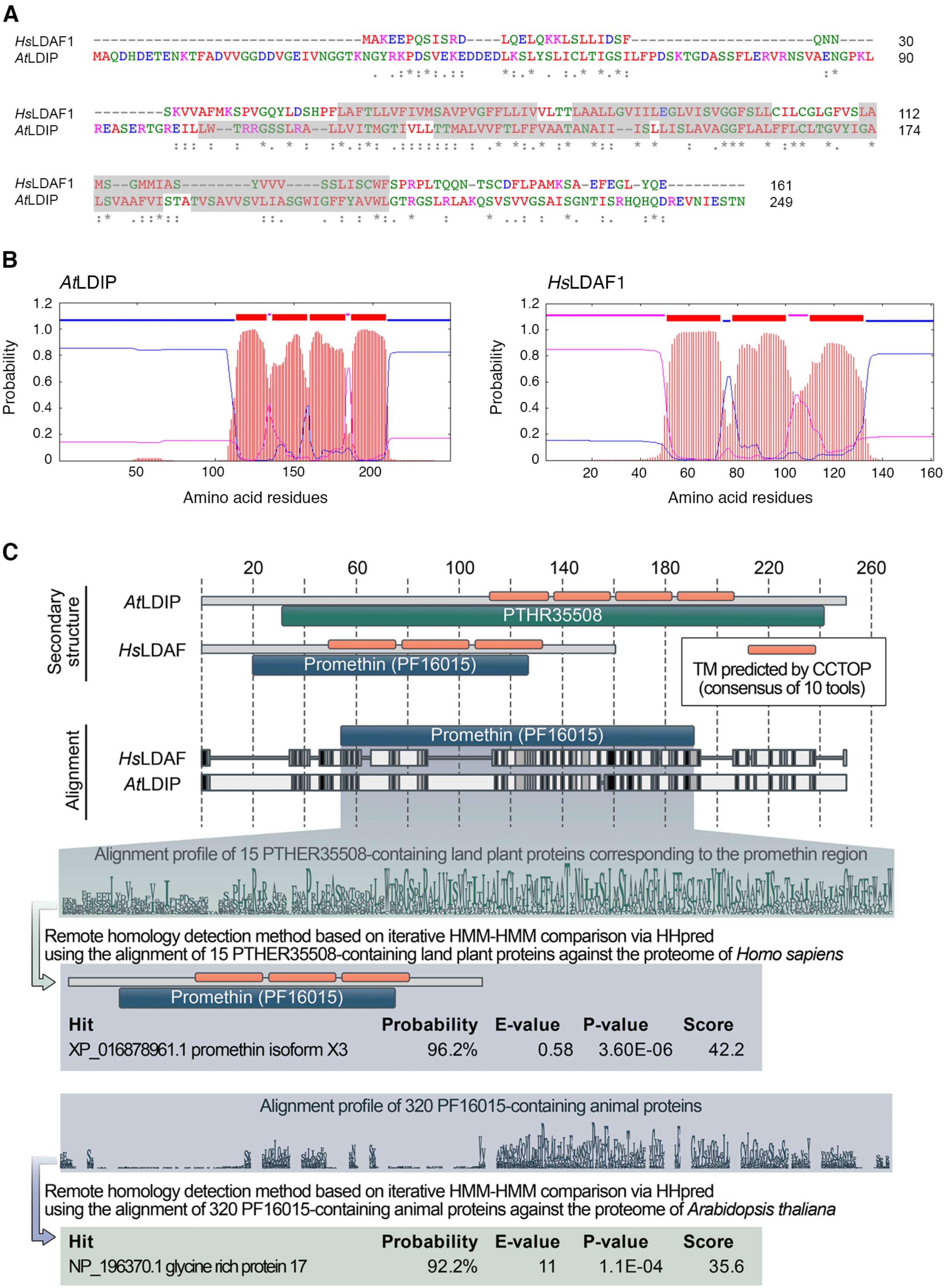

Supplemental Data. Pyc and Gidda et al. (2021). LDIP Cooperates with SEIPIN and LDAP to Facilitate Lipid Droplet Biogenesis in Arabidopsis. Plant Cell.

Supplemental Figure S6. Similarity of Arabidopsis LDIP and human LDAF1. Supports Figure 5, 6, 7, 8 and 9. **(A)** Deduced polypeptide sequence alignment of *H. sapiens* (Hs) LDAF1 and *A. thaliana* (At) LDIP generated using Clustal Omega. Asterisks indicate positions which have a single, fully conserved residue; colons indicate conservation between groups of strongly similar amino acids; and periods indicate conservation between groups of weakly similar amino acids. Amino acid residues are highlighted as follows: red - small and mostly hydrophobic residues (A, V, F, P, M, I, L and W); blue - acidic residues (D and E); magenta - basic residues (R and K); green - hydroxyl, sulfhydryl, amine and glycine residues (S, T, Y, H, C, N, Q and G). Numbers to the right of each row of sequences represent specific amino acids. TMDs are indicated by the grey shading, based on **(B)** and as previously described for human LDAF1 in Chung et al. (2019) and Arabidopsis LDIP in Pyc et al. (2017b). **(B)** Comparison of hydropathy profiles of Arabidopsis LDIP and human LDAF1 polypeptide sequences (as shown in **[A]**), based on the TMHMM server. Note that both proteins possess several contiguous hydrophobic segments, which, in LDAF1, partially overlap with the protein's promethin (PF16015) domain predicted by InterProScan (Jones et al., 2014) (refer to **[C]**). **(C)** Putative deep homology of plant LDIP and animal LDAF1 proteins. Schematic representations of the protein domain structure of Arabidopsis LDIP and *H. sapiens* LDAF1, showing their PTHER35508 domain and promethin (PF16015) domains, respectively, based on the UniProt/InterPro database. Putative TMDs in each protein are also shown, based on the CCTOP prediction server; refer also to **(B)**. Numbers at the top represent the relative positions of specific amino acids in LDIP and LDAF1. To account for sequence variation in PTHER35508 domain-containing proteins of embryophytes, a phylodiverse data set of 15 PTHR35508-containing sequences from across the land plant tree of life was obtained from UniProt/InterPro, including Arabidopsis LDIP, as well as protein homologs from other selected plant genomes. Using MAFFT, an alignment was then generated of the conserved region in the land plant proteins that corresponds to the region that can be aligned with the promethin (PF16015) domain in the human LDAF1 protein sequence; refer to sequence logo in **(C)** corresponding to amino acid residues in alignment (refer also to Supplemental Dataset S6 for the list of all the sequences used for alignment). This alignment was then used for a pairwise comparison of profile hidden Markov models search via HHpred against the proteome of *H. sapiens*. As shown, the top hit recovered was LDAF1 (annotated at HHpred as promethin isoform X3). Similarly, an MAFFT-based alignment was generated of a phylodiverse set of 320 animal PF16015-containing protein sequences (i.e., putative LDAF1 homologs) obtained from UniProt/InterPro (refer to Supplemental Dataset S7 for the list of all sequences) and used for a pairwise comparison of profile hidden Markov models search via HHpred against the *A. thaliana* proteome. As shown, the top hit recovered, albeit at relatively low confidence scores, was the glycine-rich protein 17, which is also annotated at UniProt and described in Huang (2018) as anther tapetum-specific oleosin (AT5G07530). A list of all proteins recovered in the plant LDIP and animal LDAF1 HHpred-based searches are shown in Supplemental Dataset S8 and S9, respectively.

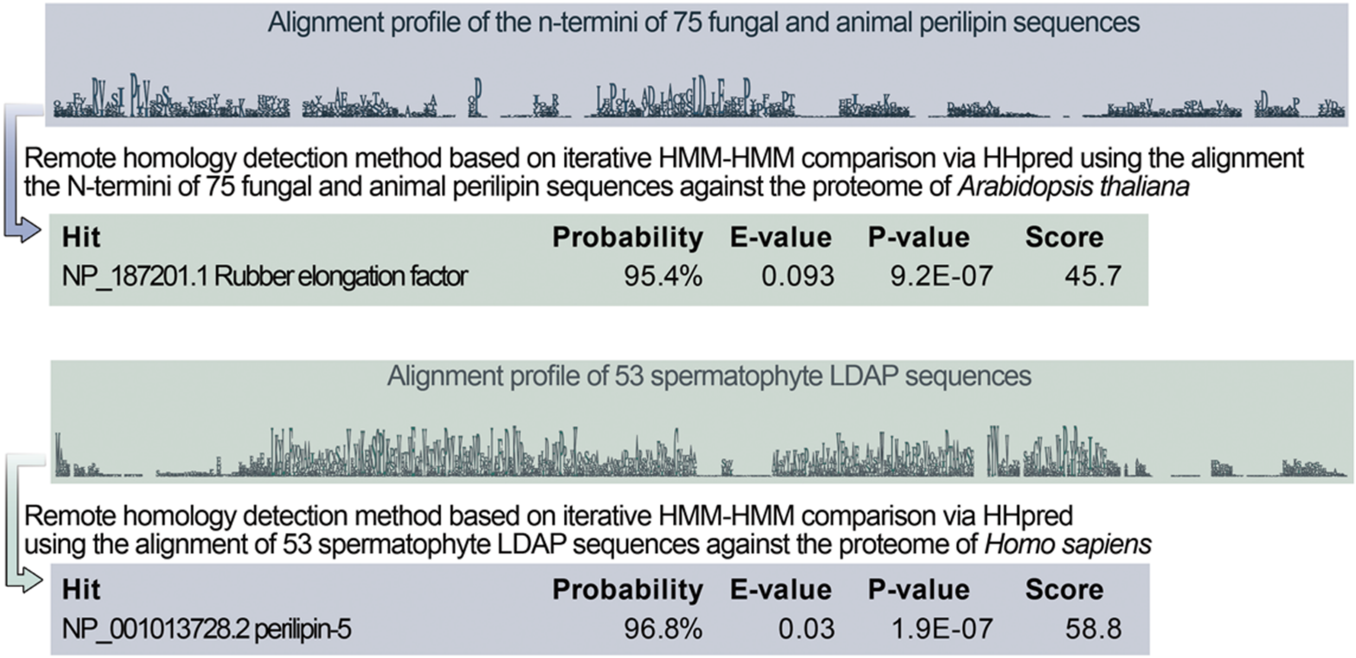

Supplemental Figure S7. Deep homology of plant LDAP and animal PLIN proteins. Supports Figure 9. Sequences of 75 curated animal and fungal PLIN proteins were obtained from UniProt/InterPro and their N-terminal sequences (i.e., the region corresponding to amino acids 22–161 of *H. sapiens* PLIN3) were aligned using MAFFT; refer to Supplemental Dataset S10 for the list of all sequences. The alignment was then used for a pairwise comparison of profile hidden Markov models search (via HHpred) against the *A. thaliana* proteome. As shown, the top hit recovered was LDAP3 (annotated at HHpred as rubber elongation factor). Note that subsequent top hits included LDAP1 and LDAP2; refer to Supplemental Dataset S11 for a list of all plant proteins recovered in the HHpred search using animal and fungal PLIN proteins. Similarly, an MAFFT-based alignment was generated of 53 angiosperm and gymnosperm LDAP homologs obtained from UniProt/InterPro (refer to Supplemental Dataset S12 for the list of all the sequences used for alignment) and used for a pairwise comparison of profile hidden Markov models search via HHpred against the *H. sapiens* proteome. As shown, the top hit recovered was PLIN5. Subsequent top hits included PLIN isoforms 1–4; refer to Supplemental Dataset S13 for a list of all human proteins recovered in the HHpred search using plant LDAPs.

**Supplemental Table S1.** Metadata file for LC-MS/MS processing of *WT* and *ldip* mutant proteomics with MaxQuant.	
**1. General features**	
Responsible persons	Dr. Till Ischebeck[1], Dr. O. Valerius[2], Prof. G.H. Braus[2]<br>[1]Department of Plant Biochemistry, Albrecht-von-Haller-Institute for Plant Sciences, University of Göttingen, Justus-von-Liebig-Weg 11, 37077 Göttingen, Germany<br>[2]Department of Molecular Microbiology and Genetics, Institute for Microbiology and Genetics, University of Göttingen, Grisebachstr.8, 37077 Göttingen, Germany
Instrument manufacturer, model	Thermo Fisher Scientific, Orbitrap Velos Pro
Experimental design	Analysis of total, and lipid droplet (LD)-enriched proteome of Arabidopsis wild-type (*WT*), *ldip* knockdown (KD) and *ldip* knockout (KO) 40-h-old, germinated seedlings
Groups	Total protein, LD-enriched fraction
Biological and technical replicates	3 biological replicates for each fraction and genetic background<br>Technical replicates (independent LC-MS runs): 1 for each sample<br>FK_V_146-148: biological replicates of total protein, *WT*<br>FK_V_149-151: biological replicates of total protein, *ldip* KO<br>FK_V_152-154: biological replicates of total protein, *ldip* KD<br>FK_V_155-157: biological replicates of LD-enriched fraction, *WT*<br>FK_V_158-160: biological replicates of LD-enriched fraction, *ldip* KO<br>FK_V_161-163: biological replicates of LD-enriched fraction, *ldip* KD
Sample amount	18
**2. Electrospray Ionisation (ESI)**	
Supply type (static, or fed)	fed
Interface manufacturer, model	Thermo Fisher Scientific
Sprayer type, manufacturer, model	Nano Spray Flex Ion Source
**3.1 Post source component Analyser**	
Ion optics, 'simple' quadrupole, hexapole, Paul trap, linear trap, magnetic sector FT-ICR, Orbitrap : name of the analyser(s)	Linear ion trap<br>Orbitrap
**3.2 Post-source component — 3.2 Activation / dissociation**	
Instrument component where	Velos Pro linear ion trap

the activation / dissociation occurs	
Gas type (when used)	Helium
Activation / dissociation type	CID
**4. Spectrum and peak list generation and annotation — 4.1 Data acquisition**	
Software name and version	Xcalibur 2.2
Acquisition parameters	Data-dependent Top10
Software name and version	MaxQuant 1.5.8.3
**4. Spectrum and peak list generation and annotation — 4.2 Resulting data**	
Location of source (‘raw’) and processed files	The mass spectrometry proteomics data have been deposited to the ProteomeXchange Consortium via the PRIDE partner repository with the dataset identifier (PXD012992).
**5. Description of the software and methods applied in the quantitative analysis (including transformation functions, aggregation functions and statistical calculations).**	
Quantification software name, version and manufacturer	MaxQuant Version 1.5.8.3
Description of the selection and/or matching method of features, together with the description of the method of the primary extracted quantification values determination for each feature and/or peptide	Upload of all .raw files into the software. Grouping of technical replicates as one Experiment (“set experiment”).<br>Group-specific parameters:<br>1) Type: default<br>2) Digestion: default<br>3) Modifications: Variable Modifications: default + GlyGly (K) + Phospho (STY)<br>4) Label-free quantification: LFQ, default<br>5) Instrument: intensity determination: total sum, rest default<br>6) First search: default<br>7) Misc: default<br>Global parameters<br>1) Sequences: updated TAIR10 peptides from 14.12.2010, rest default<br>2) Identification: PSM FDR=0.02, protein FDR=0.02, rest default<br>3) Adv. Identification: match between runs ✓, dependent peptides ✓<br>4) Protein quantification: default<br>5) Label free quantification: iBAQ ✓<br>6) Tables: default<br>7) MS/MS – FTMS: FTMS recalibration ✓, rest default<br>8) MS/MS – ITMS: default<br>9) MS/MS – TOF: default<br>10) MS/MS – unknown: default<br>Advanced: default

Confidence filter of features or peptides prior to quantification	Global parameters: identification: PSM FDR=0.02, protein FDR=0.02, rest default
Normalization	All values were divided by the total iBAQ intensities or total LFQ intensities in one sample and multiplied by 1000.

**Supplemental Table S2.** Access information to raw MS data for GFP-SEIPIN1 and GFP-LDIP (with or without co-expressed Arabidopsis LEC2) affinity-capture experiments.
In order to access the raw MS (proteomics) data generated for this study (Pyc et al.) and Pyc et al. (2017b) from the ProteomeXchange Consortium via the PRIDE database, a username and password is required.<br><br>The data can be accessed at https://www.ebi.ac.uk/pride/archive/login using the username and password provided below.<br><br>Pending the acceptance of the manuscript, the data will be accessible to the public.
**Dataset identifier:** PXD023043<br>**Username (Email):** reviewer_pxd023043@ebi.ac.uk<br>**Password:** MWZylzJb

Supplemental Table S3. Names and sequences of oligonucleotide primers used in this study.

Plasmid Construction

Shown is the name of each plasmid newly constructed in this study (in bold), along with the corresponding template plasmid DNA(s) or cDNA (in parentheses) and the names and sequences (5'-3') of the forward (Fp) and reverse (Rp) primers used for cloning. Plasmids listed without primers were generated using Gateway cloning technology; plasmids listed with two template plasmid DNAs and three sets of primer pairs were generated using overlap extension PCR-based cloning. See 'Plasmid Construction' section in the Materials and Methods for additional details on cloning procedures.

pDONR/LDIP (pRTL2/Cherry-LDIP [Pyc et al., 2017])
LDIP Fp GGGGACAAGTTTGTACAAAAAAGCAGGCTTAACCATGGCGCAAGATCACGACGAG
LDIP Rp GGGGACCACTTTGTACAAGAAAGCTGGGTCTCAGTTGGTTGATTCGATGTTTAC

pMDC32/LDIP (pDONR/LDIP [This study])

pDONR/SEIPIN2∆HH (pDONR/SEIPIN2 [Greer et al., 2020])
SEIPIN2∆HH Fp CGTCCGTGCATGTTAAGATTCTCTGAGATCCAAACCTTGAGC
SEIPIN2∆HH Rp GCTCAAGGTTTGGATCTCAGAGAATCTTAACATGCACGGACG

pMDC43/GFP-SEIPIN2∆HH (pDONR/SEIPIN2∆HH [This study])

pK83/LDIP (pRS316-PGK/LDIP [This study])
NcoI-LDIP Fp CATGCCATGGCGCAAGATCACGAC
LDIP-SacII Rp CATGCCGCGGTCAGTTGGTTGATTCGATGTTTACC

pB19/LDIP (pK83/LDIP [This study])

pDONR/NbLDIP RNAi (4-week-old *N. benthamiana* leaf cDNA)
pDNR-NbLDIP-RNAi Fp GGGGACAAGTTTGTACAAAAAAGCAGGCTTCACTAAGGAAGATTCAACAAA
pDNR-NbLDIP-RNAi Rp GGGGACCACTTTGTACAAGAAAGCTGGGTCGTCCCCACAGAAACAACAAG

pB7GW1WG2/NbLDIP RNAi (pDONER/NbLDIP RNAi [This study])

pDONR/EMP1(4-week-old Arabidopsis leaf cDNA)
EMP1 Fp GGGGACAAGTTTGTACAAAAAAGCAGGCTTCATGGCTATGGAGTTTCTGAG
EMP1 Rp GGGGACCACTTTGTACAAGAAAGCTGGGTCATCGATCTTAACAGAGGAATAG

pMDC32/EMP1-Cherry (pDONR/EMP1 [This study])

pDEST-SCYCE/cCFP-LDIP (pDONR/LDIP [This study])

pDEST-VYNE/nVenus-SEIPIN2∆HH (pDONR/SEIPIN2∆HH [This study])

pIB/Venus-LDIP (pIB/Venus [Hull et al., 2009] and pGAD/LDIP [Pyc et al., 2017])
Venus-start Fp ATGGTGAGCAAGGGC
Venus-LDIP Rp GTGATCTTGCGCCATGGCGGCGGTCACGCG

Venus-LDIP Fp CGCGTGACCGCCGCCATGGCGCAAGATCAC
LDIP-Rp GTTGGTTGATTCGATGTTTACC

Supplemental Table S3 continued

Venus-start Fp (See above)
LDIP Rp (See above)

pIB/Cherry-LDIP (pIB/Cherry [Fabrick and Hull, 2017] and pIB/Venus-LDIP [This study])
Cherry Fp ATGGTGAGCAAGGGCGAG
Cherry-LDIP Rp GTGATCTTGCGCCATCTTGTACAGCTCGTC

Cherry-LDIP Fp GACGAGCTGTACAAGATGGCGCAAGATCAC
LDIP Rp (See above

Cherry Fp (See above)
LDIP Rp (See above)

pIB/LDAP3-Venus (pGAD/LDAP3 [Pyc et al., 2017] and pIB/Venus [Hull et al., 2009])
LDAP3-start Fp ATGGCTACTCAAACGGATCT
LDAP3-Venus Rp CTCGCCCTTGCTCACCATATCAAGTGGATGGAA

LDAP3-Venus Fp TTCCATCCACTTGATATGGTGAGCAAGGGCGAG
Venus Rp TTAGGCGGCGGTCACGCG

LDAP3-start Fp (See above)
Venus Rp (See above)

pIB/LDAP3 (pIB/LDAP3-Venus [This study])
LDAP3 Fp (See above)
LDAP3 Rp ATCAAGTGGATGGAACTCCA

pIB/LDAP3ΔC100 (pIB/LDAP3 [This study])
LDAP3 Fp (See above)
LDAP3ΔC100 Rp GTAGACGGATTTAGCCATTCC

pGAD/SEIPIN2 (pMDC43/SEIPIN2 [Cai et al., 2015])
BamHI-SPN2 Fp GGGCCCGGATCCCAATGGACTCCGAGTCCGAGTCC
SacI-SPN2 Rp CGAGAGCTCCTCTACCTCCAGACTCCAGTTGGG

pGAD/SEIPIN2ΔHH (pDONR/SEIPIN2ΔHH [This study])
BamHI-SPN2 Fp (See above)
SacI-SPN2 Rp (See above)

pGAD/SEIPIN2 HH (pGADT7 [Clontech])
pGAD-SPN2 HH Fp CTTCAAAGTGGTTCCGTTAGTGACAGGATATGTCGGGCATCGATACGGGATC
pGAD-SPN2 HH Rp AATGTCTGAACAAGACGAATAGGCTCACTTCTGAAACCCGGGTGGAATTCACT

pGAD/GFP-SEIPIN2 HH (pGAD/SEIPIN2 HH [This study])
*Eco*RI-GFP Fp GCGAATTCATGAGTAAAGGAGAAGAAC
*Xma*I-GFP-Rp GACCCGGGTATTTGTATAGTTCATCCATGCC

pRS316-PGK/LDIP (pMDC32/LDIP [This study])
AtLDIP Fp CATCTGGATCCATGGCGCAAGATCACGAC
AtLDIP Rp CCATCGAATTCTCAGTTGGTTGATTCGATGTTTAC

Supplemental Table S3 continued

pRS313-PGK/LDAP3 (pIB/LDAP3 [This study])
AtLDAP3 Fp CATCTGGATCCATGGCTACTCAAACGGATC
AtLDAP3 Rp CCATCGAATTCTCAATCAAGTGGATGGAAC

RT-PCR

Shown is the name of the target gene transcript (and species) and the names and sequences (5'-3') of the forward (Fp) and reverse (Rp) primers used for RT-PCRs. See 'RT-PCR and RT-qPCR' section in the Materials and Methods for additional details.

Arabidopsis *LDIP*
AtLDIP Fp CACGACGAGACGGAGAATAAAACCTTT
AtLDIP Rp CCCAATCCAACCTGAAGCTATTAAGAC

Arabidopsis *LDAP1*
AtLDAP1 Fp CCGGCCGCTAGCATGGAGACAGAGAAGAAAAATAG
AtLDAP1 Rp CCGGCCGCTAGCCTCCGAATCAGACGATGATTTA

Arabidopsis *LDAP3*
AtLDAP3 Fp CCGGCCCCATGGCTACTCAAACGGATCTC
AtLDAP3 Rp CCGGCCCCCGGGATCAAGTGGATGGAACTCC

Arabidopsis *TUBULIN*
AtTUBULIN Fp CGATGTTGTGAGGAAGGAAG
AtTUBULIN Rp GACACCACTCATTGTAGCAG

Arabidopsis *SEIPIN1*
AtSEI1 Fp CAAAATCCCTGAACCATTACG
AtSEI1 Rp GTGGAACACTCATCACAAATG

Arabidopsis *LEC2*
AtLEC2 Fp CGTAAGCGTGATCCTCAGG
AtLEC2 Rp AGAAAGAACGCCATGATGAG

***M. musculus FIT2* in Arabidopsis**
MmFIT2 Fp AGAAAGAACGCCATGATGAG
MmFIT2 Rp CCGGCCGGATCCATGGAGCACCTGGAGC

Arabidopsis *SEIPIN2* in *N. benthamiana*
AtSEI Fp ATGGACTCCGAGTCCGAG
AtSEI2 Rp CTACCTCCAGACTCCAGT

N. benthamiana ACTIN
NbACTIN Fp GATGAAGATACTCACAGAAAGA
NbACTIN Rp GTGGTTTCATGAATGCCAGCA

T. ni ACTIN
TnACTIN Fp ACAGAAGGACTCGTACGTAG
TnACTIN Rp TTGATGTCGCGCACGATCTC

Supplemental Table S3 continued

Arabidopsis *LDAP3* in *T. ni*
AtLDAP3 Fp ATGGCTACTCAAACGGATCTCG
AtLDAP3 Rp TCAATCAAGTGGATGGAACTCCAAAG

Arabidopsis *LDAP3ΔC100* in *T. ni*
AtLDAP3ΔC100 Fp TGGCTACTCAAACGGATCTCG
AtLDAP3ΔC100 Rp TCAACGGTCAAGCTCAGTCACAG

RT-qPCR

Shown is the name of the species and target gene transcript and the names and sequences (5'-3') of the forward (Fp) and reverse (Rp) primers used for qPCRs. See 'RT-PCR and RT-qPCR' section in the Materials and Methods for additional details.

N. benthamiana LDIP
NbLDIP Fp TTCTTTGCCTGCCTAACAGC
NbLDIP Rp TTGTTACCAGCCATATTGCCC

N. benthamiana L23
NbL23 Fp AAGGATGCCGTGAAGAAGATGT
NbL23 Rp GCATCGTAGTCAGGAGTCAACC

Abbreviations: At, *Arabidopsis thaliana*; FIT2, Fat Storage-Inducing Transmembrane protein 2; LEC2, LEAFY COTYLEDON 2; L23, 60S ribosomal protein; LDAP1/LDAP3, Lipid Droplet (LD)-Associated Protein 1 and 3; LDAP3ΔC100, C-terminal 100-amino-acid truncation mutant of LDAP3; LDIP, LDAP-Interacting Protein; Mm, *Mus musculus*, Nb, *Nicotiana benthamiana*, SEI1/2, SEIPIN1/2; Tn, *Trichoplusia ni*.
