# Supplementary material for: LDIP cooperates with SEIPIN and LDAP to facilitate lipid droplet biogenesis in Arabidopsis
Source: Plant Cell. 2021 Jun 9;33(9):3076–103. doi: 10.1093/plcell/koab179 (PMC8462815; doi:10.1093/plcell/koab179)
Supplement: koab179_Supplementary_Data [file koab179_supplementary_data.zip › tpc.00378.2021-s14.pdf]

LDIP Cooperates with SEIPIN and LDAP to Facilitate Lipid Droplet Biogenesis in Arabidopsis

Michal Pyc, Satinder K. Gidda, Damien Seay, Nicolas Esnay, Franziska K. Kretzschmar, Yingqi Cai, Nathan M. Doner, Michael S. Greer, J. Joe Hull, Denis Coulon, Claire Bréhélin, Olga Yurchenko, Jan de Vries, Oliver Valerius,Gerhard H. Braus, Till Ischebeck, Kent D. Chapman, John M. Dyer, Robert T. Mullen

Corresponding author: John M. Dyer (john.dyer@usda.gov) Robert T. Mullen (rtmullen@uoguelph.ca).

Review timeline:

**TPC2020-RA-00396**	Submission received:	May 19, 2020
	1st Decision:	July 9, 2020 *manuscript declined (might reconsider)*
**TPC2021-RA-00378**	Submission received:	April 30, 2021
	1st Decision:	June 2, 2021 *accept with minor revisions (no further review)*
**TPC2021-RA-00378R1**	1st Revision received:	June 2, 2021
	2nd Decision:	June 9, 2021 *acceptance pending, sent to science editor*
	Final acceptance:	June 26, 2021

REPORT: (The report shows the major requests for revision and author responses. Minor comments for revision and miscellaneous correspondence are not included. The original format may not be reflected in this compilation, but the reviewer comments and author responses are not edited, except to correct minor typographical or spelling errors that could be a source of ambiguity.)

TPC2020-RA-00396 1st Editorial decision – *declined* July 9, 2020

Thank you for choosing to send your manuscript entitled "Protein Cooperation Facilitates Lipid Droplet Biogenesis in Plants" for consideration at The Plant Cell. Your submission has been evaluated by members of the editorial board as well as expert reviewers in your field, and we regret to inform you that we are not able to recommend publication of this manuscript in its current form. We have not made this decision lightly. We have had input from multiple scientists, and we have solicited post-review comments as well. Our present policy is to offer streamlined decisions and to not advise on the direction of the work by requesting extensive modifications or substantial additional experiments.

That said, we would be willing to re-consider a new manuscript that fully addresses the concerns raised during this review process. If you decide to resubmit to The Plant Cell, it will be evaluated as a new submission subject to full assessment by the editorial board, and if sent for external review, a new set of reviewers is likely to be chosen. Reviewers will be asked to assess your work as a new manuscript (i.e. are the claims fully supported by the data and do the results presented move the field forward?), and not only whether previous reviewer comments have been addressed.

We generally feel that although this current manuscript provides insights on a revised model of LD biogenesis, the main claims of the model, i.e. 1) SEPIN-LDPI interaction, 2) LDAP recruitment to forming LD by LDIP, 3) that the ratio of SEPIN1:LDIP governs LD size and number, and 4) evolutionary conservation of AtLDAPs and mammalian promethin/LDAF1, are not, or are not sufficiently supported by the data. The support for our opinion is detailed in the comments by the three reviewers.

It will be important to convince the editors and reviewers that the study adds significant new understanding(s) of mechanisms or processes, or otherwise represents a significant advance in the field, and that the major claims made are fully justified by the data presented. This includes careful consideration and explanation of the various controls used in experiments, the extent and manner of replication, and the statistical analyses used. Sampling methods and the nature of "biological replicates" should be described precisely (i.e. different plants, parts of plants, pooled tissue, independent pools of tissue, sampled at different times, etc.), along with a clear description of and rationale for any statistical analyses conducted. The reader should know exactly what was sampled; what forms the basis of the calculation of any means and other statistical variables and parameters reported. This is also necessary to ensure

that proper statistical analysis was conducted. -- Reviewer comments:

TPC2021-RA-00378 Submission received April 30, 2021

Reviewer comments on previously declined manuscript and **author responses:**

Reviewer #1:

Lipid droplets (LDs) serve as an important form of energy storage in plant cells, particularly in seeds. LDs form from the ER and their cell biology is unique because they are surrounded by a lipid monolayer, rather than the bilayer that surrounds other organelles. A number of proteins are required for LD biogenesis and stabilization. In seeds, oleosin proteins are critical to LD formation, but other proteins are also hypothesized to play a role as well. Previously, the authors have identified several proteins that participate in LD formation and/or stabilization. Here, they investigate how these proteins interact, drawing on recent literature from mammalian LD formation to guide their hypotheses. They find that, similar to mammalian LD formation, a complex set of interactions takes place to recruit LD associated proteins to the forming LD and to govern final LD size. The authors present a revised model for lipid droplet biogenesis in Figure 8, which is quite interesting. The main claims of this model are: 1) SEPIN-LDPI interaction, 2) LDAP recruitment to forming LD by LDIP, 3) that the ratio of SEPIN1:LDIP governs LD size and number, and 4) evolutionary conservation of AtLDAPs and mammalian promethin/LDAF1. The discussionis well-written and integrates some key ideas from a wide variety of sources to generate an interesting model. However, in several cases the data presented to support this model are either presented incompletely or lack appropriate controls, which makes the authors' claims difficult to evaluate.

Point 1. Their first main finding, that SEPINs interact with LDPIs is not supported by the data that are currently presented (the proteomics) or are supported by data without appropriate control experiments (the BiFC).

> **RESPONSE: We now provide five lines of evidence supporting an interaction between SEIPIN and LDIP, including: 1) Reciprocal affinity-capture experiments using either GFP-SEIPIN1 or GFP-LDIP as bait (Figure 4A and Supplemental Dataset 5; and Pyc et al. 2017b); 2) Dynamic relocalization of LDIP from LDs to ER when ER-localized SEIPIN is over-expressed (Figure 4B); 3) Loss of relocalization of LDIP to the ER when a hydrophobic helix known to be critical for interaction of human SEIPIN and LDAF1 was deleted from SEIPIN2 (SEIPIN2HH) (Figure 5B); 4) BiFC experiments showing a positive interaction between LDIP and SEIPIN2, but not between LDIP and SEIPIN2HH (Figure 5C); and 5) Yeast 2-hybrid experiments, showing a positive interaction between LDIP and SEIPIN2, as well as LDIP and a sufficiency construct containing just the hydrophobic helix sequence from SEIPIN2 alone, but not between LDIP and SEIPIN2HH (Figure 5E).**

Point 2. The iBAQ proteomics methods are sufficiently documented, the results have been deposited in PRIDE, and the results presented in detail in the supplemental datasets, but these essential details are lacking for the Affinity-Capture of GFP-SEIPIN1. The proteomics data from Affinity-Capture of GFP-SEIPIN1 must be deposited in a proteomics database (e.g. PRIDE) and/or presented in full here (as noted in the Plant Cell Instructions for Authors), otherwise it is impossible to evaluate these experiments and the conclusions that the authors draw from them.

> **RESPONSE: As now stated in the Methods of the revised manuscript (see 'Affinity-Capture of GFP-SEIPIN1 Expressed in N. benthamiana Leaves' section), all of the data from the affinity-capture experiments with GFP-SEIPIN1, as well as from affinity-capture experiments with GFP-LDIP, as previously described in Pyc et al. (2017b), are shown in Supplemental Dataset 5. All of the data are available also through the ProteomeXchange Consortium via the PRIDE partner repository (https://www.ebi.ac.uk/pride/), under the project accession number PXD023043. In addition, we have provided details on accessing the data for both the GFP-SEIPIN1 and GFP-LDIP affinity-capture experiments in Supplemental Table 3.**

Point 3. Similarly, the BiFC experiment replaces LDIP-cCFP with untagged cCFP as a negative control for interaction with nVenus-SEPIN1. As discussed in many papers (e.g. Kudla & Bock 2016, Plant Cell) and clearly indicated in the Plant Cell Instructions for Authors, this is an insufficient negative control for BiFC. These experiments must be

performed with appropriate negative controls (i.e. proteins with the same subcellular localization) in order for the authors to draw meaningful and robust conclusions from them.

RESPONSE: As suggested, we have provided new BiFC data in the revised manuscript (see Figure 5C), showing that co-expression of cCFP-LDIP and nVenus-SEIPIN2 yielded a BiFC fluorescence signal in N. benthamiana leaf cells, but there was significantly less BiFC fluorescence when cCFP-LDIP was co-expressed with nVenus-SEIPIN2ΔHH, which consists of SEIPIN2 without its conserved hydrophobic helix (HH) sequence (amino acids 395-416 [refer to Supplemental Figure 4]). Notably, we showed also that GFP-SEIPIN2ΔHH is properly localized to the ER, consistent with the localization of (full-length) GFP- SEIPIN2 (Figure 5A) Confirmation of expression of both pairs of BiFC fusion constructs using RT-PCR is provided in Supplemental Figure 2F.

Point 4. Also, if SEPINs recruit LDIPs to the ER, as the authors document using heterologous overexpression in Fig 4C, why is the BiFC signal for these two proteins not also localized to the ER? Do these puncta colocalize with LDs? Please repeat the experiments with appropriate controls and counterstaining for neutral lipids.

RESPONSE: As now shown in the revised manuscript (see Figure 5D), close analysis (via CLSM) of the BiFC fluorescence signal attributable to cCFP-LDIP and nVenus-SEIPIN2 in N. benthamiana leaf cells revealed an aggregated and reticular-like structure(s) that was in close association with MDH-stained LDs and resembles the reorganization of the ER in cells overexpressing SEIPIN proteins and their localization to ER-LD junctions; compare images in Figure 5D with those in Supplemental Figure 4, as well as those presented previously in Cai et al. (2015), Taurino et al. (2018) and Greer et al. (2020).

Point 5. Their second main finding, that LDIP recruits LDAP to developing LDs is supported by data with inappropriate control experiments. For the LDIP RNAi, the authors attempt to evaluate the efficacy of this RNAi construct using RT-PCR (Fig S4D), which is insufficient to document a quantitative change in transcript levels and indeed, there seems to still be quite a lot of LDIP transcript in these samples. qPCR is necessary (as noted in the Plant Cell Instructions for Authors) to quantitatively evaluate whether the RNAi construct is functioning as the authors claim.

RESPONSE: In support of the experiments involving LDIP RNAi presented in Figure 3C, both RT-PCR and (new) qPCR analysis confirming the suppression of endogenous LDIP expression in N. benthamiana leaves are presented in Supplemental Figure 2D and 2E, respectively. Statistical analysis of the qPCR results is shown in Supplemental Dataset 1. It is also worthwhile mentioning that, while our qPCR results indicate that LDIP transcripts are still detectable in LDIP RNAi-infiltrated leaf cells (Supplemental Figure 2E), the consistent appearance of the aberrant, supersized LDs in these cells (Figure 3B) resembles the same enlarged LD phenotype observed in leaves of an Arabidopsis ldip knockdown (and knockout) transgenic line (Pyc et al., 2017b).

Point 6. Their third claim, that the ratio of SEPIN1:LDIP governs LD size and number suffers from the same issue, since they use RT-PCR, not qPCR, to evaluate SEPIN1 and LDIP overexpression levels. Western blots would be the ideal way to evaluate expression levels, but qPCR would be acceptable. "semi-quantitative" RT-PCR is insufficient to document quantitative changes in transcript levels (as noted in the Plant Cell Instructions for Authors)..

RESPONSE: We have revised Figure 7 to more accurately depict LD numbers and sizes for the various lines and experimental conditions tested. Notably, these experiments employed T1 transgenic lines that were generated by stably transforming WT and well-established homozygous ldip KO (Pyc et al., 2017b) and SEIPIN1 overexpression lines (Cai et al., 2015) with LDIP or SEIPIN1, and included parallel transformations with the empty expression vector serving as controls. T1 progeny were subsequently selected first based on antibiotic resistance to confirm the presence of the new transgene, then analyzed by RT-PCR to confirm the relative overexpression or absence of (trans/endogenous) gene expression (Supplemental Figure 2H). T1s were not advanced further, since for several lines it was not possible to derive full homozygosity. Rather than repeat these experiments, we have softened the language in the conclusionary statement for this section in the Results to read “Taken together, these data suggest that LDIP interacts functionally with SEIPINs to determine the number of LDs in plant cells and, furthermore, that LDIP might work together with SEIPIN to regulate LD size.” Importantly, we then follow this section up with the expression of SEIPIN1 and LDIP in a yeast SEIPIN-mutant line and show that SEIPIN1 and LDIP together, and not on their own, are both required for production of normal numbers and sizes of LDs in the heterologous system (see Figure 8 and allied text).

Point 7. Their final claim that LDAPs are evolutionarily conserved between mammals and plants seems only to be supported by a one-way homology search (Fig S3). Having Arabidopsis proteins turn up in a search using a mammalian protein as query (Fig S3) is not sufficient evidence for evolutionary conservation. At the very least, rigorous molecular phylogenetic analyses will be required to support this claim (as noted in the Plant Cell Instructions for Authors). Ideally, cross-complementation experiments would also be performed, though I recognize that the authors could feel that these experiments might be outside the scope of this study. Furthermore, the authors will need to discuss why yeast LD formation requires only yeast SEPIN, but both AtSEPIN1 and AtLDIPs, but not AtLDAPs (Fig 7). Otherwise, claims of evolutionary conservation should be removed from the manuscript.

RESPONSE: We added an evolutionary plant biologist (Dr. Jan de Vries (University of Göttingen) to the paper to provide a more detailed analysis of the homology between the various proteins in our study. In terms of the relationship between LDAPs in plants and perilipins in mammals, Dr. de Vries performed curated animal and fungal PLIN proteins, as well as angiosperm and gymnosperm LDAPs, and then used resulting conserved-sequence alignments for pairwise comparisons of profile hidden Markov models searches (via HHpred) against the Arabidopsis and human proteomes. As shown, the top hit recovered was LDAP3 (annotated at HHpred as rubber elongation factor). The results showed that LDAPs shared significant homology with the N-terminal regional of mammalian and fungal perilipin proteins. These results are presented in new Supplemental Figure 7 and described on pages 24 and 25 in the Discussion of the revised manuscript. Evolutionary relationships between plant LDIP and mammalian LDAF1 proteins were also analyzed. Unfortunately, given their relatively short polypeptide sequences, it was not possible to build robust phylogenetic trees. However, comparison of LDIP sequence alignments from plants to the animal (human) proteome (via a pairwise comparison of profile hidden Markov model search using HHpred) revealed deep homology between LDIP and LDAF1, while a reciprocal comparison of animal LDAF1 alignments to the Arabidopsis proteome identified an oleosin as a top hit. These results are presented in a new Supplemental Figure 6 and described on pages 23 and 24 in the Discussion of the revised manuscript. Additional (new) text has also been added on pages 25 and 26 in the Discussion to more thoroughly describe the proteins required for SEIPIN function in yeast. Like in plants and animals, yeast SEIPIN activity requires two proteins, one called Fld1, which has significant homology with SEIPIN proteins from other organisms, including plants and animals, and a second protein called Ldb16, which has no obvious homology with plant or mammalian proteins, including LDIP/LDAF1. Text describing these proteins, as well as several other proteins in yeast that might functionally substitute for LDIP or LDAF1 (i.e., Ldo16 and Ldo45) are also described. Whether any of these yeast proteins function in a manner similar to LDIP/LDAF1 remains to be determined.

Point 8. Currently, the microscopy experiments are mostly descriptive. Quantification could be performed on LD size in Figure 3 and should be performed on the colocalization experiments Figures 1 & 2. I suggest an object-based colocalization method using ImageJ/Fiji plugins such as JaCOP (Bolte & Cordelières 2006, J Microscopy) or DiAna (Gilles et al 2017, Methods), which are both user friendly and well documented. Plotting fluorescence profiles over single line scans might be a helpful additional way to display some of these data (e.g. the LDIP "rings" around the Nile Red/BODIPY bodies in Fig 2B).

RESPONSE: Better images have been provided in Figure 1A, 2 and 3 in the revised manuscript to more clearly indicate the obvious colocalization of proteins with LDs. In addition, colocalizations of fluorescence signals for LDAP1/3-Cherry and BODIPY were quantified in Figure 1A, based on an assessment of the Manders' co-occurrence coefficient, as described in the figure legend, and as suggested.

Reviewer #2:

The study from Pyc et al. focuses on the role of LDIP, LDAP3 and, SEIPIN1/2 in LD biogenesis. Previous works from the same lab consortium have demonstrated physical interactions between LDIP and LDAP3 or SEIPIN2/3. The present manuscript provides evidence of functional interactions between these partners. The authors nicely demonstrated, by manipulating the relative expression level of LDIP and LDAP1/3 or SEIPIN1 in leaves and seeds that these three proteins cooperate to control LD number and size. These experiments were carefully conducted including the analysis of gene expression by RT-PCR. They also demonstrated, using heterologous expression in insect cells, that LDAP3 is able to recruit LDIP to the LD surface. Conversely, they convincingly showed that LDIP is not required for the association of LDAPs and other known LD proteins with LDs. However, the results of the localizations of SEIPIN1/2 and LDIP raise some questions.

This work is remarkable because it demonstrates the importance of the stoichiometry of the three partners in the formation of LDs, which is a difficult task. The manuscript is nicely built and written.

Point 1. I have some concerns about the respective localizations of SEIPIN1/2 and LDIP, shown in Fig. 4.

First of all, the pattern of localization of GFP-SEIPIN1 and GFP-SEIPIN2 in N. benthamiana is different from the one observed by Cai et al. (2015) in the same transient expression system. Cai et al. have shown that GFP-SEIPIN1 and GFP-SEIPIN3 localized mostly to specific regions of the ER that might be LD forming sites. They also noticed that the expression of GFP-SEIPIN1/3 induced a dramatic reorganization of the ER to form discrete regions that mostly overlap with GFP- SEIPIN1/3 and LDs. Consistent with these results, Taurino et al. (2018) showed that SEIPIN overexpression reshapes the ER into perinuclear structures and vesicle-like bodies. In the present manuscript, the localization of GFP-SEIPIN1/2 displayed the typical reticular pattern of the ER. Thus, the overexpression of SEIPIN1/3 did not induce the reshaping of the ER that was previously observed in Cai et al. (2015) and Taurino et al. (2018). How do the authors reconcile such different results?

RESPONSE: Our results showing the co-localization of ectopically-expressed SEIPIN1/2 with LDIP at the ER network (Figure 4B) were conducted at relatively early time points after co-expression (i.e., 3-days post-infiltration) in order to help distinguish the localization of proteins between the ER and LDs. As mentioned by the reviewer, longer (ectopic) expression of SEIPIN in plant cells results in a dramatic reorganization of the ER, with very close association of ER and LDs (Cai et al. 2015; Pyc et al., 2017a; Taurino et al., 2018; Greer et al., 2020). The goal of this particular experiment (i.e., Figure 4B) was to show that the over-expression of SEIPIN resulted in the relocalization of LDIP from LDs to the ER. This relocalization is not as obvious when SEIPIN coexpression is allowed to go longer, as the ER and LDs become much more closely associated. However, we have added a new supplemental figure (see Supplemental Figure 4) showing that longer expression of SEIPIN1/2 (i.e., 5-days post-infiltration) does indeed reorganize the ER, as expected; note that our previous experiments with SEIPIN ectopically expressed in N. benthaniana leaves were also conducted at 5-days post-infiltration (Cai et al., 2015). As shown also in the new Supplemental Figure 4, the reorganization of the ER in SEIPIN overexpressing cells coincides with the dramatic aggregation of LDs, which, as mentioned, makes it more difficult to determine if LDIP is indeed associated with ER, or LDs.

Point 2. Moreover, it would be important to image GFP-SEIPIN1/2 without co-expressed Cherry-LDIP (and with FP-labelled ER and fluorescent-stained LDs), as a control. I think that providing 3D projections of surface rendering, high-magnified Z-stack images would help to better visualize the contact areas between LDs and the ER.

RESPONSE: Expression of GFP-SEIPIN2 without Cherry-LDIP is now included in Figure 5A. GFP-SEIPIN2 co-localized with an ER marker protein (EMP1-Cherry), showing a largely reticular pattern consistent with data shown in Figure 4B. We did not generate 3D projections or renderings of the ER-LD junction sites, as we have previously described these in detail in Cai et al. (2015) and Pyc et al. (2017a)..

Point 3. Then, I am not convinced that the co-expression of GFP-SEIPIN1/2 and Cherry-LDIP resulted in the relocalization of LDIP from LDs to the ER. Further evidence should be provided, e.g. by subcellular fractionation of SEIPIN1 OE and wt leaf extracts and, western blot analysis of LDIP content in the different subcellular fractions using anti-LDIP antibody (described in Pyc et al. (2017)). The relocalization of LDIP by SEIPIN overexpression might impair LDIP activity and could explain why the ldip KO and SEIPIN OE display similar LD phenotype. It is therefore important to confirm this observation.

RESPONSE: To determine whether the observed relocalization of LDIP to ER by co-expression with SEIPIN1 or SEIPIN2 (Figure 4B) might be due to a general impairment of LD biogenesis, we determined whether SEIPIN1 or SEIPIN2 might affect localization of a different LD coat protein, namely LDAH1. As shown in Figure 4C of the revised manuscript, when expressed on its own, LDAH1 localized specifically to LDs, as expected (Kretzschmar et al., 2020). Further, unlike LDIP, the localization of LDAH1 to LDs was unaffected by co-expression with SEIPIN1 or SEIPIN2 (see Figure 4C). These data support the premise that the alteration in LDIP localization from LDs to the ER upon co-expression with SEIPIN1/2 was due to a more specific and intimate relationship between these two proteins in plant cells.

Point 4. At last, I have some questions about the co-localization of LDIP and SEIPIN1 observed by BiFC in Fig. 4C. It seems to me that there is a co-localization of the signal produced by LDIP-SEIPIN1 interaction with that of the peroxisomes. Am I wrong?

> **RESPONSE: We apologize for the confusion regarding the usage of the peroxisomal marker protein (Cherry-Perox). This fusion protein was included in BiFC experiments to help identify transformed cells, which is important, since cells lacking a BiFC interaction show no fluorescence. The scaling used in the previous Figure 4C identified single cells, not subcellular organelles. Thus, the fluorescence pattern of the Cherry-peroxisome marker and BiFC signals appear to overlap. We have removed the Cherry-peroxisomal marker protein from the figure and described its usage in BiFC assays more clearly in the Methods (see 'BiFC and Y2H Assays' section) and in the figure legend for the new Figure 5C.**

Point 5. Moreover, the putative localization of LDIP-SEIPIN complex at ER-LD contact sites is important to investigate, so I suggest to mark the ER and LDs in this experiment.

> **RESPONSE: Thank you for this suggestion. We have included a series of higher-magnification images in the new Figure 5D showing that the BiFC fluorescence signal occurs in the immediate vicinity of MDH-stained LDs. Unfortunately, we unable to co-express an additional ER marker protein for technical reasons, i.e., the confocal microscope used in these experiments allows for imaging three fluorescent signals in cells, thus we could only visualize the BiFC signal, MDH, and, as mentioned above, Cherry-Perox serving as a cell transformation marker. Nonetheless, the well-known localization of SEIPIN to the ER and ER-LD junction sites (Cai et al., 2015; Brocard et al., 2017; Müller et al., 2017; Pyc et al., 2017b; Taurino et al., 2018; Coulon et al., 2020; Greer et al., 2020) strongly suggests that the protein interaction is occurring in a region of intimacy between the ER and LDs..**

Point 6. In the Discussion (from L 521), I think the arguments in favour of a similarity between Arabidopsis LDIP and human LDAF1 are not convincing. Using the Conserved Domain Database at NCBI to confirm the data shown in Supplemental Fig. 2, I found that the promethin domain is identified in LDAF1 but not in LDIP (LDIP contains only a DUF63 domain). Moreover, a BLAST analysis of plant proteins using human LDAF1 as a query identifies similarity with oleosins but not with LDIP, as mentioned in Eisenberg-Bord et al. (2018). In my opinion, part of the discussion (from L 518) and the model for the function of LDIP (Figure 8) based on this premise should be deleted, along with the Supplemental Fig. 2. Moreover, structural models shown in Fig. 8 are hypothetical and are not supported by the experimental results of the manuscript. It is a very interesting model that needs to be experimentally validated, at least partially, before publication.

> **RESPONSE: To obtain additional data that informs structural and functional similarities of plant SEIPIN/LDIP and human SEIPIN/LDAF1, we conducted a series of experiments based on recent observations in mammals and insects that identified a conserved hydrophobic helix (HH) in SEIPIN that binds to LDAF1 (Chung et al., 2019). Using this information as a guide, we showed that plant SEIPIN2 and LDIP interact in an HH-dependent manner. Specifically, LDIP was no longer redirected to the ER when co-expressed with SEIPIN2 lacking the HH region (SEIPIN2ΔHH) (Figure 5B), the BiFC signal between LDIP and SEIPIN2 was significantly reduced when the HH was deleted from SEIPIN2 (Figure 5C), and LDIP interacted with SEIPIN2 and a minimal construct containing just the SEIPIN2-HH region, but not SEIPIN2ΔHH, in a yeast 2-hybrid system. Collectively, these data strongly support a structural and perhaps functional similarity between plant SEIPIN/LDIP and human SEIPIN/LDAF1. Further, we conducted a more comprehensive analysis to explore the evolutionary relationships between LDIP and LDAF1 proteins. As mentioned in our response to similar issue raised by Reviewer 1, a new of our group with expertise in evolutionary biology (Dr. de Vries) curated animal LDAF1 and plant LDIP proteins and then used the resulting conserved-sequence alignments for pairwise comparisons of profile hidden Markov models searches (via HHpred) against the Arabidopsis and human proteomes. As shown in the new Supplemental Figure 6 and as described on pages 23 and 24 in the Discussion of the revised manuscript, these reciprocal searches recovered LDAF1 and an oleosin as the best hits. These results indicate a relationship between LD proteins of plants and opisthokonts that share a last common ancestor that likely lived more than 1.5 billion years ago (i.e., the last common ancestor of eukaryotes). This also warrants further investigation and speaks to a conspicuous similarity and perhaps reflects a deep homology of the components involved in LD form and function. However, convergence or even more complicated evolutionary scenarios cannot be ruled out.**

Reviewer #3:

This story from Pyc et al. is a follow up on a previous works of the same groups on LD biogenesis in plants. It highlights the cooperation of LDAPs, LDIP and seipins for proper LD assembly. Taking into consideration the recent finding on the role of the LADF1/Seipin complex in LD assembly initiation or nucleation, they propose a parallel mechanism involving LDAPs and LDIP in LD nucleation in plant cells. To reach their findings the authors employed several cell types to study the localization of the proteins in focus and the changes they induce in LD size distribution. Most conclusions of the manuscript were drawn from these two readouts. Overall the data are convincing but need further details. There remain major points to be clarified or addressed to meet standards for publication in TPC journal. In particular, interpretations on the LDIP/LDAP localization should be clarified.

Point 1. Because the LD size is a crucial readout, this referee believes that only clear cut differences in size can be interpreted with confidence.

RESPONSE: We appreciate this concern and we have softened the language throughout the revised manuscript with regards to our interpretations of the results for the relative differences in LD size distributions among the various plant lines examined. Unfortunately, it is not considered appropriate to do statistical analysis on the three LD size classes as defined in this study (i.e., <0.5 µm [small], 0.5-1.0 µm [intermediate], and >1.0 µm [large]), since the only statistics test available for these kind of data (i.e., parts of whole) is the Chi-squared test, which can only compare the observed results against expected results for the same line and not all the lines together (for example, in Figure 1B, WT vs ldip vs LDAP1-Cherry vs LDAP1-Cherry x ldip, etc). Nonetheless, we did perform statistical analysis on the average LD size (i.e., LD diameter) for each line examined using a Kruskal-Wallis test followed by a Dunnett post-hoc multiple comparisons test. Note that the results of these latter statistical analyses of average LD size are presented as violin plots in the new Supplemental Figure 1, which support the LD size distribution data presented as pie graphs in revised Figure 1B, 6A and 7. Similarly, the average LD size in the yeast cell lines examined are presented also as violin plots in the revised Figure 8C. Details on all of the statistical analyses performed in the study are presented in a new 'Statistical Analysis' section in the Methods and summaries of all the statistical analysis data are provided in the new Supplemental Dataset 1.

Point 2. Differences in LD number are always striking (statistical tests are actually only provided for these) and the data are solid.

RESPONSE: Thank you. As mentioned above, all the statistical analyses performed in the revised manuscript, including the differences in LD numbers and sizes in plant and yeast cells, are presented in a new 'Statistical Analysis' section in the Methods and summaries of all the statistical analysis data are available in the new Supplemental Dataset 1. Refer also to the new graphs in Figure 1B, 6A, 7 and 8B and 8C, as well as the related violin plots on average LD size (in plant lines) in Supplemental Figure 1.

Point 3. Localization studies are not obvious, especially in Fig 2. This referee would suggest to the authors to systematically zoom on selected regions so that the reader may appreciate the LD distribution and protein localization, which are important aspects of the paper.

RESPONSE: As suggested, we have provided more 'zoomed-in' (higher magnified) images of selected regions of the plant cells shown in Figures 1A and 3 and, as well as new higher quality images in Figure 2A showing the localization of transiently-expressing Venus-tagged LDAP3 or LDIP in insect cells stained with the neutral lipid-specific dye LipidTOX, rather than Nile red. We hope these revisions satisfy your previous concerns about LD distribution and protein localization.

Point 4. LDIP seems to be shaped like reticulons, which are curvature inducers. Is there any parallelism to make here? If yes, would the LDIP involved in curving the LD monolayer to ensure a rapid LD assembly, which would lead to smaller or normal sized LDs, as compared with LDIP KO? These points may deserve comments, especially since curvature have been recently brought up for LD biogenesis and tubular images are shown by these authors as the localization of LDIP-LDs (Figure 4B).

RESPONSE: This is a very interesting possibility and definitely one we will consider as we continue to study the functional role(s) of LDIP in LD biogenesis in plant cells. As suggested, we have commented on this in the revised paper – see Discussion section (pg 28). The new text reads: "Similar also to how LDAF1 is proposed to function

(Chung et al., 2019; Prasanna et al., 2021), the hydrophobic segments of LDIP could integrate into the ER bilayer in manner that promotes membrane-bending, akin to ER-shaping reticulon proteins."

Point 5. Figure 1: It seems that not all LDs are LDAP1/3 positive in both in WT and LDIP KO. In WT, some LDs belonging to the tinier population seems to lack LDAP.

RESPONSE: We apologize for the quality of the images in Figure 1 in the previous version of the manuscript, which were misleading in terms of our overall observations. We do not see any consistent evidence of subpopulations of LDs and their associated proteins in plant cells. That is, based on our experience working with numerous, ectopically-expressed plant LD (fusion) proteins, including LDAPs, oleosins, LDAH1 and LDIP in this study, they generally localize to all LDs in plant cells and, as such, any apparent non-colocalizations are usually the consequence of non-uniform staining of the LDs with neutral lipid-specific dyes, such as Nile red, and/or a relatively low(er) fluorescence signal attributable to an expressed LD (fusion) protein at smaller-sized LDs. As mentioned above, we have addressed this concern in the revised manuscript by providing new images of higher quality in Figure 1A (and Figure 2A and 3) that are more representative of what we typically observe in terms of the obvious colocalization of the LDAP1/3-Cherry proteins with BODIPY-stained LDs. In addition, colocalizations of fluorescence signals for LDAP1/3-Cherry and BODIPY were quantified in Figure 1A, based on an assessment of the Manders' co-occurrence coefficient, as described in the figure legend.

Point 6. I would thus recommend quantifying the fraction of LDs that are LDAP positive per LD size, since size matter.

RESPONSE: Please see comment above.

Point 7. Based on this observation, the conclusion that LDIP does not recruit LDAP1/3 should be reconsidered since LDIP could recruit LDAP to some LD population (those lacking LDAPs in LDIP KO). This illustrates the importance of quantifying the fraction of LD LDAP-negative in both WT and KO cells; if this fraction is somehow conserved, then the data would suggest that LDIP is not required for LDAP targeting.

RESPONSE: As mentioned, we do not see any consistent evidence of localization of proteins to subpopulations of LDs of different sizes. However, in Figure 2B, it is clear that co-expression of LDIP and LDAP3 in insect cells results in the formation of larger-sized LDs. We do not know if this change in size represents a true biological function of LDIP and LDAP3 proteins, or whether it's an artifact of the heterologous expression system. We have addressed this caveat by stating "Whether the apparent increase in LD size in insect cells co-expressing LDIP and LDAP3 (Figure 2B) reflects a biologically relevant function of these proteins in plant cells, or an artifact of the heterologous expression system, remains to be determined."

Point 8. Figure 2A: LDAP3-venus do not colocalize with all Nile red-positive structures, suggesting that it targets a subset of LDs only. Clarifying this will help. Also, the below displayed colocalization where the venus signal fills the LD interior, i.e. the Large LD "in the nucleus", is weird. A ring of venus signal should be observed as in figure B, given the large size of the LD (unless this large LD is an aggregate of smaller LDs). The authors should double check.

RESPONSE: We apologize for the relatively poor quality of this image. We have revised Figure 2A to show new images of the localization of transiently-expressed LDAP3-Venus to LDs, based on its colocalization with the neutral lipid-specific dye LipidTOX, rather than Nile red, which, as this reviewer mentions below, is not as good of a marker for LDs.

Point 9. Given the structure of LDIP with multiple hydrophobic helices, why doesn't it display a membrane signal instead of the cytosolic one that can be seen in figure 1A? In Figure 4B, mCherry-LDIP seems membranous.

RESPONSE: We do not have a definitive explanation as to why, given its hydrophobic characteristics (refer to hydropathy profile shown in Supplemental Figure 6B), ectopically-expressed LDIP expressed on its own localizes predominantly to the cytoplasm in insect cells, other than to suggest that the putative homologs of proteins that serve as LDIP-binding partners in plant cells, e.g., SEIPIN and perilipins, are not able to mediate its localization to the ER and/or LDs. Notably, LDIP ectopically-overexpressed in plant cells localizes to LDs and also accumulates in the cytoplasm (Pyc et al., 2017b; refer also to Figure 4B in this study). Similarly, human LDAF1 is mostly cytoplasmic when mammalian cells are cultured in regular media, but is LD-localized when cells were treated with oleic acid to stimulate LD formation (Castro et al., 2019). So, despite the hydrophobic nature of a portion of the LDIP protein, it apparently has capacity to remain in the cytoplasm rather than become membrane associated. Notably,

there is no structural information currently available for either LDIP or LDAF1, so we don't know if this hydrophobic region forms membrane-spanning domains or a 3D domain structure that is hydrophobic on the interior, but otherwise surrounded by regions that help solubilize the overall protein.

Point 10. The sole above image is supposed in A to demonstrate that LDAP recruits LDIp. This seems insufficient and the quality of the image does not allow to say so.

RESPONSE: We apologize again for the relatively poor quality of this image in the previous version of the manuscript. We have subsequently revised Figure 2A to show new images that better convey the intracellular localization of transiently-expressed Venus-tagged LDIP or LDAP3 (or LDAPΔC100) when expressed on their own in insect cells, as well as, for comparison purposes, Venus alone. Notably, in all of the new images in Figure 2A, LDs were stained with the neutral lipid-specific dye LipidTOX, rather than with Nile red (as in the previous version of this figure). The results showing the LD localization of transiently-expressed Venus- or Cherry-tagged LDIP in cells stably-expressing non-tagged LDAP3 (i.e., indicating the recruitment of LDIP to LDs by LDAP3) are now presented solely in Figure 2B.

Point 11. Still the signal of LDIP is cytosolic while this referee is expecting it to be membranous. Maybe the referee is wrong on this but it would be helpful if the author elaborate on it.

RESPONSE: Again, we apologize for the quality of prior images that suggested the presence of different subpopulations of LDs and associated LD targeting. We have replaced these images with images of higher quality and better representation, showing targeting to all (or the vast majority of LDs). While it is indeed possible that some other factor(s), such as simply the larger size of an LD, is recruiting LDIP to LDs, separate from an interaction with LDAP3, this model is significantly more complex than what we know from current and former experiments: LDIP was identified in a 2-hybrid screen using LDAP3 as bait, and a physical interaction on the LD surface was previously demonstrated using BiFC (Pyc et al. 2017). LDAP3 targets to LDs independently of LDIP (Figure 1A), and also targets to LDs in insect cells (Figure 2). LDIP is only associated with LDs when co-expressed with LDAP3 (Figure 2B), but not when co-expressed with a mutant of LDAP3 that no longer targets to LDs (Figure 2B). The simplest explanation is that LDAP3 is required for the location of the LDIP to LDs. We have softened the overall interpretation of our data by saying "localization" rather than "recruitment", in the modified conclusionary statement in the revised manuscript (pg 11): "… these results are consistent with those in Arabidopsis leaves and seeds showing that LDAPs can target to LDs independently of LDIP (Figure 1) and, further, that LDAPs are important for localizing LDIP to the LD surface."

Point 12. 5A: The number of LDs seemed to be only increased significantly, meaning more assembly events. The intermediate size, increased by 5-10% which make the referee wonder about the significance. The authors seem to give importance to this tiny change in the intermediate size. Thus they should provide evidence of its significance. From the display pictures, one can only appreciate the change in number.

RESPONSE: We agree that, based on the images present in Figure 6A, the increase in the number of LDs in leaves of the LDIP overexpression lines (renamed LDIP-1 and LDIP-2; see next comment) relative to WT is a much more predominant phenotype compared to the relative decrease in the intermediate-sized LDs, as depicted in the related graph. As such, we have softened the language in the text related to these observations (see pg. 17) to say: "However, overexpression of LDIP in two independent stable lines (LDIP-1 and LDIP-2) produced a near doubling in the total number of LDs in leaves and with a decrease in average LD size, including a trend towards an increased proportion of smaller-sized LDs at the expense of intermediate-sized LDs (Figure 6A and Supplemental Figure 1B)." In addition, the results of a statistical analysis supporting the significantly smaller average LD sizes in the LDIP-1/2 overexpression lines relative to WT are presented in the new Supplemental Figure 1B, which also support the LD size distribution data presented as pie graphs in revised Figure 6A. It is also worth noting that significantly smaller-sized LDs were also observed in Arabidopsis LDIP-1 seeds compared to LDs in WT seeds when analyzed by electron microscopy, as highlighted by the representative images and graph in Figure 6B. Refer also to the summaries of the statistical analysis data for Figure 6A and 6B in the new Supplemental Dataset 1.

Point 13. When looking at the figures shown in A and B, only the change in number is striking. When looking at the quantification of this figure, B, C, D, one can see changes in size. The author should clarify this by showing more convincing images in principal and supplement or better explain the display pictures and details that can only be seen upon quantification.

RESPONSE: As already discussed, we agree that, based on the images present in Figure 6A, the increase in the number of LDs in leaves of the LDIP overexpression lines is a much more predominant phenotype compared to the relative decrease in the intermediate-sized LDs, as depicted in the related graph. As such, we have revised the text related to these observations (see pg. 17) and we have provided the results of a statistical analysis supporting the significantly smaller average LD sizes in the LDIP-1/2 overexpression lines in the new Supplemental Figure 1B, which supports the LD size distribution data presented as pie graphs in revised Figure 6A. Similarly, we also note the significantly smaller-sized LDs in Arabidopsis LDIP-1 seeds, as highlighted by the representative electron micrographs in Figure 6B. Note that the 'observation index' graph previously presented in Figure 5C, as well as the graph depicting the percentage of supersized LDs in seeds previously presented in Figure 5D, have both been removed from the revised manuscript.

Point 14. With the existence of three seipin isoforms that these authors have shown, it could well exist a LD subpopulation formed by a similar mechanism involving other proteins, e.g. oleosins.

RESPONSE: This is a very good point and future work is no doubt required to understand the functional interactions of the three SEIPIN proteins in plant (Arabidopsis) cells, including their potential homo- and hetero-oligomeric interactions and with partners in LD biogenesis, including oleosins, LDAPs and LDIP. Note that we make a related statement in the final paragraph in the Discussion (pg 29) which reads: "While this model is consistent with the experimental evidence collected to date, many questions remain. For instance, do plant SEIPINs form oligomeric, radial structures similar to those observed in yeast, insects and mammals, and is the complex composed of mixtures of all three SEIPIN homologs, or are the complexes distinct? How does oleosin work together with SEIPINs, LDIP and LDAPs to facilitate LD biogenesis in plant cells?...."

TPC2021-RA-00378 1st Editorial decision – *revision requested* June 2, 2021

We have received reviews of your manuscript entitled "LDIP Cooperates with SEIPIN and LDAP to Facilitate Lipid Droplet Biogenesis in Plants." On the basis of the advice received, the board of reviewing editors would like to accept your manuscript for publication in The Plant Cell. This acceptance is contingent on revision based on the comments of our reviewers. Among their comments below, please also particularly consider the following: 1. include the additional result in the abstract, and 2. correct the wrongly cited references. Please highlight all changes and include a detailed annotation of changes of the text, with line numbers, and noting your responses to the comments.).

-- Reviewer comments:

TPC2021-RA-00378R1 1st Revision received June 2, 2021

Reviewer comments on previous submission and **author responses**:

Reviewer #1

Lipid droplets (LDs) store energy in seeds and other plant cells. LDs form from the ER and their cell biology is unique because they are surrounded by a lipid monolayer, rather than the bilayer that surrounds other organelles. Many proteins are required for LD biogenesis and stabilization: oleosins are critical in seeds, but are replaced by other proteins in different tissues. Previously, the authors and others have characterized several LD-associated proteins. In this manuscript, they draw on recent literature from LD formation in other organisms to generate hypotheses about how these LD-associated proteins interact during LD formation. The key findings from this work and others are summarized by the model in Figure 9. Although the interactions they document here provide insight into how LD formation is influenced by these protein interactions, it remains unclear how they interact to affect LD size. The authors have done an extremely through job of addressing my comments from my previous review of this manuscript and I agree with them that the document is dramatically improved. They have conducted more through analyses of their microscopy experiments and now present the data using appropriate graphs and micrographs. The proteomics data have been deposited in an appropriate database.

Point 1. The authors now present appropriate controls for the BiFC experiments, including the interesting result that the SEPIN version without the HH domain is insufficient to recruit LDIP to the ER. However, the images supporting

the BiFC experiment are a bit strange: Why is only the LDIP-SEPIN2 presented (and not LDIP-SEPIN2deltaHH)? Why is only one cell transformed? Why does the BiFC signal appear cytoplasmic? I think that my questions could be resolved by inclusion of additional BiFC images, either in the main figure or supplemental data.

RESPONSE: Since there is little to no BiFC signal attributable to co-expressed cCFP-LDIP and SEIPIN2DHH (refer to graph in Figure 5C), we do not show representative CLSM images of the cells from this experiment. However, for the images of cCFP-LDIP and nVenus-SEIPIN2 that are shown in Figure 5D, we have clarified that these micrographs are at high magnification and represent only a portion of an individual transformed N. benthamiana leaf epidermal cell. As such, the images presented in Figure 5D are meant to simply convey (as requested by a reviewer during the initial manuscript review) the aggregated ER and LDs observed in cCFP-LDIP and nVenus-SEIPIN2 co-transformed cells and how this is similar to results observed when Cherry-LDIP and GFP-SEIPIN1/2 were overexpressed in cells (refer to Supplemental Figure 4). We have revised the text in both the Results (see pg. 16) the legend for Figure 5D (see pg. 60) to help clarify this.

Point 2. I also advise the authors to focus their writing. Currently, the intro is probably longer than it needs to be (5 pages) and the discussion definitely is (almost 10 pages). While I appreciate that they are integrating many sources of information into their proposed model, I think the authors run the risk of losing non-specialist readers in so much detail.

RESPONSE: While we have shortened the text in a few places in the Introduction section, we chose not to make revisions or changes to the Discussion section, since we feel that the writing does well to tell a comprehensive and compelling story that includes enough details to provide context for non-specialist readers, but yet also gives important insights for more specialist readers. In support of this, none of the other reviewers (or the editors) requested shortening of the paper.

<u>Reviewer #2:</u>

The study from Pyc et al. explores in detail the role of LDIP in the biogenesis of lipid droplets (LDs), focusing on its functional and physical relationships with LDAP and SEIPIN, two other players of LD biogenesis. The present manuscript is a revised version that has been substantially improved over the original manuscript. Notably, the authors have introduced new experimental results that identify the binding domain of SEIPIN with LDIP. They indeed demonstrated that a hydrophobic helix (HH) domain on SEIPIN is necessary and sufficient for its interaction with LDIP. Moreover, appropriate controls are now included in BiFC and Y2H experiments, using a version of SEIPIN lacking the HH domain, thus clarifying any doubts on the interaction between SEIPIN and LDIP. In addition to this major improvement, the authors also included a control, using LDAH1 as a LD marker, to convincingly support that the relocalization of LDIP from LD to the ER is driven by its specific interaction with SEIPIN, and not due to a general alteration of LD biogenesis induced by SEIPIN overexpression. Overall, these additional evidences are supporting the proposed model of LD biogenesis, which was mostly hypothetical in the previous version of the manuscript.

Point 1. In my opinion, the revised document fully deserves publication in The Plant Cell, after three minor corrections. The abstract should mention the results that have been added to the new version of the manuscript, in particular concerning the identification of the binding domain of SEIPIN with LDIP.

RESPONSE: As suggested, the Abstract has been revised to include text indicating that the interaction of LDIP with SEIPIN is dependent on the conserved hydrophobic helix sequence in SEIPIN.

Point 2. References: In line 155, the reference Berthelot et al., 2014 (Berthelot, K., Lecomte, S., Estevez, Y., and Peruch, F. (2014). Hevea brasiliensis REF (Hev b 1) and SRPP (Hev b 3): An overview on rubber particle proteins. Biochimie 106, 1-9) seems more appropriate than Berthelot et al., 2016.

In line 261, the reference Noi et al, 2007 is not appropriate as it described the use of a fluorescently labeled phospholipid but not a neutral lipid dye. Several LipidTOX reagents are indeed available from Thermo Fisher Scientific; the one used in the reported experiments is probably the HCS LipidTOX{trade mark, serif} Deep Red Neutral Lipid Stain. This reference should be deleted and the dye correctly named in line 1057.

RESPONSE: The Berthelot et al. (2016) reference has been replaced with the suggested Berthelot et al. (2014) reference. The Noi et al. (2007) reference has been removed and the LipidTOX reagent used in this study has been

correctly named as "HCS LipidTOXTM Deep Red" in both the Results (when it was first mentioned) and in the Methods (see Microscopy section).

Reviewer #3:

The authors have done a great job in repeating their experiments and providing much better images, which support their conclusion better. They have addressed most of this referee's major questions. Therefore, this referee is in support of the publication.

TPC2021-RA-00378R1 2nd Editorial decision – *acceptance pending* **June 9, 2021**

We are pleased to inform you that your paper entitled "LDIP Cooperates with SEIPIN and LDAP to Facilitate Lipid Droplet Biogenesis in Plants" has been accepted for publication in The Plant Cell, pending a final minor editorial review by journal staff. At this stage, your manuscript will be evaluated by a Science Editor with respect to its presentation of scientific content, compliance with journal policies, and presentation for a broad readership.

Final acceptance from Science Editor **June 26, 2021**
